# Supplementary material for: Depth-enhanced high-throughput microscopy by compact PSF engineering
Source: Nat Commun. 2024 Jun 7;15:4861. doi: 10.1038/s41467-024-48502-y (PMC11161645; doi:10.1038/s41467-024-48502-y)
Supplement: Supplementary file 1 — Supplementary Information [file 41467_2024_48502_MOESM1_ESM.pdf]

Depth-enhanced high-throughput microscopy by compact PSF engineering

Nadav Opatovski[1,*], Elias Nehme[2,3,*], Noam Zoref[2], Ilana Barzilai[2], Reut Kedem Orange[1], Boris Ferdman[1], Paul Keselman[4], Onit Alalouf[2], Yoav Shechtman[1,2]

[1]RBNI Nanoscience Institute, The Technion, Haifa, Israel; [2] Faculty of Biomedical Engineering, The Technion, Haifa, Israel; [3] Faculty of Electrical and Computer Engineering, The Technion, Haifa, Israel; [4] Sartorius Stedim North America, Inc., Bohemia, NY, USA

[*] These authors contributed equally to this work.

Supplementary Information

Contents

Supplementary note 1 - EDOF and standard PSF deconvolution 2
Supplementary note 2 - Coherence length verification for incoherence EDOF 2
Supplementary note 3 - Phase element axial positioning 3
Supplementary note 4 - Quantification of DOF increase of incoherence EDOF 4
Supplementary note 5 - Comparison of phase-mask EDOF to NA reduction 4
Supplementary note 6 - CellSnap dataset preparation 4
Supplementary note 7 - Image normalization 5
Supplementary note 8 - Neural network architectures 6
Supplementary note 9 - Training details 6
Supplementary note 10 - Post-processing segmentation outputs 7
Supplementary note 11 - Quantification of segmentation quality 7
Supplementary note 12 - Imperfect labels and axial reconstruction accuracy 7
Supplementary note 13 - Comparison to the standard PSF 8
Supplementary note 14 - Acquisition and post-processing times 8
Supplementary note 15 - Three-dimensional tracking 8
Supplementary table 1 - PSFs summary 9
Supplementary Figures 10
Supplementary references 22

Supplementary note 1 - EDOF and standard PSF deconvolution

We have taken advantage of the low variability of the EDOF PSF to improve imaging results. We did so by implementing Lucy-Richardson deconvolution[1,2] and subsequently assessing its efficacy on images of the spheroid presented in Fig. 3. The iterative deconvolution process requires an input image and a PSF model. We managed to apply 2D deconvolution to EDOF images using a single focused PSF kernel for the entire image, because the EDOF PSF stays approximately focused and constant along an extended axial range. For comparison, we repeated the process for standard PSF, which suffers from strong axial PSF variations across the 3D object being imaged. For appropriate modelling of the PSFs, we initially imaged z-stacks of fluorescent microspheres on a coverslip for both the EDOF PSF and the standard PSF. Each PSF model was designed to optimally fit the corresponding imaged PSFs. Respectively, the EDOF focused PSF was modeled as a Lorentzian-inspired function:

$$I(x,y) \propto \frac{1}{(x-\mu)^2 + (y-\mu)^2 + \gamma^2}, \qquad (S1)$$

with $\mu = 12$ and $\gamma = 0.8$. Simultaneously, the standard focused PSF was modeled as a Gaussian function:

$$I(x,y) \propto \exp\left(-\left(\frac{(x-\mu)^2}{2\sigma^2} + \frac{(y-\mu)^2}{2\sigma^2}\right)\right), \qquad (S2)$$

with $\mu = 12$ and $\sigma = 0.6$. In both cases, the PSF model kernels were cropped to a size of 23X23 pixels around their centers. For the deconvolution process, we used the Matlab native *deconvlucy* function. For all images, the function input included a background-subtracted spheroid image and a corresponding PSF model, both normalized by their Euclidean norms. The number of iterations was set as 5. This value was selected as a trade-off between a number of iterations too low to achieve significant improvement of resolution, and an excessively high number of iterations that results in artifact generation.

As expected, the EDOF deconvolution process yielded an improved resolution EDOF image over an extended axial range, as the PSF used for the deconvolution was appropriate for an extended axial range. As for the standard PSF deconvolution, the resolution was not improved far from focus due to the inadequacy of the PSF model (Supplementary Fig. 1).

Supplementary note 2 - Coherence length verification for incoherence EDOF

The incoherence EDOF relies on separating the back-focal-plane (BFP) of the optical system into several non-interfering annular zones. As each corresponds a fraction of the imaging NA, they naturally exhibit a larger depth of field than the full-aperture PSF. An optical element producing this effect comprises of transparent steps along the radial direction, creating concentric rings of different layer heights, where each step introduces an optical path difference (OPD) greater than the fluorescence coherence length, namely, $L_c \ll \Delta nh$, where L_c is the fluorescent emission coherence length, Δn is the difference between the refractive index of the element and the surrounding medium (usually air), and h is the layer height. In our home-made element, we created the steps by stacking 170 µm thick microscope coverslips with concentric holes. Prior to making the optical element out of 170 µm thick glass, we had to verify that the coherence length of the fluorescence is indeed significantly shorter than the optical path difference (OPD) contribution of each layer. We built a Michaelson interferometer, with a pinhole and a lens at its entrance. A camera at the end of the interferometer measures two overlapping images of light that passed through the pinhole (Supplementary Fig. 2). To measure the coherence length of the beads (Fluoro-max, ThermoFisher Scientific; Cat #G0100), we prepared a dense, bright sample on a coverslip, from which we

induced fluorescence using an inverted microscope (Ti-2, Nikon). We placed the pinhole of the interferometer in the emitted light path, letting the fluorescence pass through the interferometer. The scanning mirror of the Michaelson interferometer slowly moved across 20 µm, while the camera was measuring the intensity of the overlapping images. In total, 20 µm of mirror travel accounts for an OPD of 40 µm due to path extension towards and from the scanning mirror.

Supplementary note 3 - Phase element axial positioning

Often in optical systems, shift independence of the PSF is desired, especially when performing PSF engineering. This requires placement of the phase element at the Fourier-plane of the system, which generates FOV-independent PSF modulation. At planes other than the Fourier plane, the spatial support of wavefronts emanated from different points in the FOV are translated from one another. Thus, they experience a translated phase profile, leading to a spatially varying PSF. Additionally, in real imaging systems imperfections generate a finite amount of native FOV dependence, independent of intended phase modulations. For this reason, analyzing the FOV dependence of a complex phase mask (e.g. Tetrapod or EDOF) will not clearly reveal the amount of FOV dependence due to misplacement of the element (relative to the native FOV dependence). Therefore, we used a simple calibration element to estimate the FOV dependence by the optical addition. We used a circular glass coverslip with a black ink dot drawn near its center. With the glass in place, we imaged beads highly defocused, such that a dark spot is clearly visible near the center of each defocused PSF. If the element is not correctly positioned in the Fourier plane, the position of the spot relative to the PSF will change with the position of the bead.

We verified the effect of element position over the FOV dependence by acquiring two images – one with the element at the exit of the objective (similar to how the phase elements in this work were positioned, Fig. 1), and one with the element placed ~20 mm below that plane (Supplementary Fig. 3A). Analysis was based on finding how much the dark dot near the center of the PSF drifts as a function of FOV position, in each image. For each bead, the outer PSF perimeter and inner spot perimeter were marked manually to find their center positions. Per PSF, the parameter we care about is the difference between these centers. In a FOV-dependent optical system, this parameter will demonstrate a trend related to the position of the PSF in the FOV. Supplementary Fig. 3F shows a quiver plot, with arrows denoting the deviation of the dark spot from the center of the PSF. Arrow lengths are enhanced for clarity and the mean deviation was reduced from all values (due to the ink dot is not being exactly at the center of the aperture). The arrow lengths and directions are randomly distributed across the FOV, governed by the finite precision of marking the circles, without a FOV-dependent trend. From this, we conclude that the position of the element does not introduce significant FOV-dependence. For comparison, we repeated the experiment with the ink dot placed at a wrong plane, about 20 mm below the objective. The results are shown in Supplementary Fig. 3C,E,G. This time, the position relative to the PSF *is* FOV dependent, leading to the arrows pointing in the radial direction, with radially increasing lengths. In total, approximately 60 PSFs were analyzed in each image. Viewing a histogram of the deviation magnitudes (Supplementary Fig. 3H), we see that at the correct plane, the deviations are rather small – typically below 2.5 pixels, with an average of 1.27. This is due to the finite precision of allocating the centers of the spots. On the other hand, when the PSF suffers from FOV dependence due to a poor axial position of the ink dot, the deviations are much greater, with an average of 2.3 pixels.

Supplementary note 4 - Quantification of DOF increase of incoherence EDOF

DOF quantification was performed over an ensemble of isolated beads suspended gel and imaged under the same conditions as the experiment. The PSF of the beads in gel was analyzed using statistical experimental data. We did so by acquiring a z-stack of another, sparse sample of beads in gel (10x sparser than the dense one), at steps of 1 µm. We analyzed the PSF of all isolated beads, by fitting a 2D gaussian to 80 frames around the focal plane. The average PSF widths as function of defocus are plotted in Supplementary Fig. 4A. Often, the DOF of a PSF is defined according to the broadening of the PSF, with the minimal PSF size as a reference. Such a comparison would not provide the full picture, as the minimal size of the standard PSF is smaller than that of the EDOF PSF. It may be that the "out-of-focus" size of the standard PSF is still smaller or comparable to the "in focus" EDOF PSF size. To provide the full picture, we examined the axial extents in which the PSF size is smaller than a given value. For a set of values spanning 1 to 4 µm, we calculated the standard DOF (SDOF) and EDOF defined as the axial extent in which the PSF is smaller than each value. The factor of DOF improvement by the EDOF PSF is obtained, per value, by dividing the EDOF by the SDOF. The improvement factors are presented in Supplementary Fig. 4B. The improvement reaches a plateau at a value of 1.9, from a PSF width of 1.7 µm onwards. As described in the main text, DOF quantification was performed over an ensemble of isolated beads suspended gel, under the same conditions as the experiment. The only difference was a lower bead density, for a better isolation of the PSFs.

Supplementary note 5 - Comparison of phase-mask EDOF to NA reduction

Supplementary Fig. 5 shows x-z projections of a coverslip-adhered fluorescent bead, obtained using three modalities – the native objective PSF, EDOF (lithography mask) and a reduced NA with DOF comparable to the DOF of the EDOF. The NA was reduced by placing an aperture of 6 mm at the exit of the objective (a 3D printed element similar to the mask holder). All projections are presented with the same lookup table. The total intensity measured with the EDOF PSF is 82% that of the standard PSF, while the total intensity measured with the reduced NA is only 8% that of the standard. The significant intensity reduction due to NA restriction is due to light blocking both in the illumination and detection optical paths. By measuring the excitation light intensity at the sample plane, we detected a reduction of 8% in total excitation light intensity due to the holder of the EDOF mask alone (17% with the mask in place), and of 53% by the 6 mm aperture block. while some of the detected photons of the phase-mask EDOF PSF is not localized in the PSF center (see logarithmic colormap), the phase-mask EDOF is an order of magnitude more efficient than the naïve NA reduction. It is more than an order of magnitude larger when only considering the center of the PSF.

Supplementary note 6 - CellSnap dataset preparation

To acquire a training/testing dataset we imaged 4 plates of 96 wells, with each well mostly containing a single spheroid. Each well was scanned in z twice: once with a standard objective lens and once with an objective lens inducing the Tetrapod PSF. To enrich the training dataset acquired from only 4 plates, we experimentally "augmented" the spheroid in each well by shaking the entire plate and re-imaging the wells multiple times. After shaking, the 3D spheroid is rested in a different "view" from before with respect to the objective (see Supplementary Fig. 7B), hence, a new shell of cells was acquired, constituting another training example.

To automatically curate the acquired experimental data we performed multiple filtering operations on the standard PSF channel (Supplementary Fig. 6C). First, we disregarded wells where the cells either didn't

form a spheroid, formed multiple attached spheroids, or formed highly amorphic and non-spherical shapes. The filtering criterion for judging whether cells formed a spheroid was based on the amount of pixels above a sample-specific intensity threshold. We first estimated the mean background intensity by averaging the lower 30% counts. Afterwards, we checked whether at least 500 cells (approx. 10x10 pixels in xy each) persist for more than 10 z slices with an average intensity of 5 times the mean background. To check whether the resulting shape is approximately a sphere, we projected the stack to xy using mean projection in z, cropped the spheroid and centered it, and calculated the ratio of the major axis divided by the minor axis of the enclosing ellipse. If this ratio was above 1.25, we disregarded the data from that well. Second, we disregarded wells where the acquired data was either too saturated or had extremely low signal-to-noise ratio (SNR). In a high-throughput setting, it is difficult to optimize the exposure time for all wells simultaneously, hence the intensity range can vary significantly. Data with more than 5% saturated pixels per z slice was discarded. Similarly, data with very low SNR either due to low labelling efficiency, or due to non-optimal exposure time was also discarded. To decide whether a stack has a low SNR or not, we first aligned consecutive slices using StackReg[3] and estimated the focus plane of the lower part of the spheroid (middle 75x75 pixels of the image) using the variance of the Laplacian filtered stack. The slice with the maximal variance was estimated to be the focus position. Stacks with a maximal Laplacian variance below 100 were deemed to have low labelling efficiency. In addition, we also calculated the ratio of the top 10% counts divided by the bottom 10% counts at the focal slice. If this ratio was below 1.8, we discarded the data due to low cell SNR.

Next, for stacks that passed all filters, we processed the accompanying Tetrapod channel. Like the standard PSF, we first cropped the data, centered it, and used StackReg to align consecutive slices. The focus of the lower part of the spheroid was also similarly found using the variance of the Laplacian filtered stack. Afterwards, the two channels were globally aligned by co-registering the bottom part of the spheroid using StackReg on the corresponding focal slice of each. The resulting dataset of stack pairs (Standard/Tetrapod PSFs) were aligned both spatially (in xy) and in focus (z), albeit the acquisition.

Finally, the standard PSF stack was processed with Cellpose[4] to produce a GT 3D segmentation label (Supplementary Fig. 6D). For training the segmentation model we discarded instance-level cell labels and thresholded the result of Cellpose to produce a binary volume. In addition, since Cellpose as most cell segmentation algorithms, was trained on data from either a confocal or a light sheet microscope, the resulting segmentations were heavily elongated in z, due to the resolution of the standard PSF (Supplementary Fig. 6E). Therefore, we post-processed the result by shrinking the segmented cells such that their axial extents are roughly only twice as large as their lateral extents.

Supplementary note 7 - Image normalization

Prior to training, we normalized count values of experimental data such that network input (2D slice) is first mapped to the range [0,1] using the 0.5 and the 99.95 percentiles respectively, and then z-scored by subtracting the mean and dividing by the standard deviation of the normalized counts. Network output for the focus finder is also normalized by linearly mapping the foci to the range [-1,1]. During training of both models, we randomly sampled a focus position and in addition to the normalization mentioned earlier, with a 50% chance we employed a contrast augmentation to the snapshot by raising the normalized values to a random power gamma in the range [0.7,1.3]. This enabled our models to be robust to focus position and global intensity variations observed in between wells.

Supplementary note 8 - Neural network architectures

Both the focus finder and the 3D segmentor constituting Cell-Snap are based on the fully convolutional U-net architecture[5,6]. Both U-nets have the same structure and are comprised of a 5-level encoder-decoder structure with skip connections. At each resolution the input features are processed by two convolutional layers with C 3x3 filters, where C is 64 in the first and second levels and is doubled at each consecutive level such that the number of features per level in the encoder is given by (64, 64, 128, 256, 512). Each convolution layer is followed by instance normalization[7] and a LeakyReLU activation with a negative slope of 0.1. Spatial resolution between levels is halved using 2x2 MaxPooling layers. Symmetrically, in the decoder the number of features is decreased in a mirrored fashion. Spatial resolution is increased through upsampling by a factor of 2 using Transpose convolution with a filter size of 2 and a stride of 2 like in the original u-net implementation. The main difference between the architecture of the focus finder and the 3D segmentor is in the input/output layers. For the focus finder, the input image is comprised of one channel which is the measured snapshot. As for the output, we pass the u-net output features through a global average pooling layer followed by a 1x1 convolution to reduce it to a scalar with no further activation (Fig. 4 main text). On the other hand, for the 3D segmentor the input image has two channels: the first channel is the measured snapshot, and the second channel is the predicted focus which is reshaped into an image to condition the segmentation on the focus position. As for the output, the channel dimension plays the rule of depth, such that feature maps at the output layer are passed through a sigmoid activation and the result is treated as a 3D binary segmentation volume with 50 voxels discretizing a z-range of 200 microns (i.e., an axial voxel size of 4 µm).

Supplementary note 9 - Training details

Cell-Snap training was done in two separate stages. First, we trained the focus finder separately using pre-aligned z-stacks (Fig. 4 main text). In each training step, the input to the focus finder was a slice from the z-stack at a random depth and the regressed output is the normalized slice index. Training of this model was done on full images, since the task of predicting the focus position in z requires aggregating global spatial cues of the focused ring within the spheroid snapshot and relating the radius of this ring to the overall spheroid diameter. The loss function used for this task of scalar regression was the standard Mean Squared Error (MSE). In the second stage of training, we reshaped the output of the focus finder to a full image, concatenated it to the experimental snapshot, and fed the two channeled image into the 3D segmentor trained to output a 3D binary segmentation volume of 50 different depths (Fig. 4 main text). The 3D segmentor was trained using the Dice Loss, where the GT labels for training were obtained by post-processing the output of Cellpose from the experimental standard PSF z-stack. Unlike, the focus finder here we trained on patches and not full images, since the required information (shape/depth cues) to output the 3D segmented cells is mainly local and not global. Note that in the second stage of training the pre-trained focus finder parameters were kept fixed. Both models were trained with a batch size of 32 samples (either full images or patches) using the Adam optimizer with an initial learning rate of $\alpha = 0.001, \beta_1 = 0.9, \beta_2 = 0.999$, and a decoupled weight decay with $\lambda = 0.01$[8]. The learning rate was reduced by a factor of 10 if the validation loss didn't improve for 5 epochs, with a minimal learning rate of 0.00005. Training was stopped early if the validation loss didn't improve for 20 epochs. On a workstation equipped with a Titan RTX GPU training of both networks (focus finder + 3D segmentor) took roughly 7 hours.

Supplementary note 10 - Post-processing segmentation outputs

The outputs of Cell-Snap were thresholded at a confidence level of 0.2 and then passed through watershed splitting with ImageJ 3D Suite[9] to separate touching binary predictions (Supplementary Fig. 7A-D). Finally, cells with less than 100 voxels were discarded and each connected component was assigned a distinct label defining a cell instance. We note that in the rare event of adjacent cells aligning exactly with the principal axis of the Tetrapod PSF, the reconstruction accuracy was reduced due to inaccurate post-processing (Supplementary Fig. 7E-F). However, such cell layouts are extremely rare, and typically this loss of accuracy is confined to a small number of cells if any in each spheroid.

Supplementary note 11 - Quantification of segmentation quality

To quantitatively evaluate performance, we used two different metrics: the dice score which is a measure of the global segmentation quality, and the average precision, which is a measure of the individual cell (instance) segmentation quality. The dice score was computed on a per-voxel basis, where True Positives (TP) were predicted occupied voxels that overlap with ground truth occupied voxels, False Positives (FP) were predicted occupied voxels that do not overlap with ground truth occupied voxels, and False Negatives (FN) were ground truth occupied voxels that were predicted to be empty. Then, the dice score per volume was defined as

$$Dice = \frac{2TP}{2TP + FP + FN}. \qquad (S3)$$

As for the average precision metric, it required defining matches between predicted individual cells (after post-processing) and ground truth individual cells (prior to thresholding). First, each cell was matched to a ground truth cell that was most similar as predicted by the voxel-wise Intersection over Union (IoU). Then, the predictions were evaluated at a range of IoU thresholds; at a lower IoU threshold, fewer pixels of a predicted cell must match a corresponding ground truth cell for a match to be considered valid. Like the dice score, the valid matches defined the TP, predicted cells with no valid matches defined the FP, and ground truth cells with no matches defined the FN. Then, the average precision per volume was defined as

$$AP = \frac{TP}{TP + FP + FN}, \qquad (S4)$$

Note that in terms of the computer vision literature this definition does not follow the one from instance segmentation algorithms, where the average precision amount to averaging the precision (true positive rate) at different model thresholds covering the precision-recall curve, and then averaging the result over a range of IoU thresholds to produce the mean average precision or mAP. Here, we followed the definition from Cellpose[4,10], previously also defined as “accuracy” in StarDist[11,12], to be consistent with previous works in the field of cell segmentation.

Supplementary note 12 - Imperfect labels and axial reconstruction accuracy

The labels we were able to derive from applying Cellpose[4,10] to the z-stack acquired with the standard PSF were not perfect (Supplementary Fig. 8). In some cases, Cellpose failed to detect cells that are apparent in the image, either due to saturated regions, or due to densely packed cells (Supplementary Fig. 8A-H and Supplementary Fig. 9B). In other cases, part of the cells from the spheroid’s edge started detaching/moving in between the z scan with the standard PSF and the snapshot with the Tetrapod PSF (Supplementary Fig. 8I and Supplementary Fig. 11H). Both issues resulted in incorrect overlap

calculations rendering our performance worse than it is in practice. In addition to these factors, in other cases our reconstruction was slightly shifted in the axial direction (Supplementary Fig. 9G-H,J). Both quantitative metrics we used to quantify performance are extremely sensitive to such inaccuracies in the axial direction. However, for a wide range of applications, small inaccuracies in z (2.5% of the z-range) are bearable and could suffice in practice.

Supplementary note 13 - Comparison to the standard PSF

The labels derived with Cellpose were mainly reliable in the lower part of the spheroids, and hence our quantitative analysis was restricted to the middle 150x150 pixels of the image roughly capturing the lower quarter of the spheroid (Supplementary Fig. 10D, red square). As can be seen in Supplementary Fig. 10, CellSnap trained on snapshots of the standard PSF achieved impressive performance when the focus was set optimally (Supplementary Fig. 10E left column), although the signal from defocused cells appeared weak and diffuse. However, as the focus varied away from optimum, the performance quickly degraded (Supplementary Fig. 10D, F left column). On the other hand, when we complemented CellSnap with the Tetrapod PSF (Supplementary Fig. 10D-F right column), the achieved results were roughly similar for in focus imaging (Supplementary Fig. 10E, right column), but significantly more robust to focus changes, resulting in a more graceful decay in performance across a wide axial range of ~100 μm (Supplementary Fig. 10D-F right column).

Supplementary note 14 - Acquisition and post-processing times

Our acquired dataset consisted of stack-pairs with a 4 μm step in the axial axis. Comparing the acquisition time in this setting, would lead to z-stacks of 50 slices for the standard PSF to cover the 200 μm range. Meaning, CellSnap with a single snapshot exhibits a speed-up of 50x in acquisition speed and roughly 40x in post-processing when compared with Cellpose. However, the biological sample in hand may not strictly require such a fine axial sectioning, meaning the data acquisition for Cellpose analysis may be relieved. In our data we observed that cell nuclei had a diameter of ~20 μm. A sufficient axial segmentation of the space for CellPose would require capturing the nuclei at least at 2 axial planes, meaning a z-stack with a step size of 10 μm could suffice. As the signal-efficient range of our Tetrapod PSF in the experiment was roughly 120 μm, in main text Fig. 5.G, we timed our method compared to Cellpose analyzing z-stacks with 12 planes. In conclusion, compared to a naïve implementation of Cellpose, our result provides a speed up of roughly 10x in acquisition, and the same ratio in post-processing. Please note that in this analysis we left out the time required for training set acquisition and model training because these are one-time efforts that need to be done only once. The resulting model can then be applied to thousands of samples seamlessly, enjoying the speed up benefits mentioned above.

Supplementary note 15 - Three-dimensional tracking

We have demonstrated 3D nanoparticle tracking analysis (NTA) using the Tetrapod PSF. To evaluate the contribution of PSF engineering, we also performed the experiment with the standard PSF. Notably, the objective had a severe spherical aberration present, which extends the DOF and provides axial information to the PSF, better than the non-aberrated PSF. Localization in the time-lapse with both the standard and the Tetrapod PSF was performed with DeepSTORM3D. We used the default training parameters, with the signal to noise ration and particle density set to match the experimental data. After training, at test time we used a threshold of T=40 and a radius of 6 voxels for post-processing. The resulting tracks were linked using a custom code on a frame-by-frame basis. In between successive frames, the linking was performed using the Hungarian (munkres) algorithm with a distance threshold of 20 μm laterally and 30 μm axially.

The linked tracks were then thresholded prior to mean square displacement (MSD) calculations, such that only tracks with more than 50% localizations were used (Supplementary Fig. 12 and Supplementary Fig. 13). After MSD calculation, the diffusion coefficient and the localization precision were derived by extrapolating a linear fit to the first 4 datapoints, such that[13]:

$$\mathrm{MSD}(\tau) = a\tau + b \qquad (S5)$$

and

$$D = \frac{a}{2}$$
$$\sigma = \sqrt{\frac{1}{2}\left(b + a\frac{\Delta t}{3}\right)}, \qquad (S6 - S7)$$

where $\Delta t = 0.4$ (s) is the exposure time accounting for motion blur. The results in Supplementary Fig. 12 demonstrate that the Tetrapod PSF improves the overall number of recovered tracks, the mean track length, and the axial localization precision compared to the application of DeepSTORM3D without further PSF engineering.

At high particle densities, the Tetrapod PSF starts to overlap due to its large lateral footprint (Supplementary Fig. 13). Nonetheless, it still achieves a similar performance compared to the Standard PSF when considering tracks where the particle was detected in at least 50% of the frames (Supplementary Fig. 13E-F). Furthermore, if we use a stronger threshold of 70% (Supplementary Fig. 13G-H), the Tetrapod provides a significantly higher localization precision in Z even at high particle density where other PSFs[14] (that are also compatible with our approach) are known to be superior.

Supplementary table 1 - PSFs summary

The following table summarizes the phase masks (PSFs) used per experiment.

Mask	PSF type	Fabrication method	Objective	Emission channel	Used in experiments
EDOF 1	EDOF	Photolithography	10X	Green	Spheroids EDOF (Fig. 3)
EDOF 2	EDOF	Layers of cut glasses	10X	All	Beads in gel (Fig. 2)
Tetrapod 1	Tetrapod	Photolithography	10X	Green	CellSnap (Figs. 4-5)
Tetrapod 2	Tetrapod	Photolithography	20X	Red	NTA (Fig. 6)

Supplementary Figures

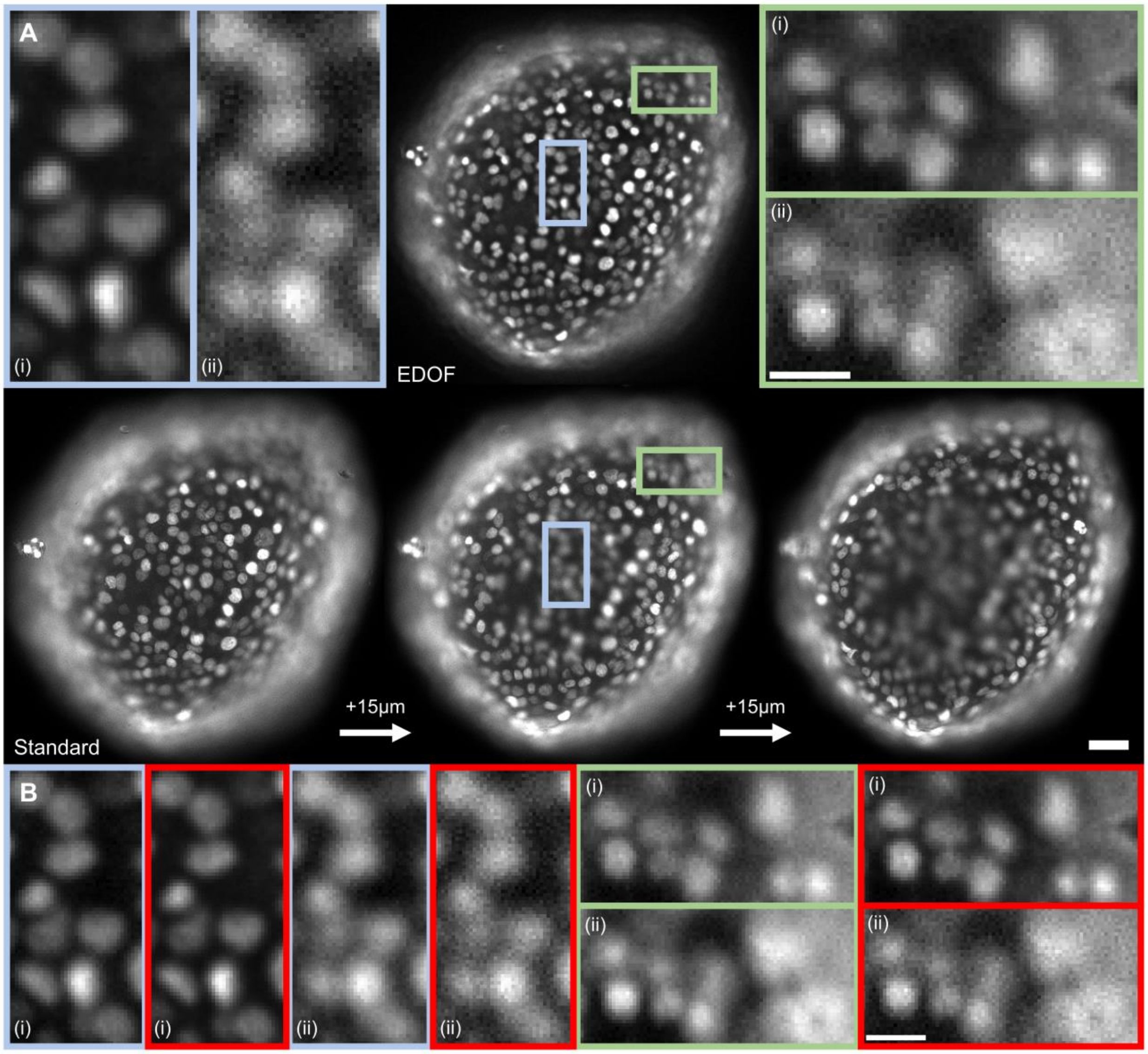

Supplementary Figure 1 – Improvement of EDOF imaging results by applying Lucy-Richardson deconvolution. **A.** Top row – a single deconvolved frame with the EDOF PSF, and zoom-ins, similar to Fig. 3. Bottom row – three standard PSF images, after deconvolution, at three different defocus values. Zoom-ins: (i) – EDOF, (ii) – Standard. Scale bar of the large images is 50 µm. Zoom-in scale bar is 20 µm. **B.** Comparison between zoom-in images before and after deconvolution. Deconvolved images are positioned to the right of the corresponding original image, and marked in red. (i) – EDOF, (ii) – Standard. Scale bar is 20 µm.

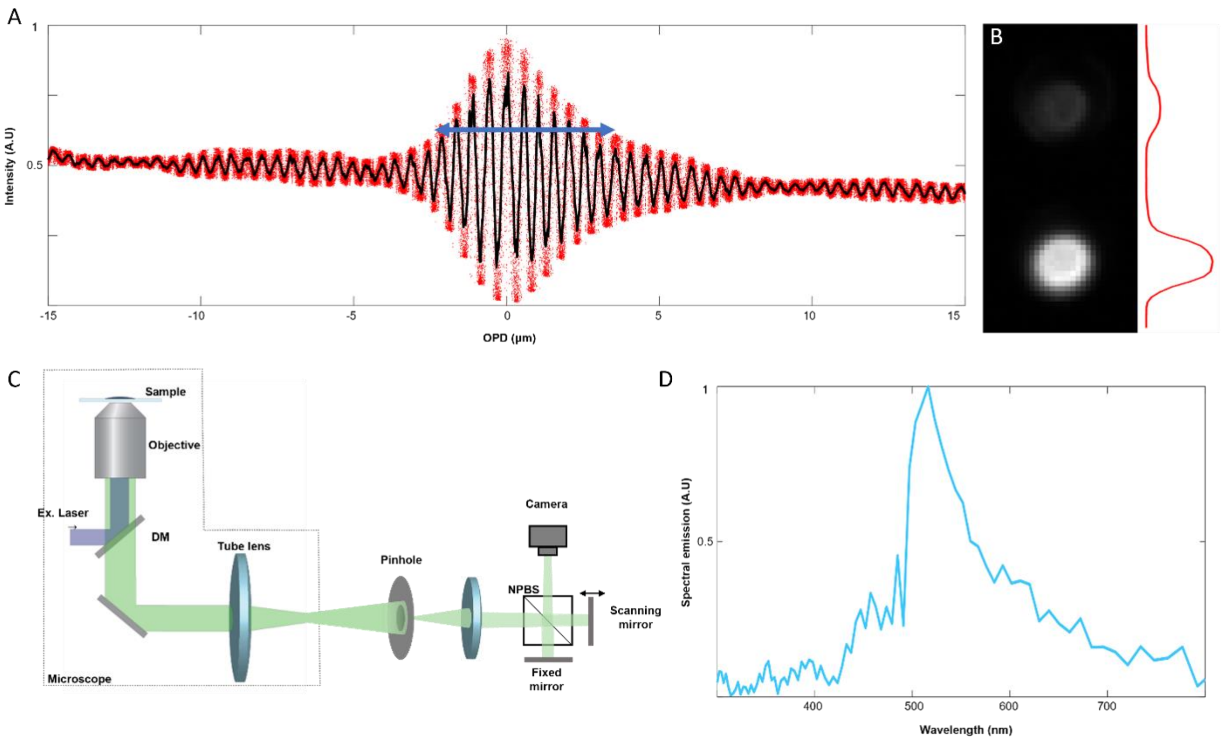

Supplementary Figure 2 - Coherence length measurement of the fluorescence of the beads imaged in Fig. 4. **A.** Interferogram of the fluorescence. **B.** Example pinhole images with destructive and constructive interferences. **C.** A schematic of the optical system used. Abbreviations: DM – Dichroic mirror, NPBS – nonpolarizing beamsplitter. **D.** The emission spectrum, as calculated from the interferogram. The spec emission was not available by the manufacturer, but the emission profile corresponds the expected result in terms of peak wavelength, shape and width. Source data are provided as a Source Data file.

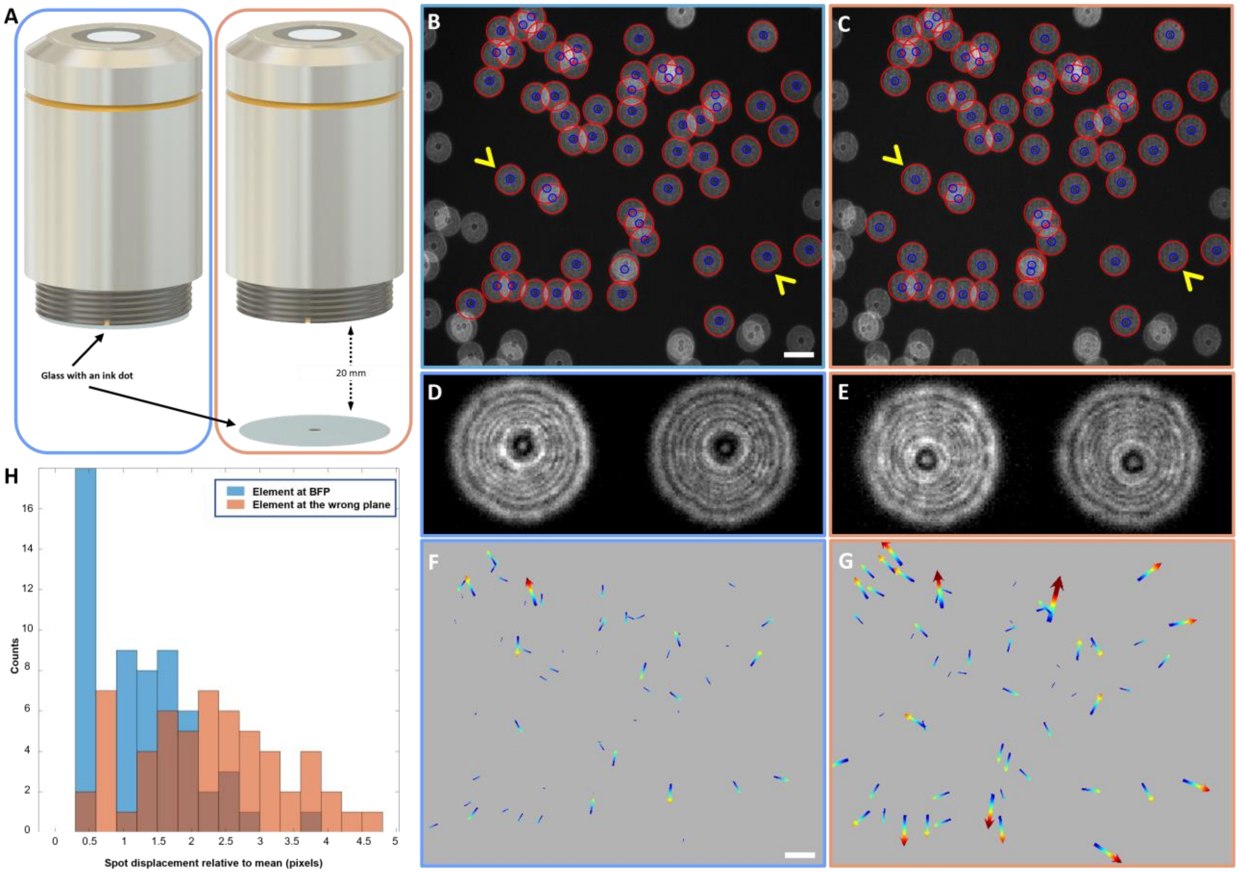

Supplementary Figure 3 - FOV-dependence effect due to optical element axial position. **A.** Experimental setup. A glass with an ink dot drawn at the center is placed beneath the objective. Left side – the dot is positioned at the objective exit, at the same axial position where we place the phase elements. Right side – the dot is positioned 20 mm below. **B.** Defocused beads imaged with an ink dot at the exit of the objective. Spot position and outer PSF position were marked (blue and red circles) to estimate the drift of the spot position relative to the PSF. Scale bar is 100 µm. **C.** The same as B., with the ink dot 20 mm below the correct plane. **D-E.** Close-up of the beads marked with yellow arrows from B., C., respectively. D. shows no change in the dark spot positions, while E. does, demonstrating FOV dependence. **F.** Quiver plot of the spot deviation, calculated from B. Arrow direction and sizes are independent of FOV position. **G.** Quiver plot of the spot deviation, calculated from C. Here, a radial trend is apparent. **H.** Distributions of the spot deviation magnitudes, in pixels. Source data are provided as a Source Data file.

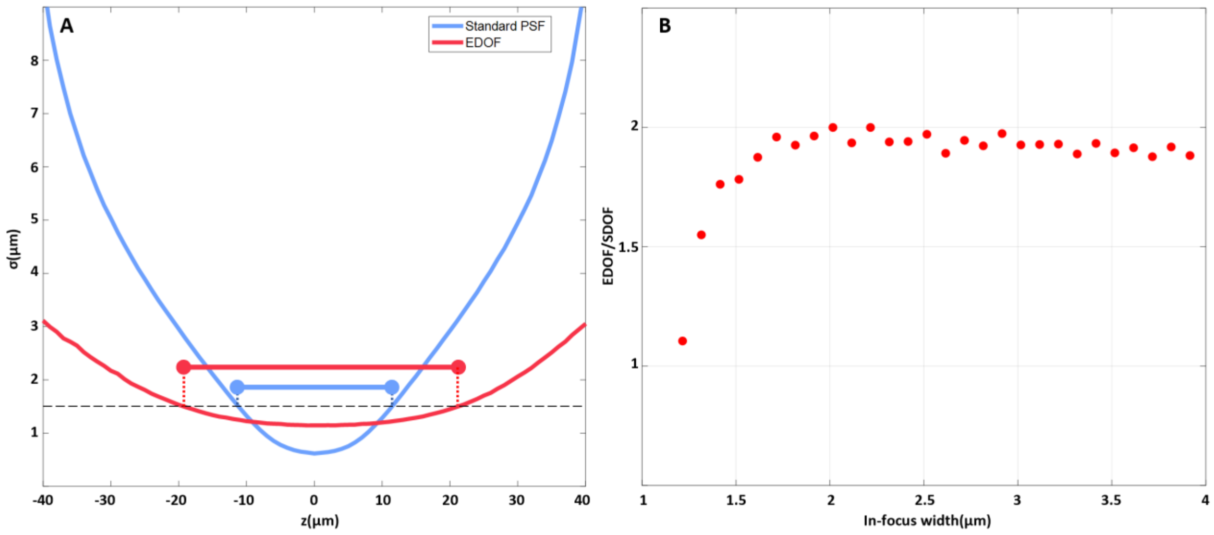

Supplementary Figure 4 - DOF comparison between the standard PSF and the incoherence EDOF, obtained from beads in gel. **A.** Standard PSF and EDOF PSF width as function of defocus (same curves as shown in main text Fig. 2.B). Horizontal lines are added as example of DOF calculations, for a DOF defined by PSF size ≤1.5 μm. **B.** Fractional DOF extension (ratio of EDOF divided by SDOF), as function of maximal "in-focus" PSF width. Source data are provided as a Source Data file.

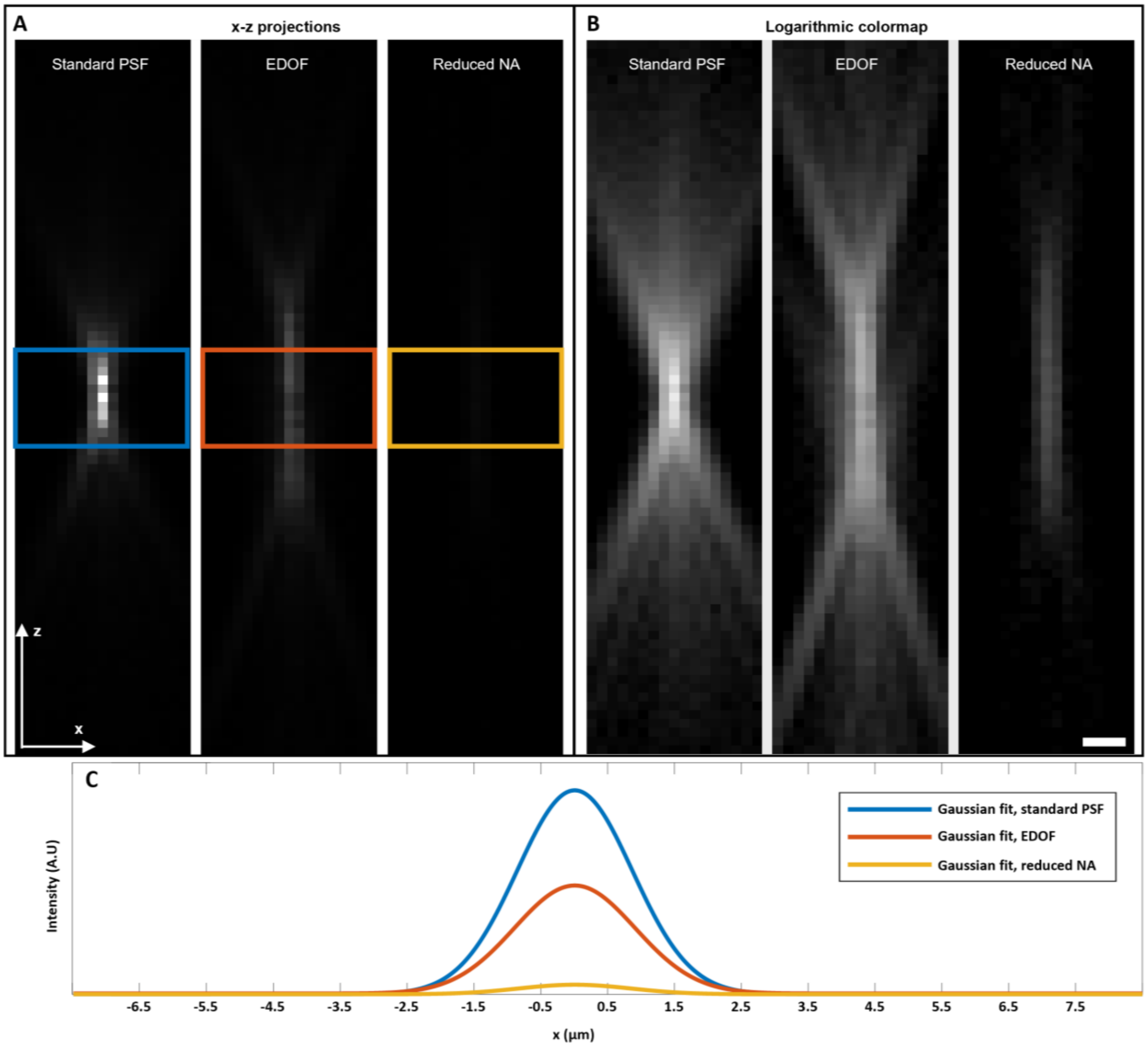

Supplementary Figure 5 - Comparison of phase-mask EDOF with the standard PSF and an NA reduction providing a similar DOF, in terms of photon efficiency. **A.** x-z projections. Aspect ratio is not unity for visibility – pixels account for 2 µm vertically and 1.17 µm horizontally. **B.** A logarithmic color scale of the projections, to better observe the PSFs. Scale bar is 5 µm. **C.** Averaged gaussian fit results of the regions marked by rectangles in **A**.

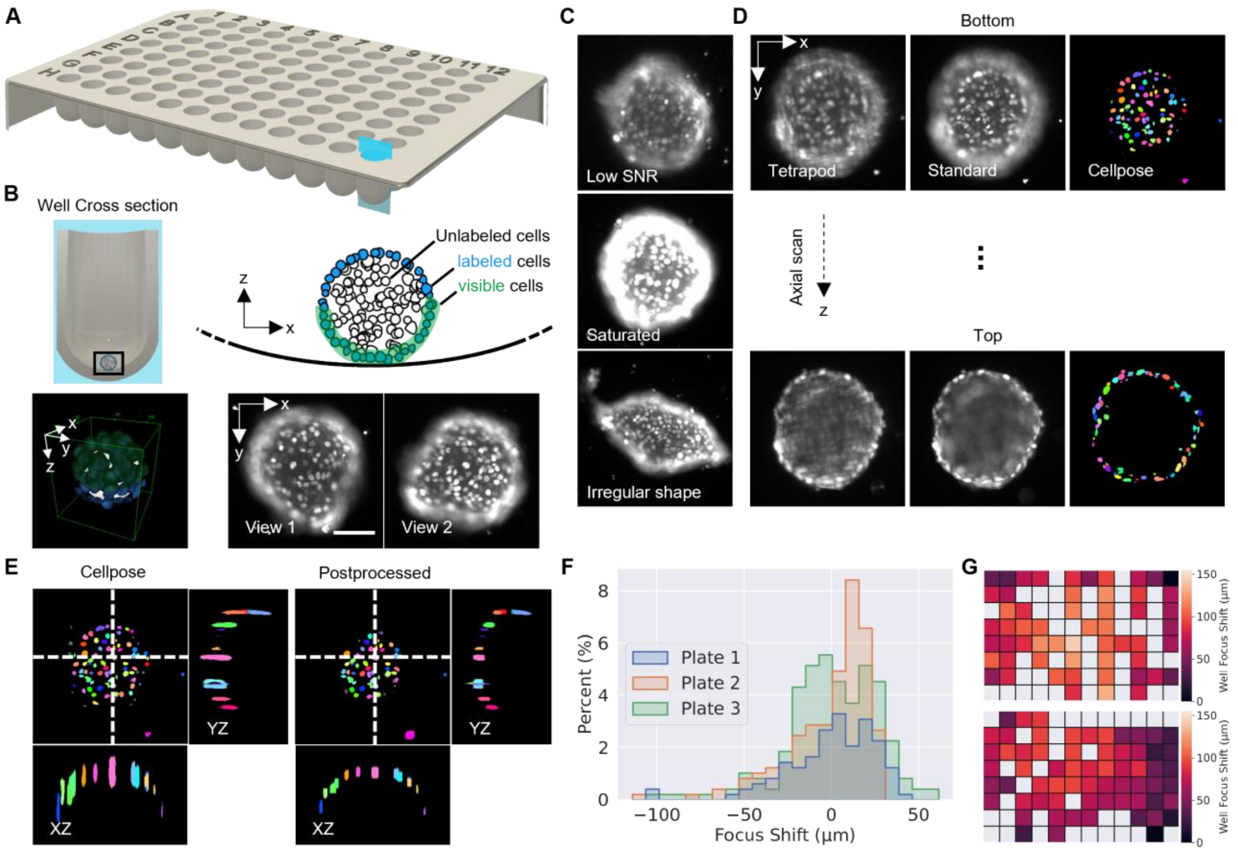

Supplementary Figure 6 - Training set acquisition and preparation. **A.** 96-well plate used for growing and imaging spheroids. **B.** The labelling/signal efficiency were such that signal is observed only from the outer shell of cells with scattering effects increasingly stronger beyond the middle point. Shaking the spheroids in between scans allowed us to acquire multiple "views" of cells from each well. Scale bar is a 100 μm. **C.** acquired data was filtered to discard saturated, irregular, or low SNR spheroids. **D.** The remaining stacks were aligned using StackReg (see text) and segmented with Cellpose. **E.** Cellpose segmentations were post-processed to reduce their Z extent. The result is a labelled training set of Tetrapod snapshots at different focus settings and their corresponding 3D segmentation. **F.** Focus shift per-well across 3 different plates. Source data are provided as a Source Data file. **G** Two different plate scans, where each well is colored according to its recovered focus by post-processing a standard PSF z-scan. Empty (white) wells refer to filtered data (e.g. as in **C**). Source data are provided as a Source Data file.

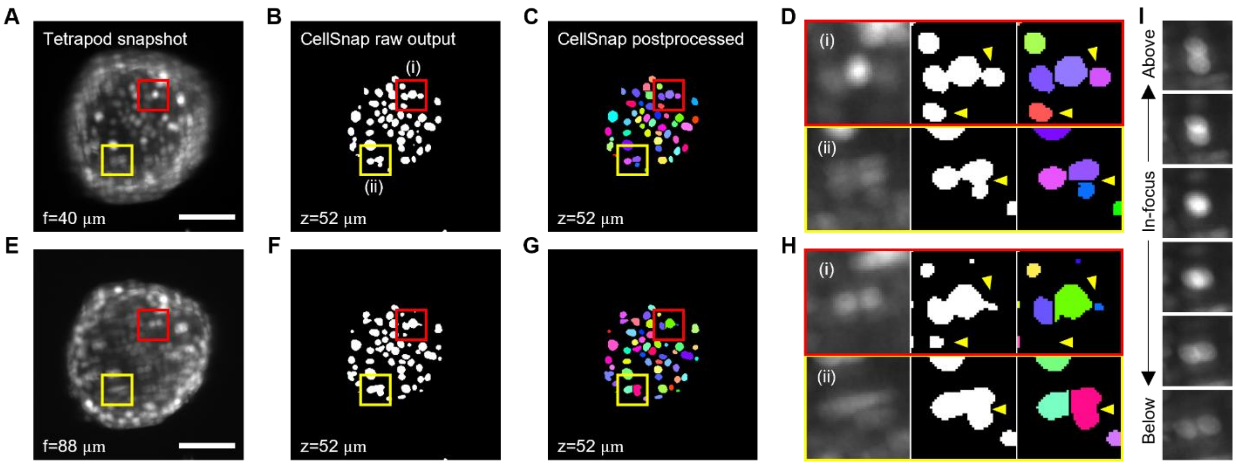

Supplementary Figure 7 – Post-processing role and directional reconstruction accuracy. **A.** Tetrapod snapshot at a focus position of 40 μm. **B.** CellSnap raw output at a depth of z=52 μm. **C.** Final output after postprocessing. **D.** Zoom-ins on the red and yellow squares in **A**-**C**. Yellow triangles mark touching cells separated after watershed post-processing. **E.** Tetrapod snapshot of the same spheroid at a focus position of 88 μm. **F.**-**G**. CellSnap output before and after postprocessing using the snapshot from **E**. **H.** Zoom-ins on the red and yellow squares in **E**-**G**. Yellow triangles mark touching cells not separated after watershed post-processing due to spatial proximity in the direction of the Tetrapod principal axis. **I.** Single cell imaged with the Tetrapod PSF at different focal positions for reference.

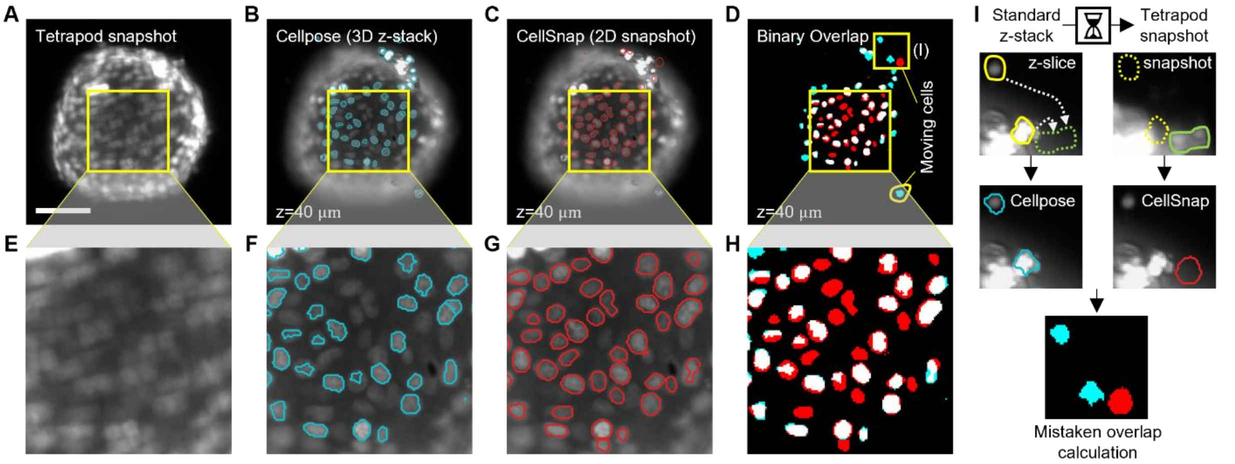

Supplementary Figure 8 – Imperfect labels by Cellpose. **A.** Tetrapod snapshot. Scale bar is 100 µm. **B.** Cellpose segmentations overlaid on top of the raw axial slice at z=40 µm. **C.** CellSnap segmentations overlaid on top of the raw axial slice at z=40 µm. **D.** Binary overlap of Cellpose (cyan, **B**) and CellSnap (red, **C**) segmentations; matches (true positives) are plotted in white. **E.**-**H.** Zoom-ins on the large yellow square in **A**-**D**. **I.** Zoom-in on the small yellow square in **D**. Due to cell movement in between acquiring the z-stack with the standard PSF and the Tetrapod snapshot (dashed white arrows), the reconstruction of Cellpose and CellSnap do not align, leading to a mistaken overlap calculation.

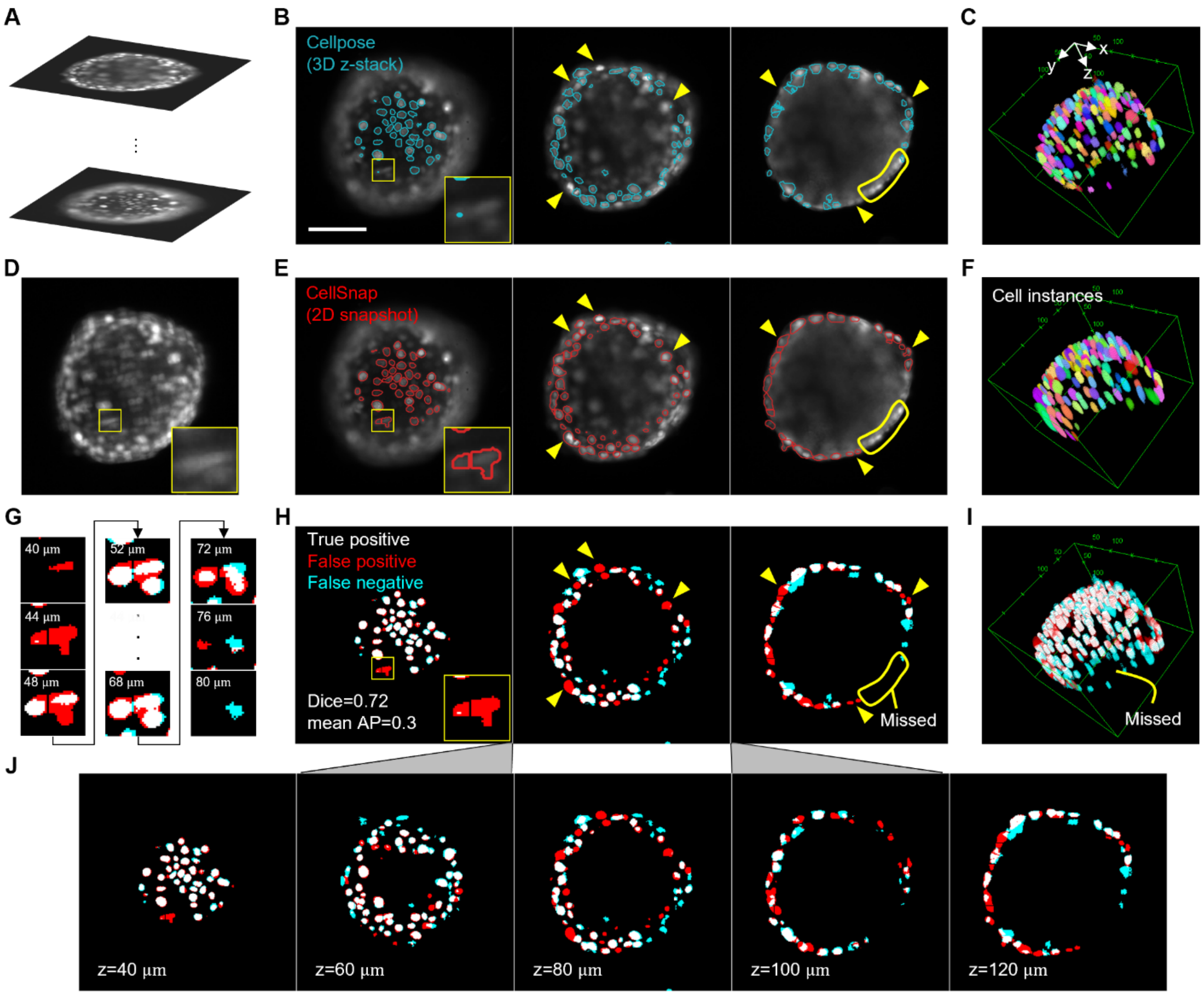

Supplementary Figure 9 – Reconstruction accuracy in Z. **A.** Standard PSF z-stack. **B.** Cellpose segmentations overlaid on top of raw axial slices separated by 40 µm. Scale bar is 100 µm. **C.** Cellpose individual cell instances rendered in 3D. **D.** Tetrapod snapshot roughly in the middle of the axial range. Yellow triangle marks a detaching cell going out of the axial range. **E.** CellSnap segmentations overlaid on top of raw axial slices separated by 40 µm. **F.** CellSnap individual cell instances (after watershed post-processing) rendered in 3D. **G.** Unrolled z-slices of the reconstructed cells in the yellow rectangle from **E**. **H.** Binary overlap of Cellpose (cyan, **B**) and CellSnap (red, **E**) segmentations; matches (true positives) are plotted in white. Yellow triangles highlight correct segmentations identified as false positives (red) either (i) due to imperfect labels, or (ii) due to inaccurate axial position. **I.** Binary overlap 3D rendering. **J.** Binary overlap in **H** explored at 20 µm jumps in z.

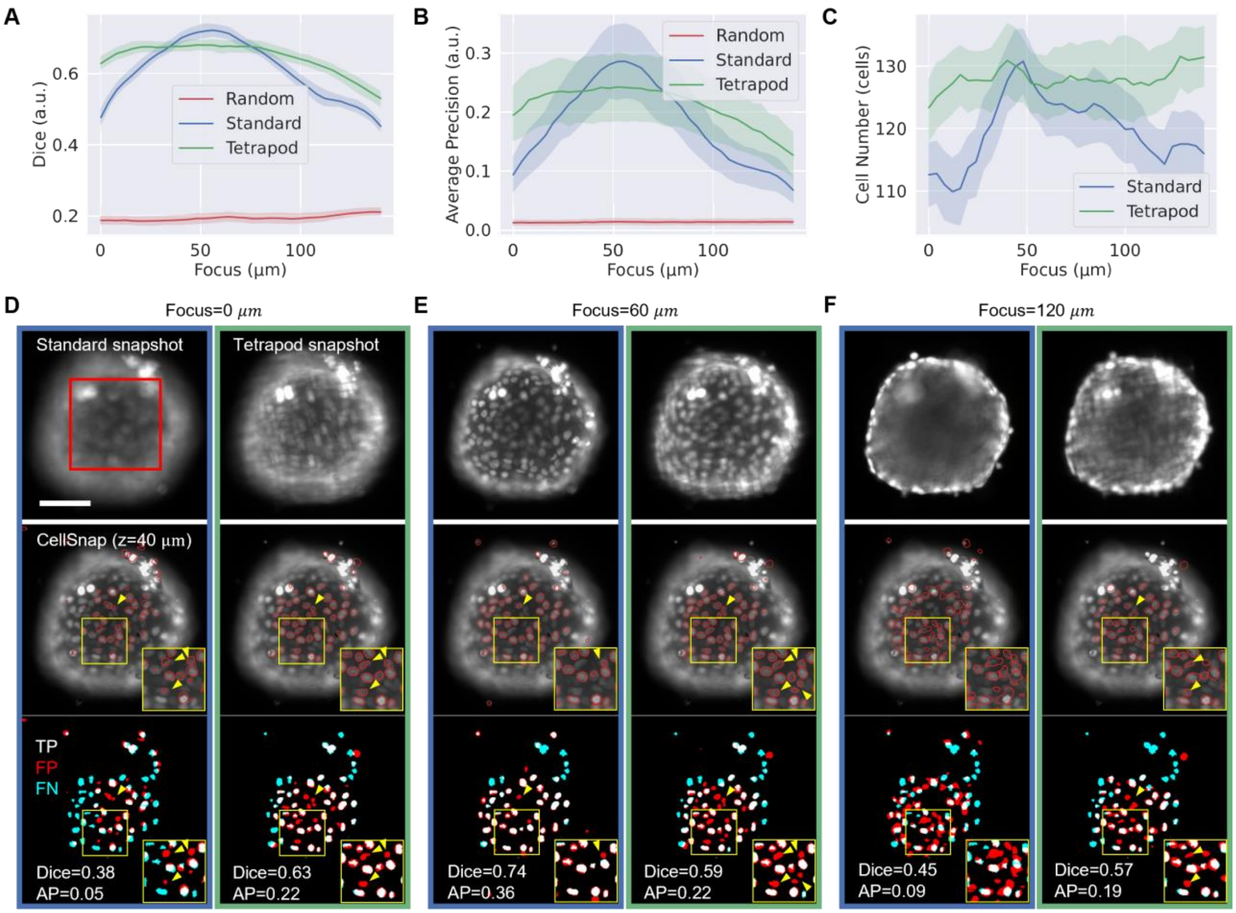

Supplementary Figure 10 - Comparison to CellSnap without PSF engineering. **A.** Dice score as a function of focus. **B.** (mean) Average precision as a function of focus. **C.** Number of cells recovered by CellSnap as a function of focus, using either a standard (blue) or a Tetrapod (green) PSF snapshot. In **A**.-**C**. data is presented as mean value +/- 95% confidence interval (i.e., 1.96*SEM). **D.** Visual example at a focus of 0 μm. Left column (blue) depicts the standard PSF snapshot (top), segmentations of CellSnap using this snapshot at a height of 40 μm (middle), and the binary overlap of CellSnap result (red) with the segmentations of Cellpose (cyan) derived from the respective z-scan. Red square marks area considered for quantitative comparison. Yellow triangles mark CellSnap segmentations mistakenly marked as false positives due to imperfect labels. Right column (green) depicts the equivalent Tetrapod snapshot (top), CellSnap segmentations (middle), and binary overlap with Cellpose segmentations (bottom). **E.** Similar to **D**, showing the same spheroid with a focus setting of 60 μm. **F.** Similar to **D** and **E**, with a focus setting of 120 μm. Source data are provided as a Source Data file.

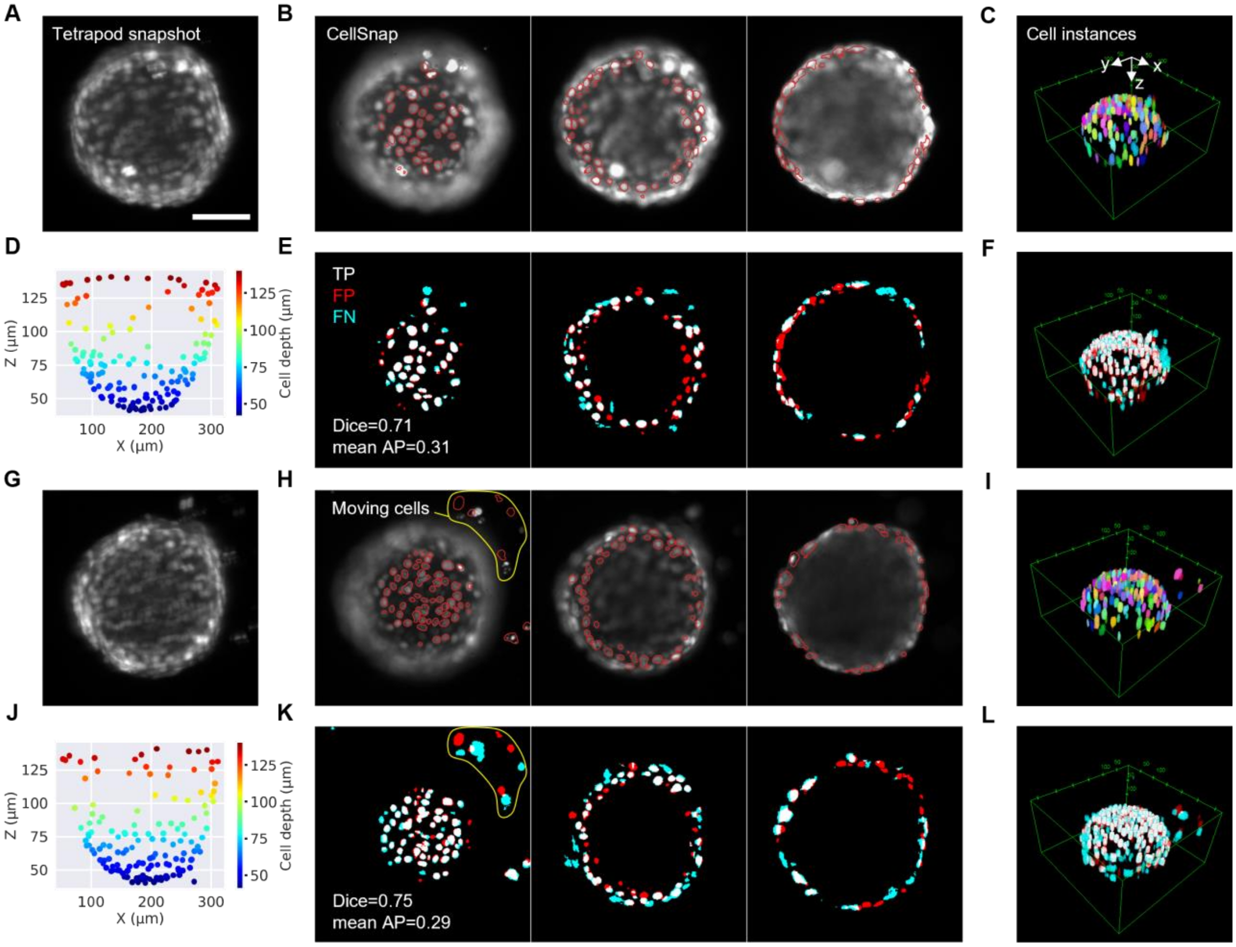

Supplementary Figure 11 - Segmentation examples. **A.** Tetrapod snapshot. Scale bar is 100 μm. **B)** CellSnap segmentations overlaid on top of raw axial slices separated by 40 μm. **C.** CellSnap individual cell instances rendered in 3D. **D.** XZ cross section plot of recovered cell centroids in 3D, colored by their z position. **E.** Binary overlap of Cellpose (cyan) and CellSnap (red, **B**) segmentations; matches (true positives) are plotted in white. **F.** 3D rendering of binary overlap in **E**. **G**-**H.** another example in a similar layout to **A-F.** Yellow area in **H** marks moving cells that result in a shifted prediction in **K-L**. Source data are provided as a Source Data file.

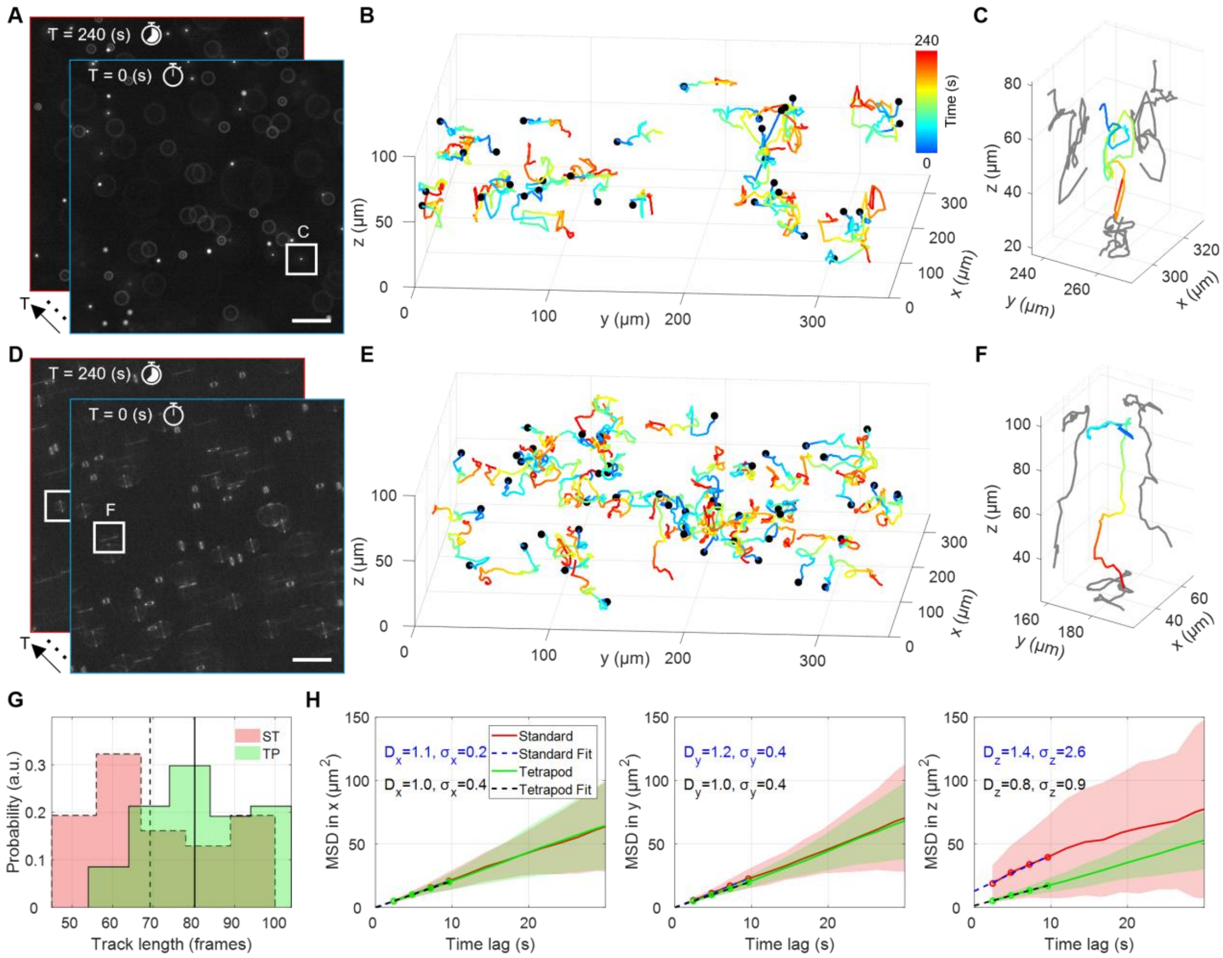

Supplementary Figure 12 - Comparison to 3D tracking without PSF engineering. **A.** Time lapse of snapshots with the Standard PSF. Scale bar is 50 μm. **B.** Recovered 3D tracks spanning 240 seconds with a 2.4 second resolution. **C.** 3D track corresponding to the highlighted white square in **A**. **D.** Time lapse of snapshots with the Standard PSF. Scale bar is 50 μm. **E.** Recovered 3D tracks spanning 40 seconds with a 0.4 second resolution. **F.** 3D track corresponding to the highlighted white square in **D**. **G.** Track length distribution in frames (where 100 frames = 240 seconds). Solid and dashed black lines marks the mean track length for the standard and the Tetrapod PSFs respectively. **H.** Ensemble mean square displacement (MSD) per axis. Filled area marks 1 standard deviation. D and σ are the per-axis estimated diffusion coefficient and localization precision respectively. Source data are provided as a Source Data file.

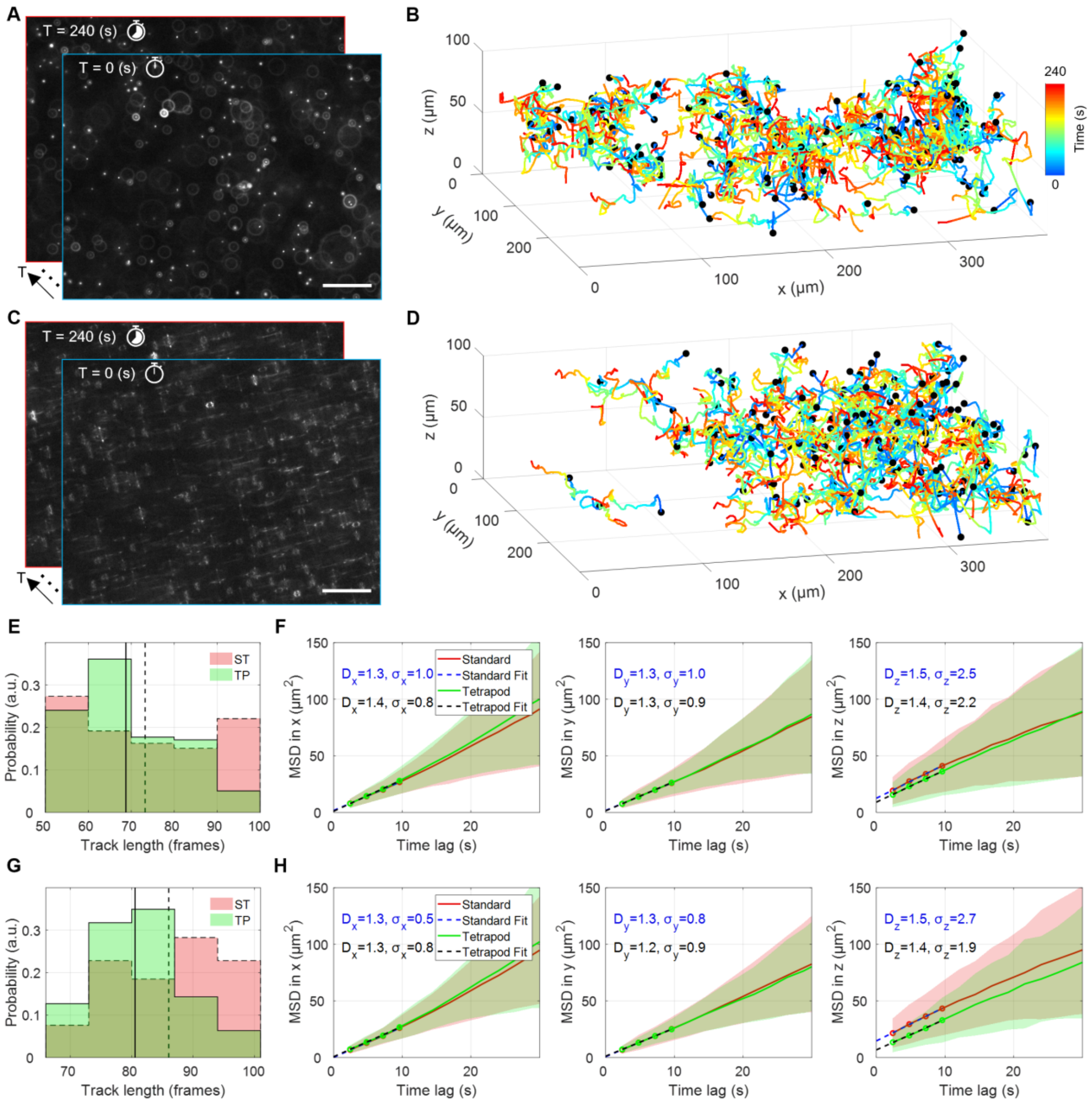

Supplementary Figure 13 - Comparison of 3D tracking at high particle density. **A.** Time lapse of snapshots with the Standard PSF. Scale bar is 50 μm. **B.** Recovered 3D tracks spanning 240 seconds with a 2.4 second resolution. **C.-D.** Time lapse of snapshots with the Tetrapod PSF and the recovered 3D tracks. **E.** Track length distribution in frames (where 100 frames = 240 seconds). Solid and dashed black lines mark the mean track length for the standard/Tetrapod PSFs respectively. **F.** Ensemble mean square displacement (MSD) per axis. Filled area marks 1 standard deviation. D and σ are the per-axis estimated diffusion coefficient and localization precision respectively. **G.-H.** Similar to **E-F** with a higher threshold on track length for MSD calculations. Source data are provided as a Source Data file.

Supplementary references

1. Richardson, W. H. Bayesian-Based Iterative Method of Image Restoration*. *J Opt Soc Am* **62**, 55 (1972).
2. Lucy, L. B. An iterative technique for the rectification of observed distributions. *Astron J* **79**, 745 (1974).
3. Thévenaz, P., Ruttimann, U. E. & Unser, M. A pyramid approach to subpixel registration based on intensity. *IEEE Transactions on Image Processing* **7**, 27–41 (1998).
4. Stringer, C., Wang, T., Michaelos, M. & Pachitariu, M. Cellpose: a generalist algorithm for cellular segmentation. *Nat Methods* **18**, 100–106 (2021).
5. Falk, T. *et al.* U-Net: deep learning for cell counting, detection, and morphometry. *Nat Methods* **16**, 67–70 (2019).
6. Ronneberger, O., Fischer, P. & Brox, T. U-net: Convolutional networks for biomedical image segmentation. *Lecture Notes in Computer Science (including subseries Lecture Notes in Artificial Intelligence and Lecture Notes in Bioinformatics)* **9351**, 234–241 (2015).
7. Ulyanov, D., Vedaldi, A. & Lempitsky, V. Instance Normalization: The Missing Ingredient for Fast Stylization. (2016).
8. Loshchilov, I. & Hutter, F. Decoupled weight decay regularization. *7th International Conference on Learning Representations, ICLR 2019* (2019).
9. Ollion, J., Cochennec, J., Loll, F., Escudé, C. & Boudier, T. TANGO: A generic tool for high-throughput 3D image analysis for studying nuclear organization. *Bioinformatics* **29**, 1840–1841 (2013).
10. Pachitariu, M. & Stringer, C. Cellpose 2.0: how to train your own model. *Nat Methods* **19**, 1634–1641 (2022).
11. Schmidt, U., Weigert, M., Broaddus, C. & Myers, G. Cell detection with star-convex polygons. *Lecture Notes in Computer Science (including subseries Lecture Notes in Artificial Intelligence and Lecture Notes in Bioinformatics)* **11071 LNCS**, 265–273 (2018).
12. Weigert, M., Schmidt, U., Haase, R., Sugawara, K. & Myers, G. Star-convex polyhedra for 3D object detection and segmentation in microscopy. *Proceedings - 2020 IEEE Winter Conference on Applications of Computer Vision, WACV 2020* 3655–3662 (2020) doi:10.1109/WACV45572.2020.9093435.
13. Michalet, X. Mean Square Displacement Analysis of Single-Particle Trajectories with Localization Error. *Biophys J* **100**, 252a (2011).
14. Nehme, E. *et al.* DeepSTORM3D: dense 3D localization microscopy and PSF design by deep learning. *Nat Methods* **17**, 734–740 (2020).
