# Supplementary material for: Depth-enhanced high-throughput microscopy by compact PSF engineering
Source: Nat Commun. 2024 Jun 7;15:4861. doi: 10.1038/s41467-024-48502-y (PMC11161645; doi:10.1038/s41467-024-48502-y)
Supplement: Supplementary file 2 — Description of Additional Supplementary Files [file 41467_2024_48502_MOESM2_ESM.pdf]

Title: Supplementary Movie 1 (Objecfive.avi)
Description: Visualization of the phase-mask mounting onto the objective. The video shows the modality presented in Fig. 1B.

Title: Supplementary Movie 2 (EDOF_Spheroid.mp4)
Description: Alternating overlapped images of a spheroid, with and without EDOF. In the standard PSF display are the three images presented in Fig. 3.C (bottom row), in the EDOF display are three replicas of the top row image of Fig 3.C.

Title: Supplementary Movie 3 (SegmentafionsOverlap.mov)
Description: Axial scan with 4 µm steps in z of the segmentations in Fig. 5.B,E (left, middle) and their binary overlap in Fig.5.H (right). Slice height is shown in µm at the bottom left. Scale bar is 100 µm.

Title: Supplementary Movie 4 (RenderingS4D.mov)
Description: Rotating 3D rendering of the results presented in Fig. 5.C,F,I.

Title: Supplementary Movie 5 (Tracking3D.mov)
Description: Time lapse of the 3D tracks presented in Fig. 6.B accumulated over time. Scale bar is 50 µm.
