# Supplementary material for: Depth-enhanced high-throughput microscopy by compact PSF engineering
Source: Nat Commun. 2024 Jun 7;15:4861. doi: 10.1038/s41467-024-48502-y (PMC11161645; doi:10.1038/s41467-024-48502-y)
Supplement: Supplementary file 10 — Source Data [file 41467_2024_48502_MOESM10_ESM.zip › Main - Figure 2/Fig2.pptx]

## Slide 1
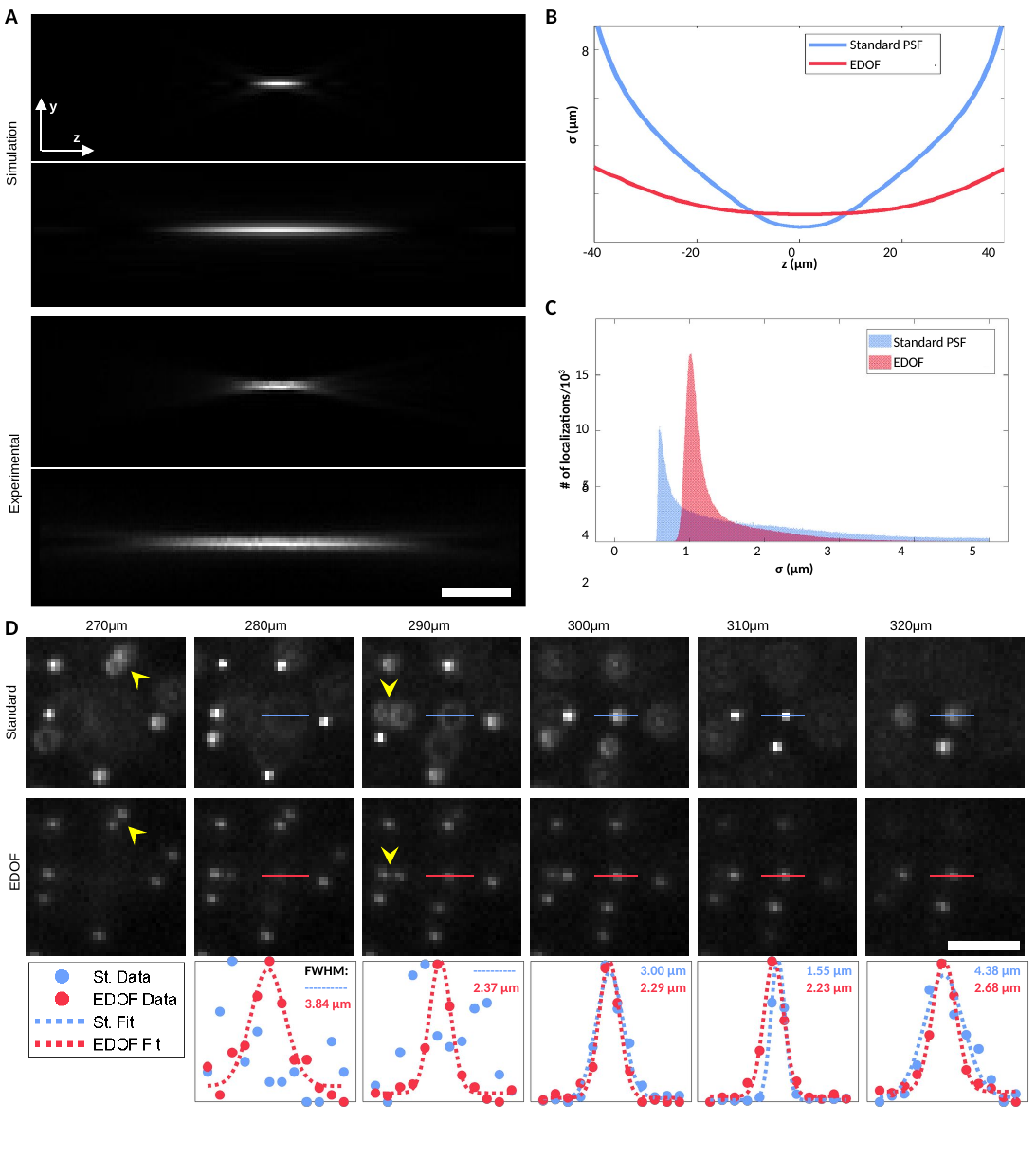

A
B
Standard PSF
8
 6
4
2
EDOF
y
σ (μm)
Simulation
z
-40 -20 0 20 40
z (μm)
C
Standard PSF
EDOF
15
10
# of localizations/103
Experimental
5
0 1 2 3 4 5
σ (μm)
D
270μm 280μm 290μm 300μm 310μm 320μm
Standard
EDOF
FWHM:
----------
3.84 μm
----------
2.37 μm
3.00 μm
2.29 μm
1.55 μm
2.23 μm
4.38 μm
2.68 μm
