# Supplementary material for: Depth-enhanced high-throughput microscopy by compact PSF engineering
Source: Nat Commun. 2024 Jun 7;15:4861. doi: 10.1038/s41467-024-48502-y (PMC11161645; doi:10.1038/s41467-024-48502-y)
Supplement: Supplementary file 10 — Source Data [file 41467_2024_48502_MOESM10_ESM.zip › Main - Figure 5/CellSnap_example_results.pptx]

## Slide 1
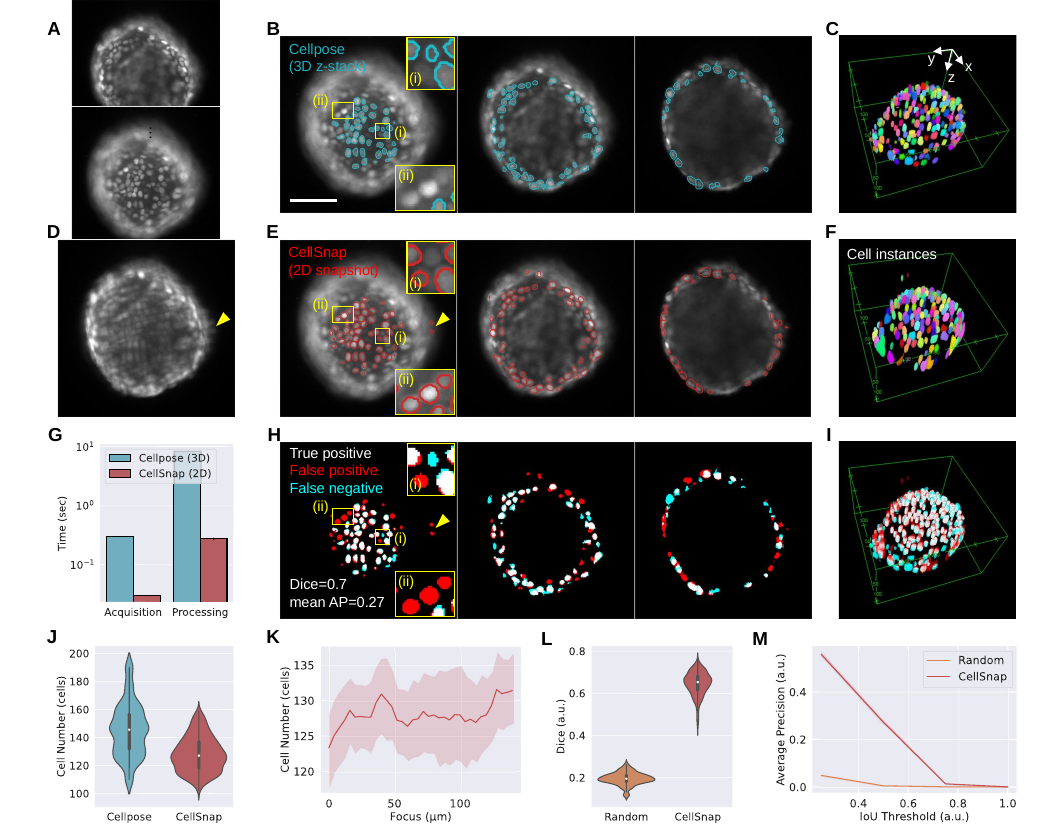

…
A
B
C
Cellpose
(3D z-stack)
y
x
z
(i)
(ii)
(i)
(ii)
D
E
F
CellSnap
(2D snapshot)
Cell instances
(i)
(ii)
(i)
(ii)
G
H
I
True positive
False positive
False negative
(i)
(ii)
(i)
(ii)
Dice=0.7
mean AP=0.27
J
K
L
M
