# Supplementary material for: Depth-enhanced high-throughput microscopy by compact PSF engineering
Source: Nat Commun. 2024 Jun 7;15:4861. doi: 10.1038/s41467-024-48502-y (PMC11161645; doi:10.1038/s41467-024-48502-y)
Supplement: Supplementary file 10 — Source Data [file 41467_2024_48502_MOESM10_ESM.zip › Main - Figure 4/CellSnap_architecture_and_concept.pptx]

## Slide 1
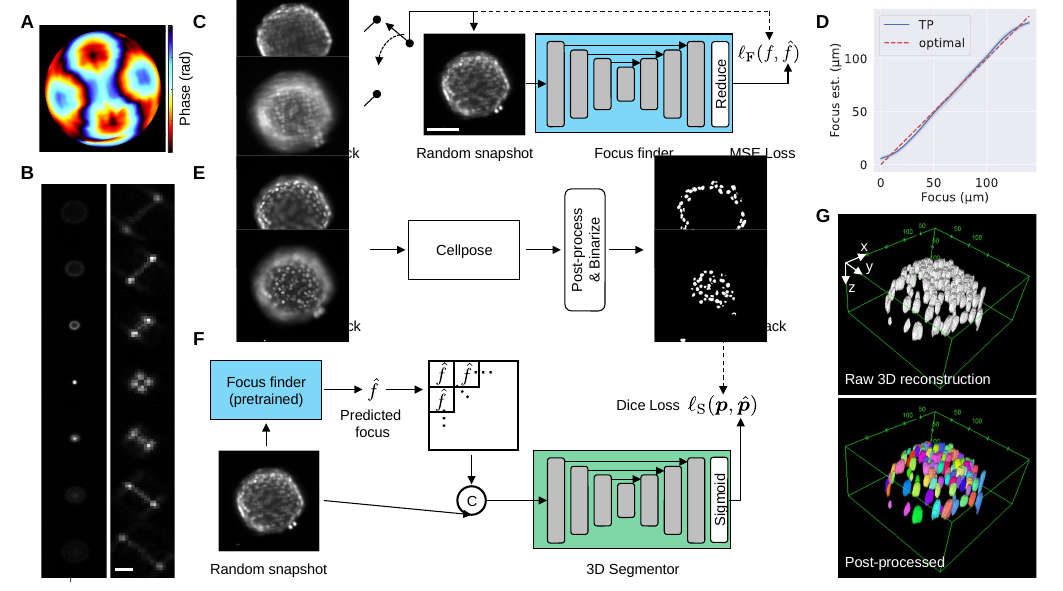

…
Tetrapod z-stack
D
A
C
Reduce
Focus finder
Phase (rad)
Random snapshot
MSE Loss
…
Standard z-stack
…
Segmented z-stack
E
B
G
x
y
z
Raw 3D reconstruction
Cellpose
Post-process & Binarize
F
Focus finder
(pretrained)
…
Dice Loss
…
Post-processed
Predicted
focus
…
C
Sigmoid
Random snapshot
3D Segmentor
