# Supplementary figures and images for: Depth-enhanced high-throughput microscopy by compact PSF engineering
Source: Nat Commun. 2024 Jun 7;15:4861. doi: 10.1038/s41467-024-48502-y (PMC11161645; doi:10.1038/s41467-024-48502-y)

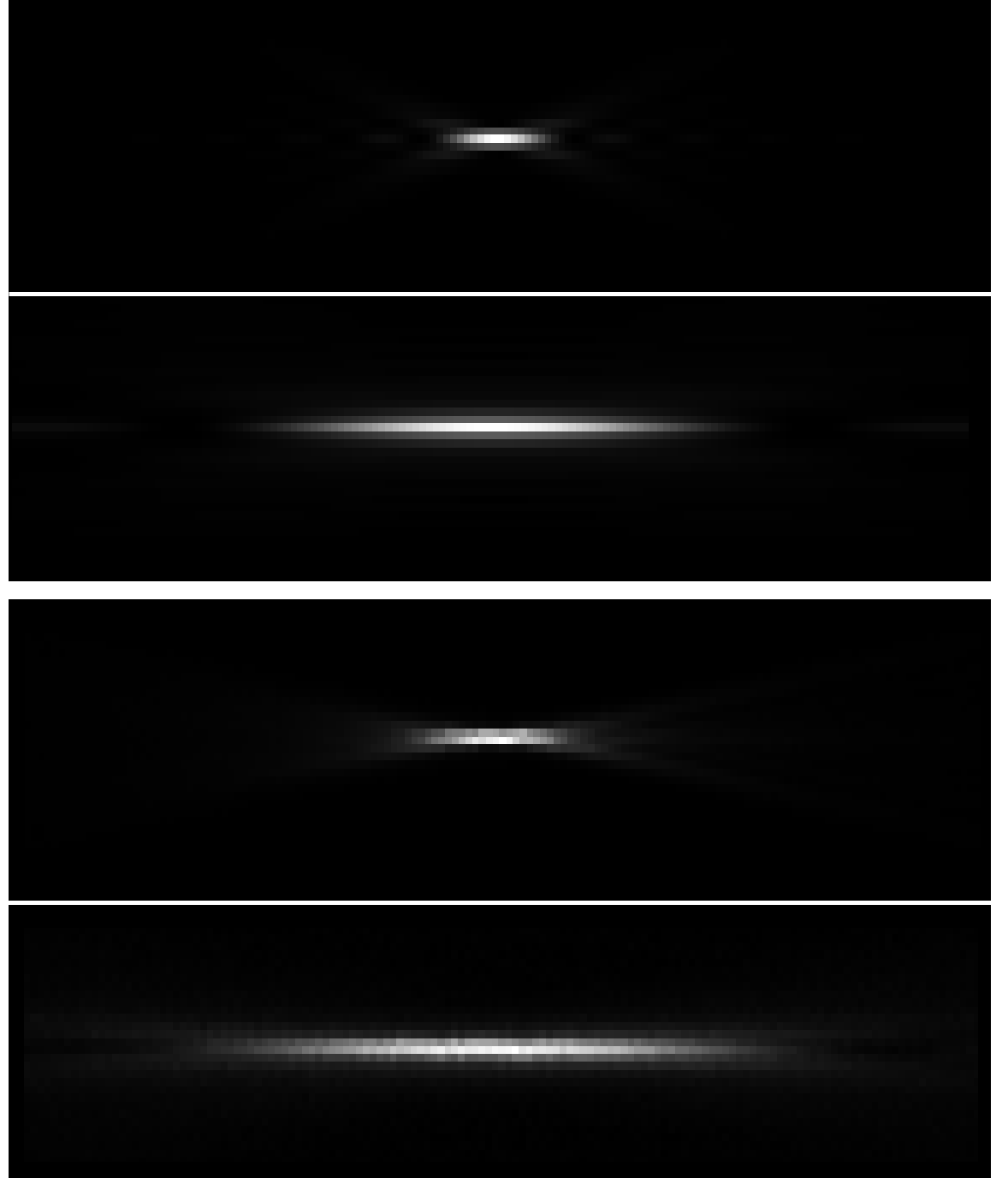

Supplement: Supplementary file 10 — Source Data [file 41467_2024_48502_MOESM10_ESM.zip › Main - Figure 2/SeparatePanels/2A.png]

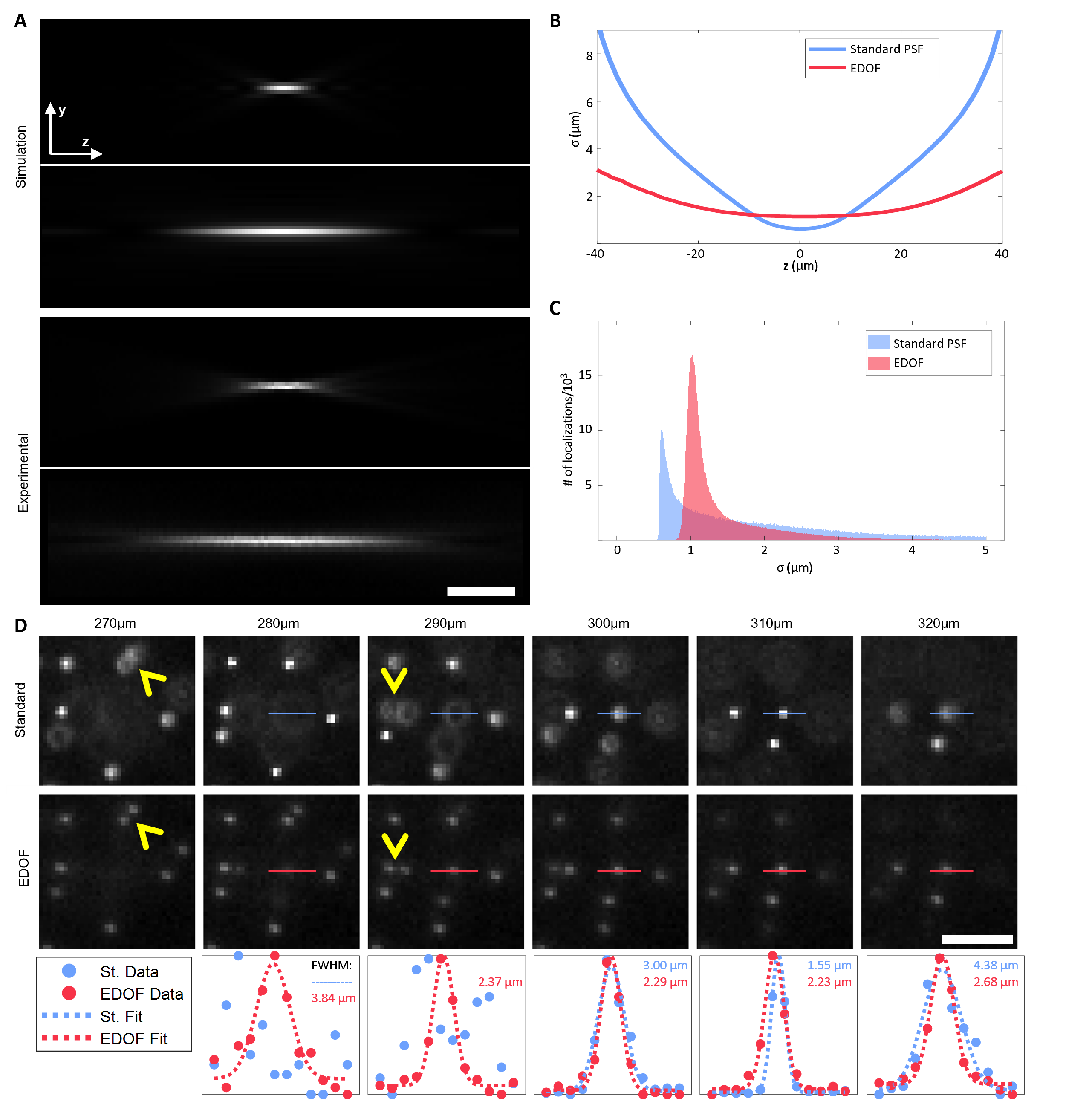

Supplement: Supplementary file 10 — Source Data [file 41467_2024_48502_MOESM10_ESM.zip › Main - Figure 2/SeparatePanels/Figure 2_PNG.png]

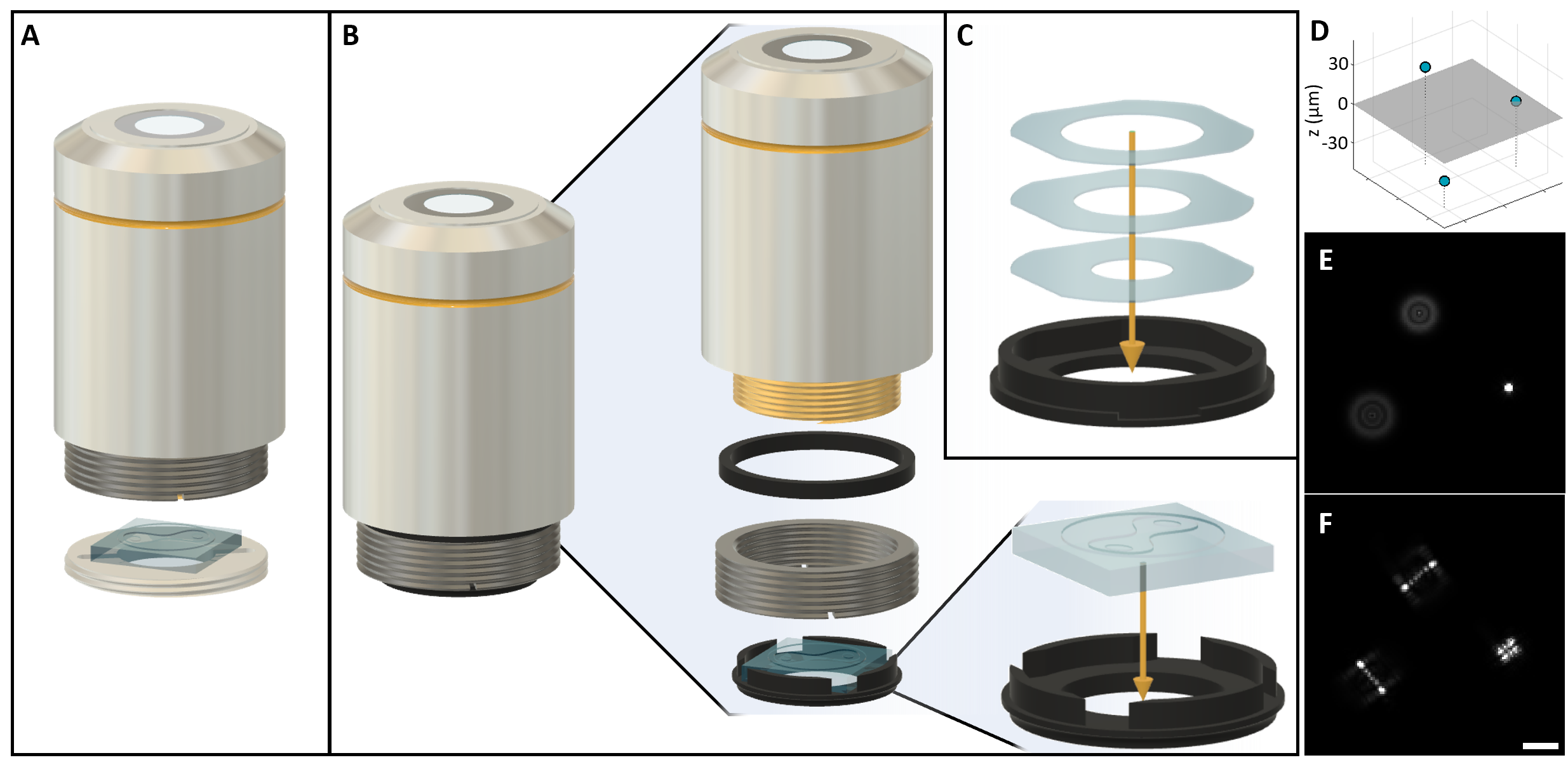

Supplement: Supplementary file 10 — Source Data [file 41467_2024_48502_MOESM10_ESM.zip › Main - Figure 1/Figure 1_PPT.png]

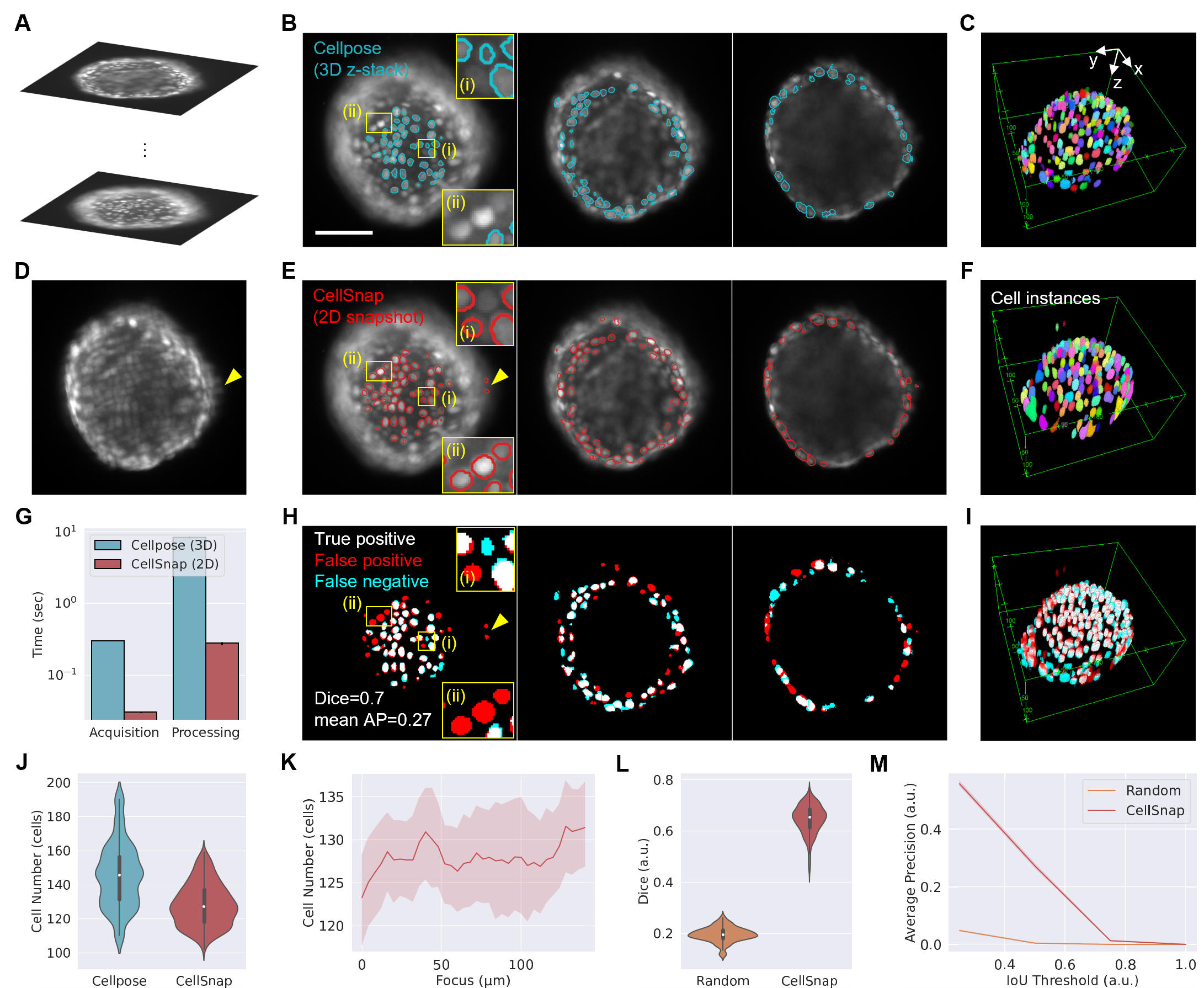

Supplement: Supplementary file 10 — Source Data [file 41467_2024_48502_MOESM10_ESM.zip › Main - Figure 5/CellSnap_example_results.png]

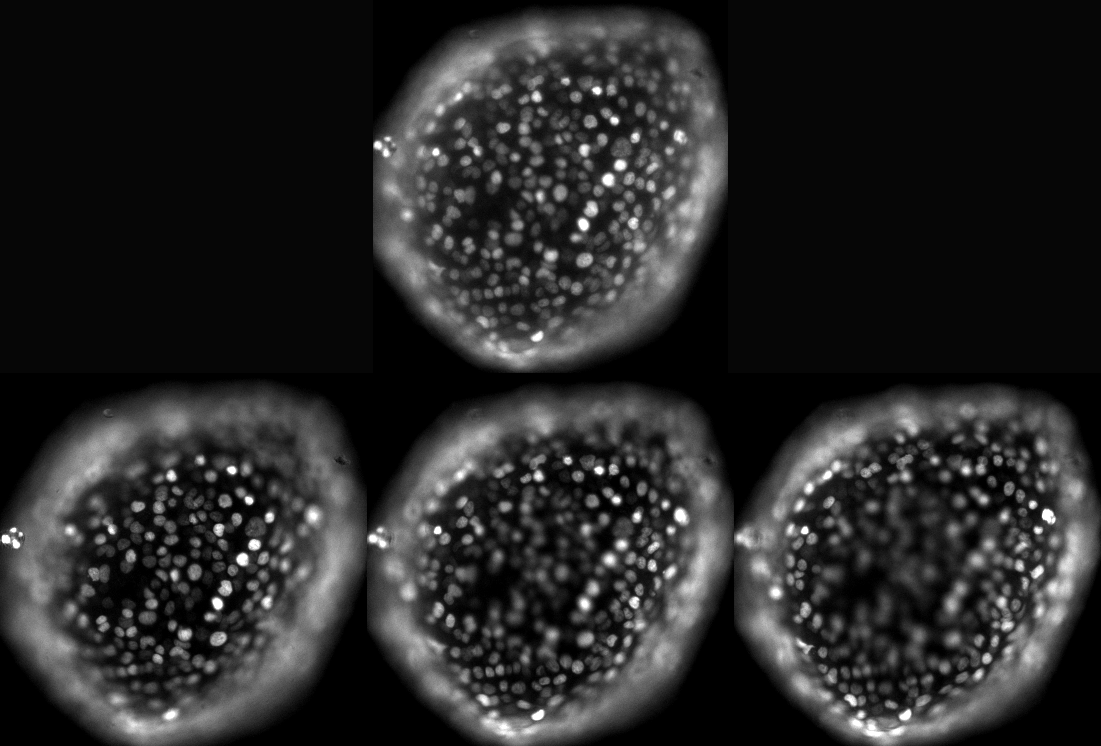

Supplement: Supplementary file 10 — Source Data [file 41467_2024_48502_MOESM10_ESM.zip › Main - Figure 3/SeparatePanels/3C_Large.png]

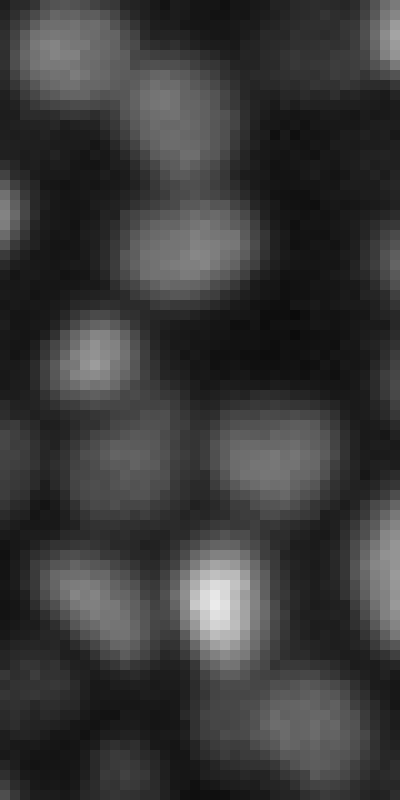

Supplement: Supplementary file 10 — Source Data [file 41467_2024_48502_MOESM10_ESM.zip › Main - Figure 3/SeparatePanels/3C_zoom_V_EDOF.png]

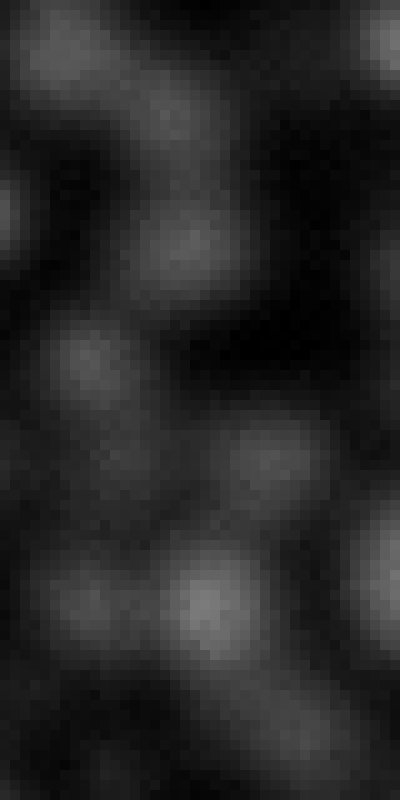

Supplement: Supplementary file 10 — Source Data [file 41467_2024_48502_MOESM10_ESM.zip › Main - Figure 3/SeparatePanels/3C_zoom_V_ST.png]

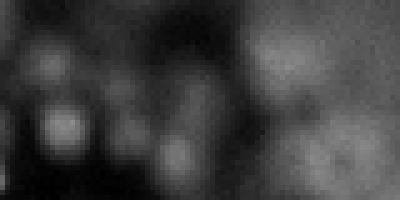

Supplement: Supplementary file 10 — Source Data [file 41467_2024_48502_MOESM10_ESM.zip › Main - Figure 3/SeparatePanels/3C_zoom_H_ST.png]

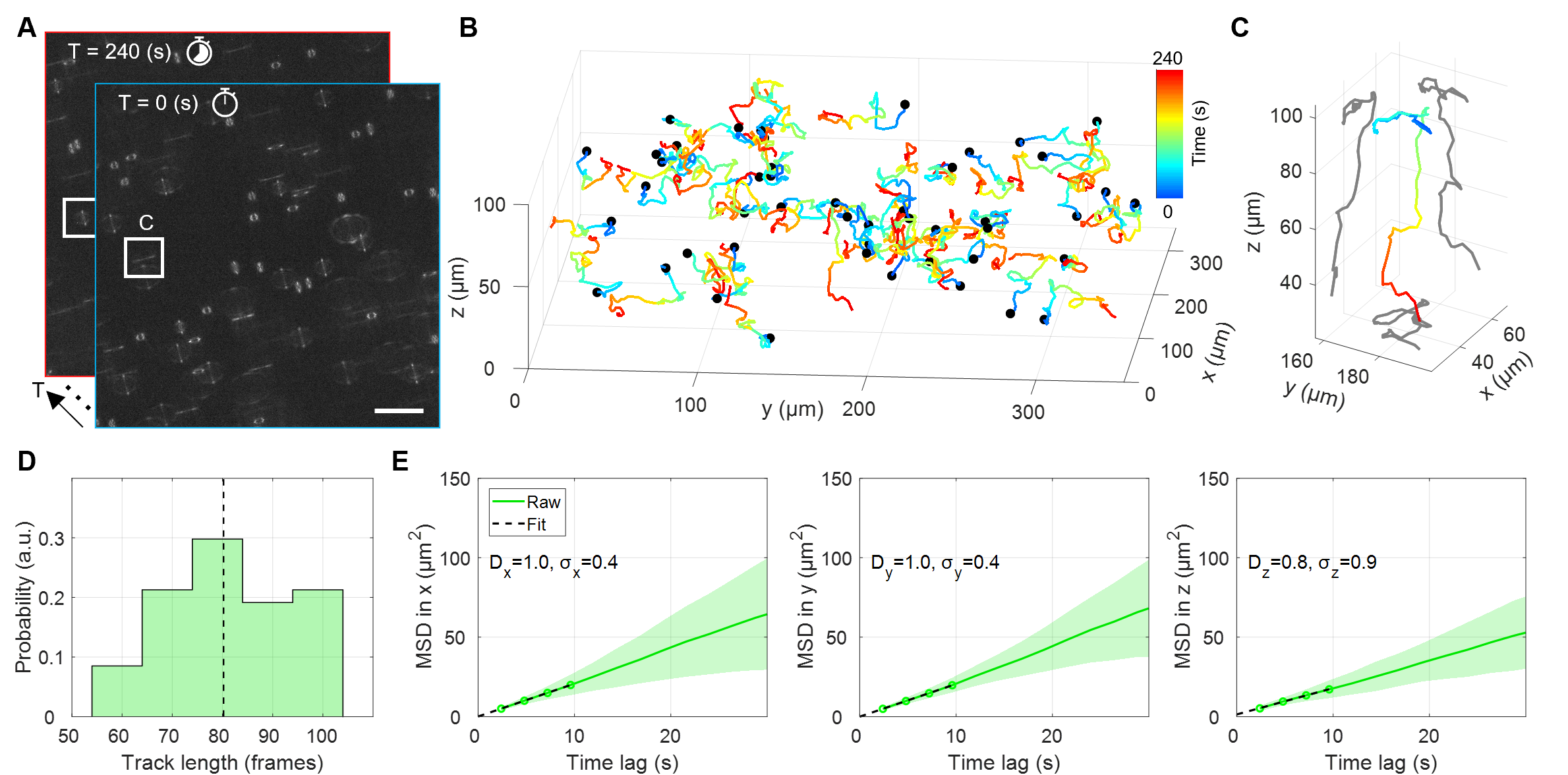

Supplement: Supplementary file 10 — Source Data [file 41467_2024_48502_MOESM10_ESM.zip › Main - Figure 6/NTA_and_3D_tracking.png]

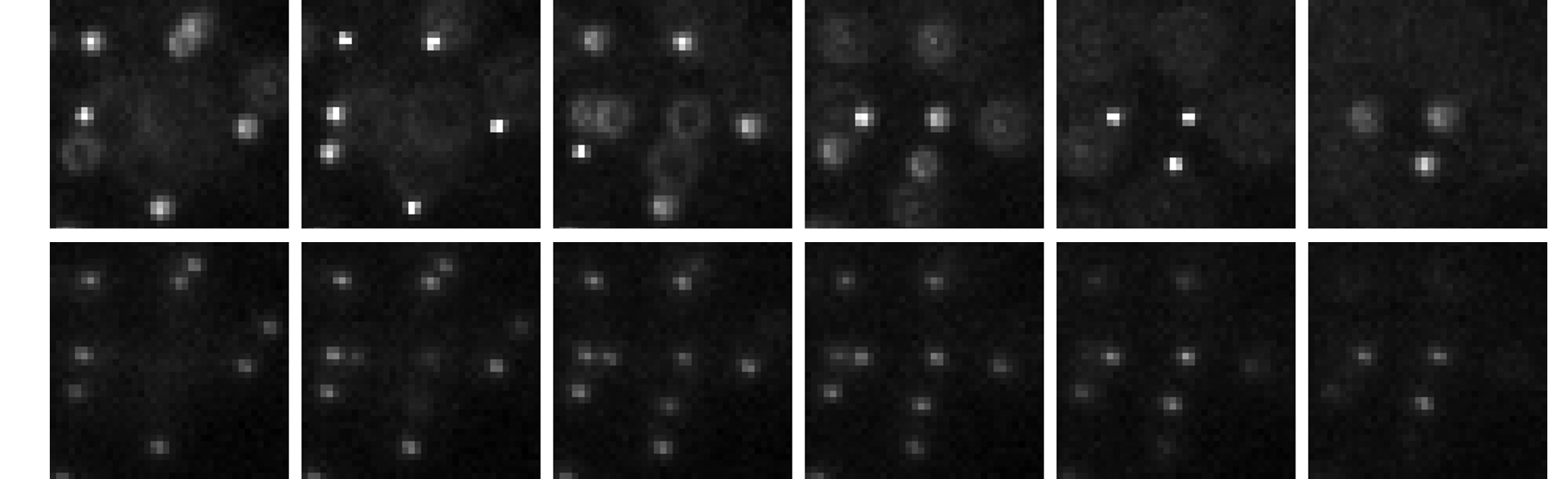

Supplement: Supplementary file 10 — Source Data [file 41467_2024_48502_MOESM10_ESM.zip › Main - Figure 2/SeparatePanels/2D.png]

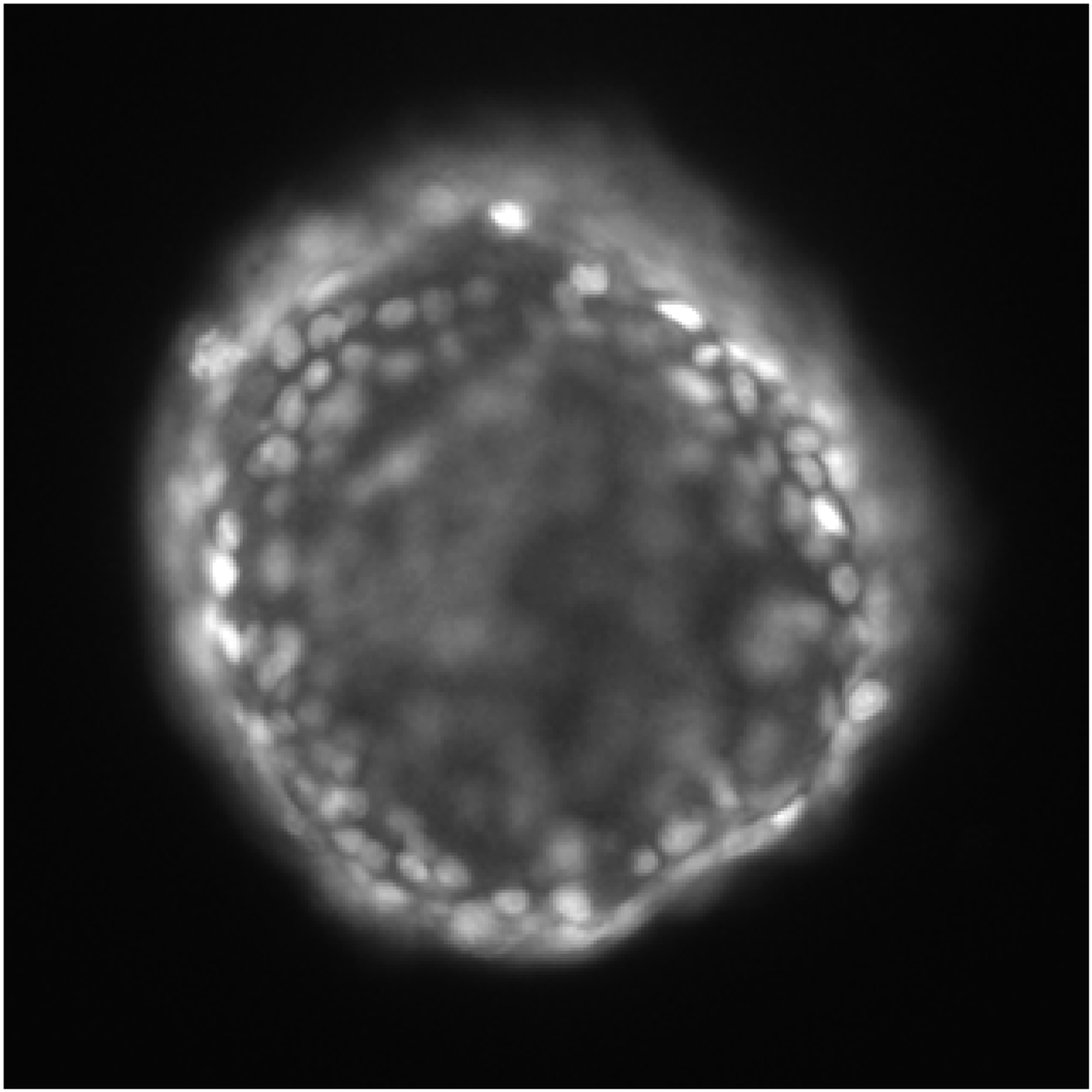

Supplement: Supplementary file 10 — Source Data [file 41467_2024_48502_MOESM10_ESM.zip › Main - Figure 5/SeparatePanels/Figure5_A_top.png]

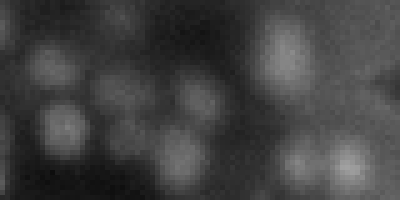

Supplement: Supplementary file 10 — Source Data [file 41467_2024_48502_MOESM10_ESM.zip › Main - Figure 3/SeparatePanels/3C_zoom_H_EDOF.png]

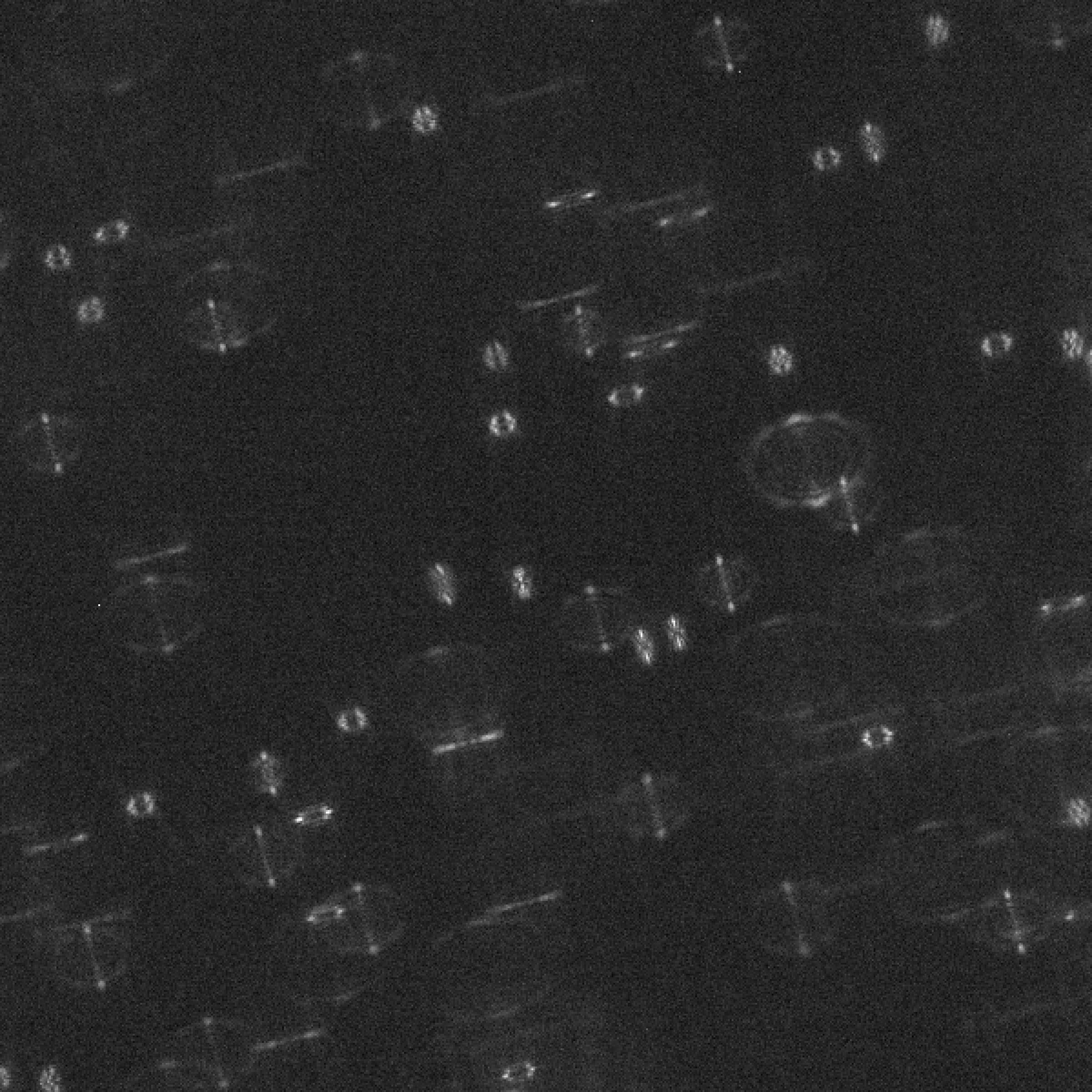

Supplement: Supplementary file 10 — Source Data [file 41467_2024_48502_MOESM10_ESM.zip › Main - Figure 6/SeparatePanels/Figure6_A_frame1.png]

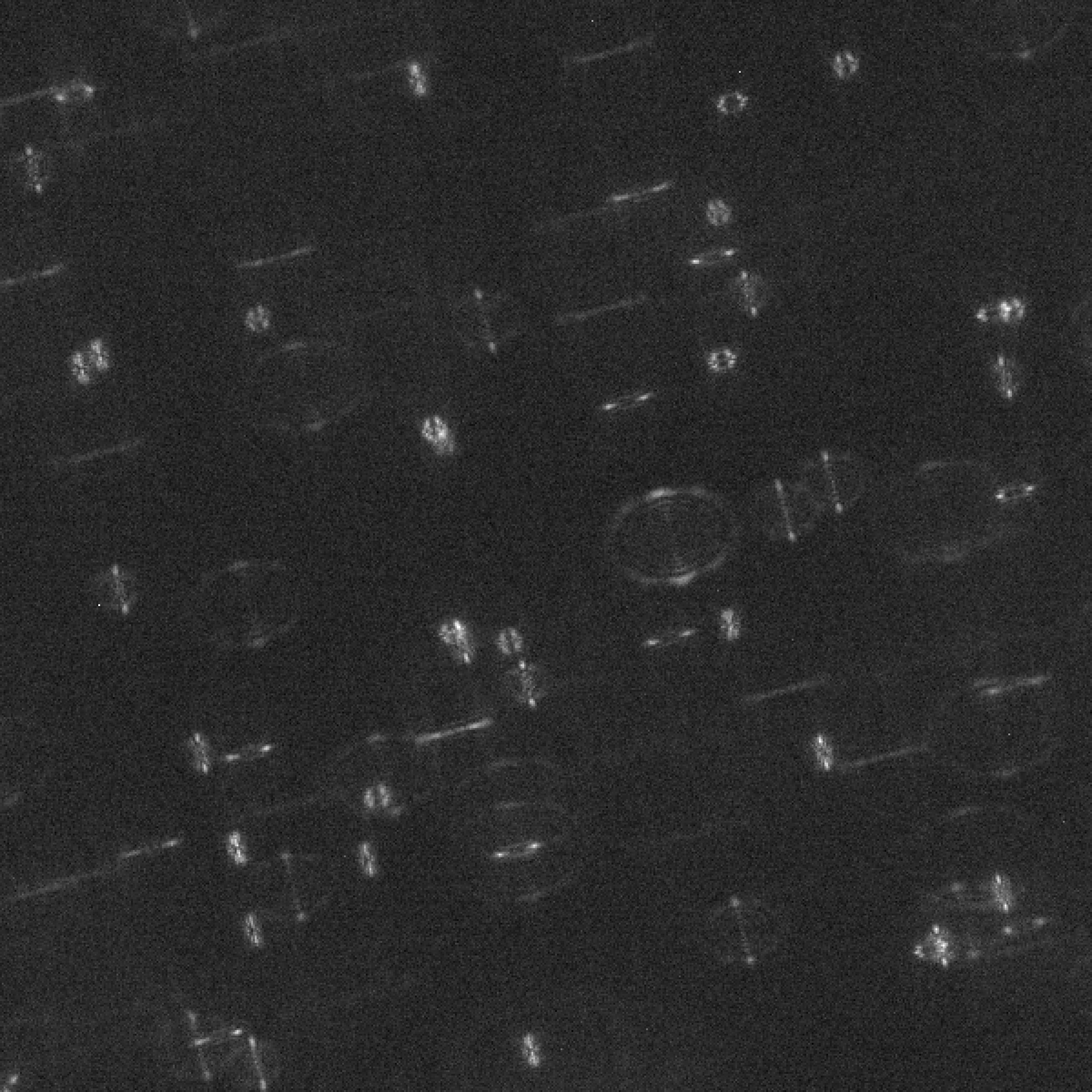

Supplement: Supplementary file 10 — Source Data [file 41467_2024_48502_MOESM10_ESM.zip › Main - Figure 6/SeparatePanels/Figure6_A_frame100.png]

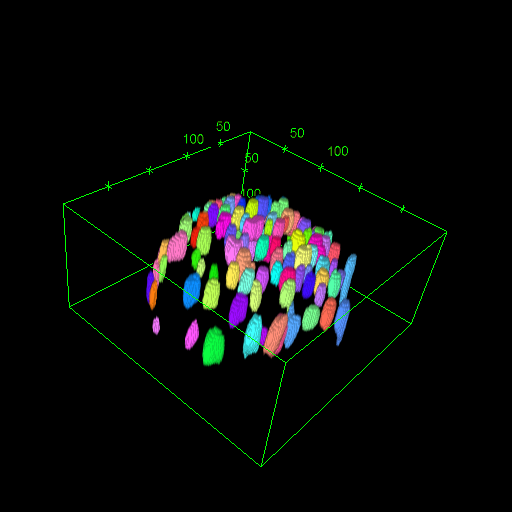

Supplement: Supplementary file 10 — Source Data [file 41467_2024_48502_MOESM10_ESM.zip › Main - Figure 4/SeparatePanels/Figure4_G_post_process_3d.png]

## Slide 1
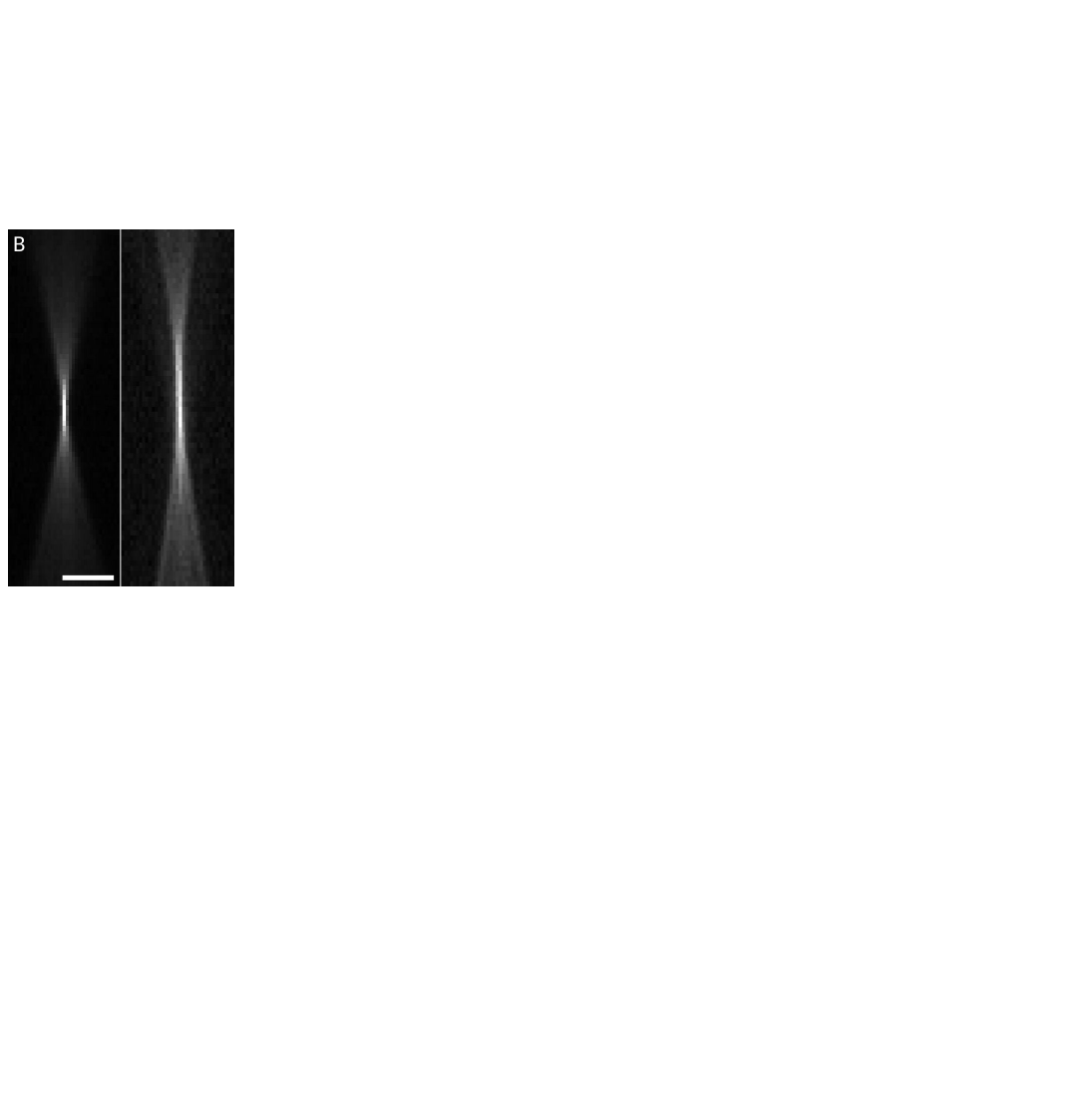

B
(i)
Standard

Supplement: Supplementary file 10 — Source Data [file 41467_2024_48502_MOESM10_ESM.zip › Main - Figure 3/SeparatePanels/Fig3B.pptx]

## Slide 1
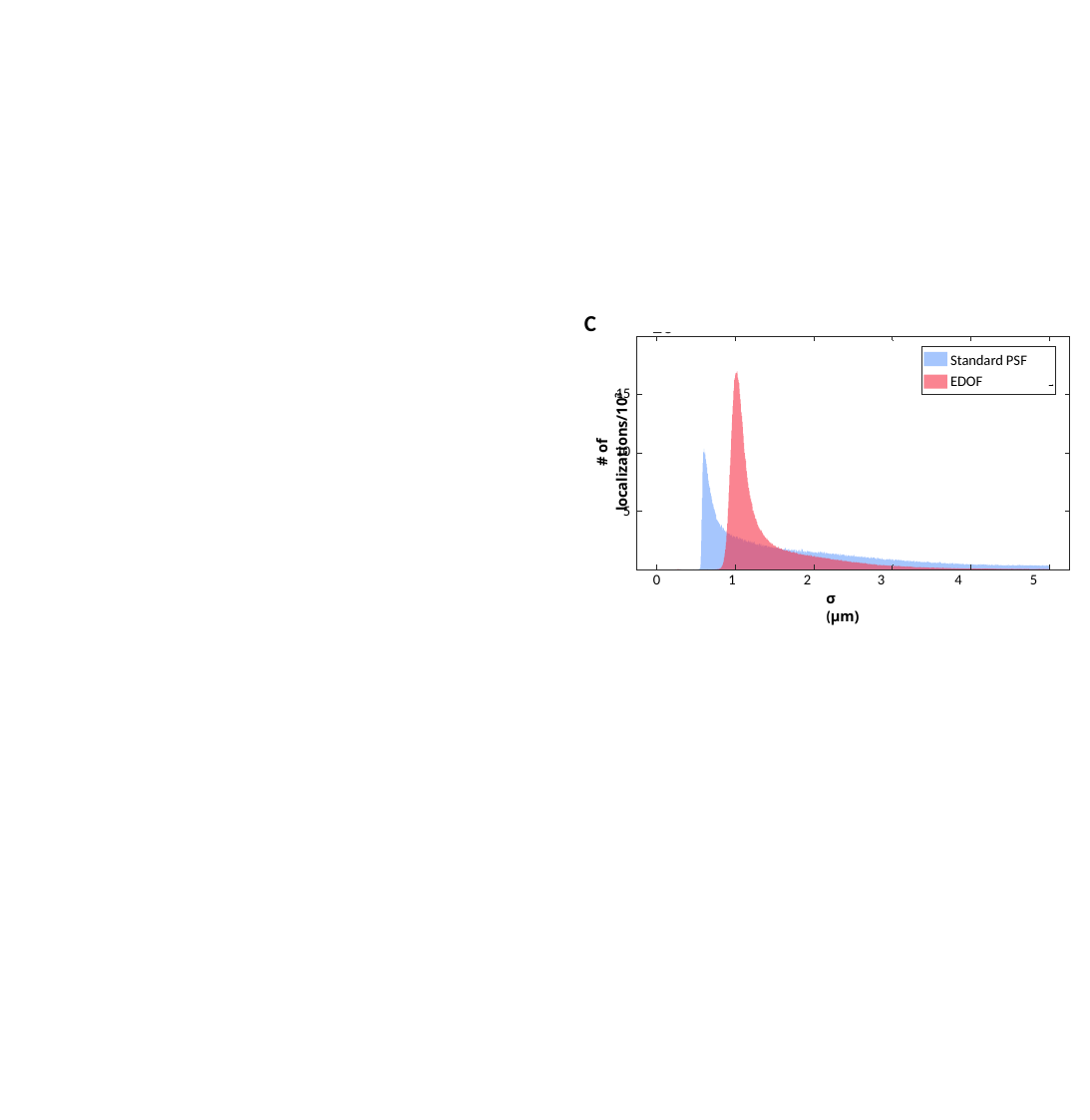

C
Standard PSF
EDOF
15
10
# of localizations/103
5
0 1 2 3 4 5
σ (μm)

Supplement: Supplementary file 10 — Source Data [file 41467_2024_48502_MOESM10_ESM.zip › Main - Figure 2/SeparatePanels/Fig 2C.pptx]

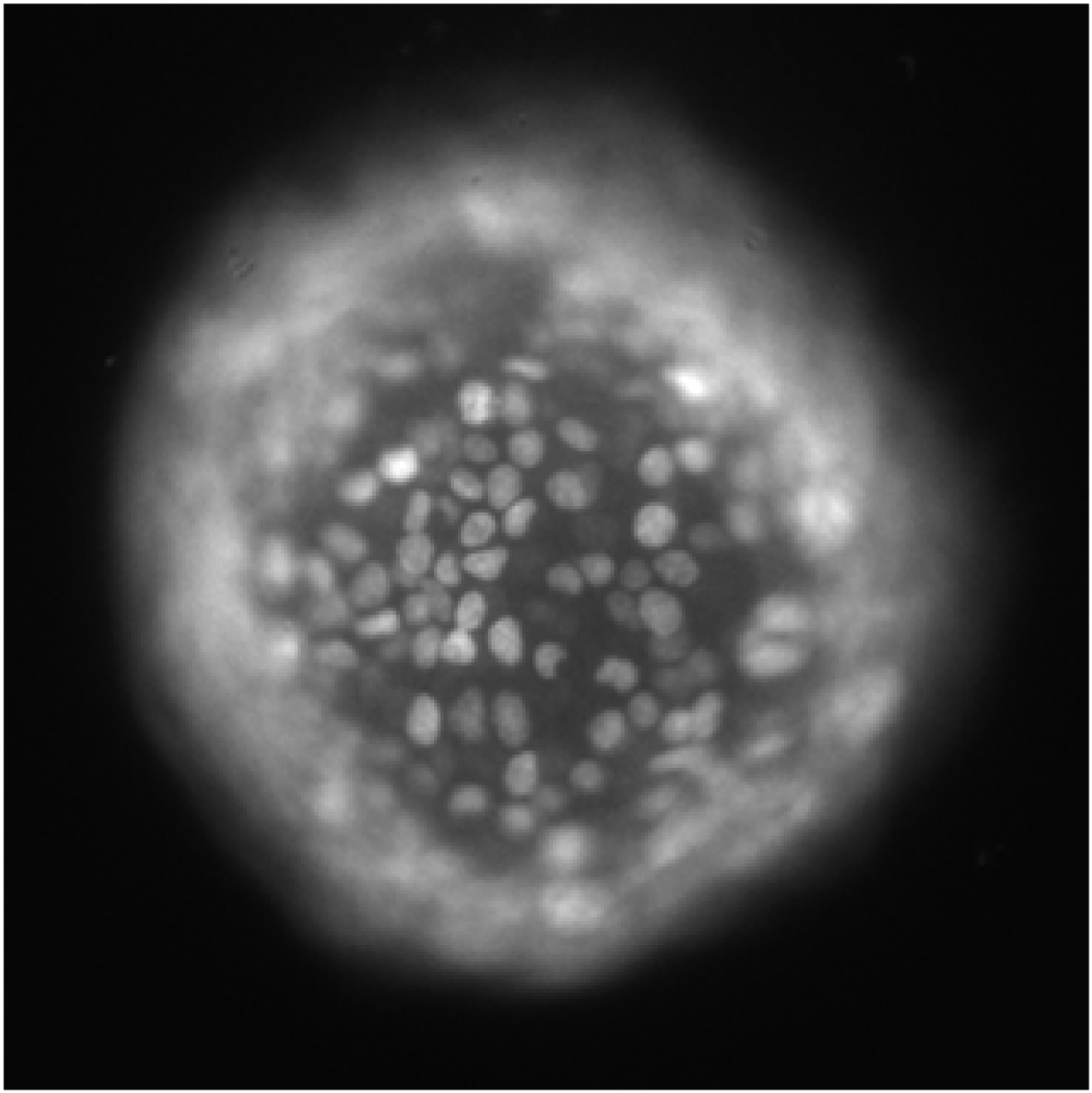

Supplement: Supplementary file 10 — Source Data [file 41467_2024_48502_MOESM10_ESM.zip › Main - Figure 5/SeparatePanels/Figure5_A_bottom.png]

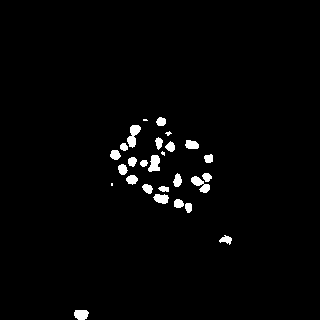

Supplement: Supplementary file 10 — Source Data [file 41467_2024_48502_MOESM10_ESM.zip › Main - Figure 4/SeparatePanels/Figure4_E_Segmented_low.png]

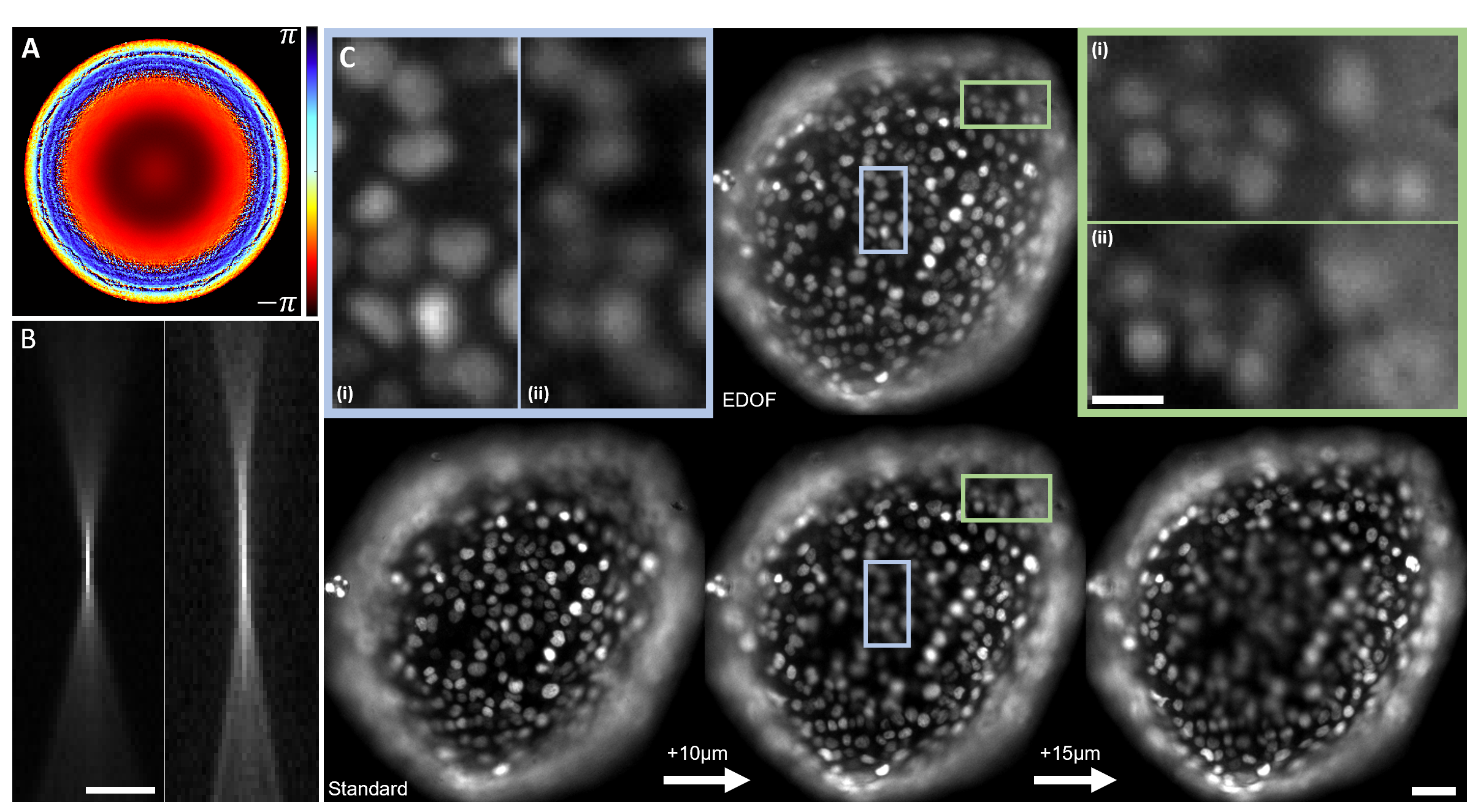

Supplement: Supplementary file 10 — Source Data [file 41467_2024_48502_MOESM10_ESM.zip › Main - Figure 3/Figure 3_PNG.png]

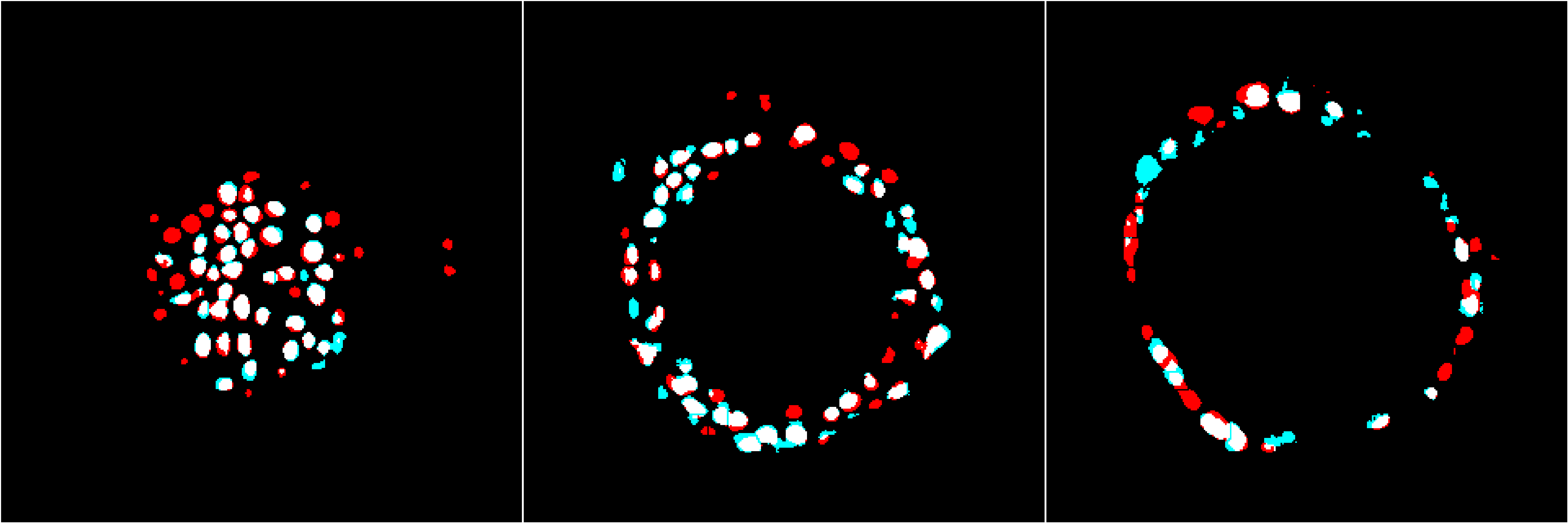

Supplement: Supplementary file 10 — Source Data [file 41467_2024_48502_MOESM10_ESM.zip › Main - Figure 5/SeparatePanels/Figure5_H.png]

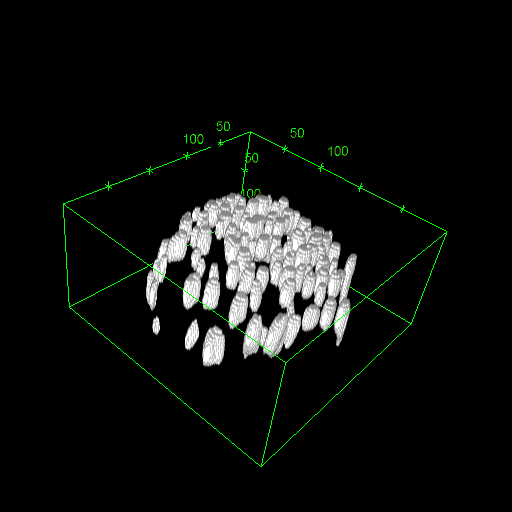

Supplement: Supplementary file 10 — Source Data [file 41467_2024_48502_MOESM10_ESM.zip › Main - Figure 4/SeparatePanels/Figure4_G_raw_3d.png]

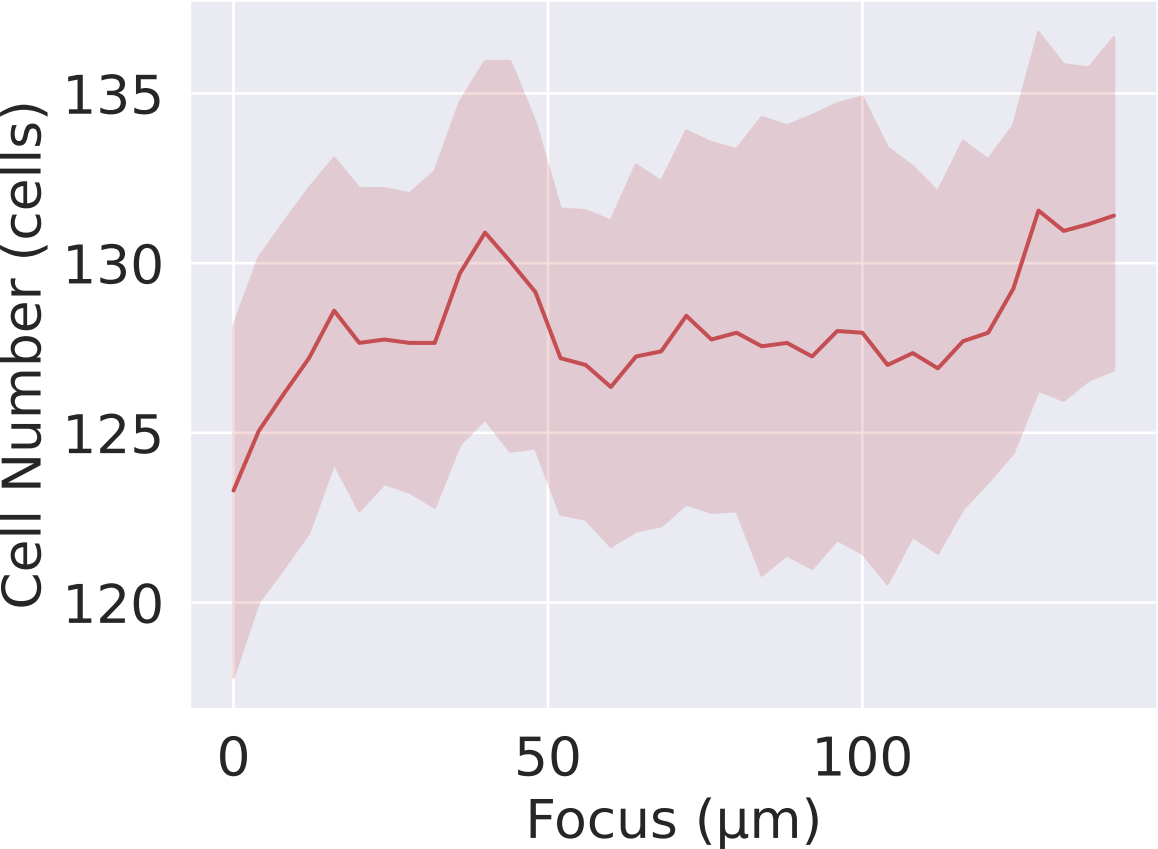

Supplement: Supplementary file 10 — Source Data [file 41467_2024_48502_MOESM10_ESM.zip › Main - Figure 5/SeparatePanels/Figure5_K.pdf]

## Slide 1
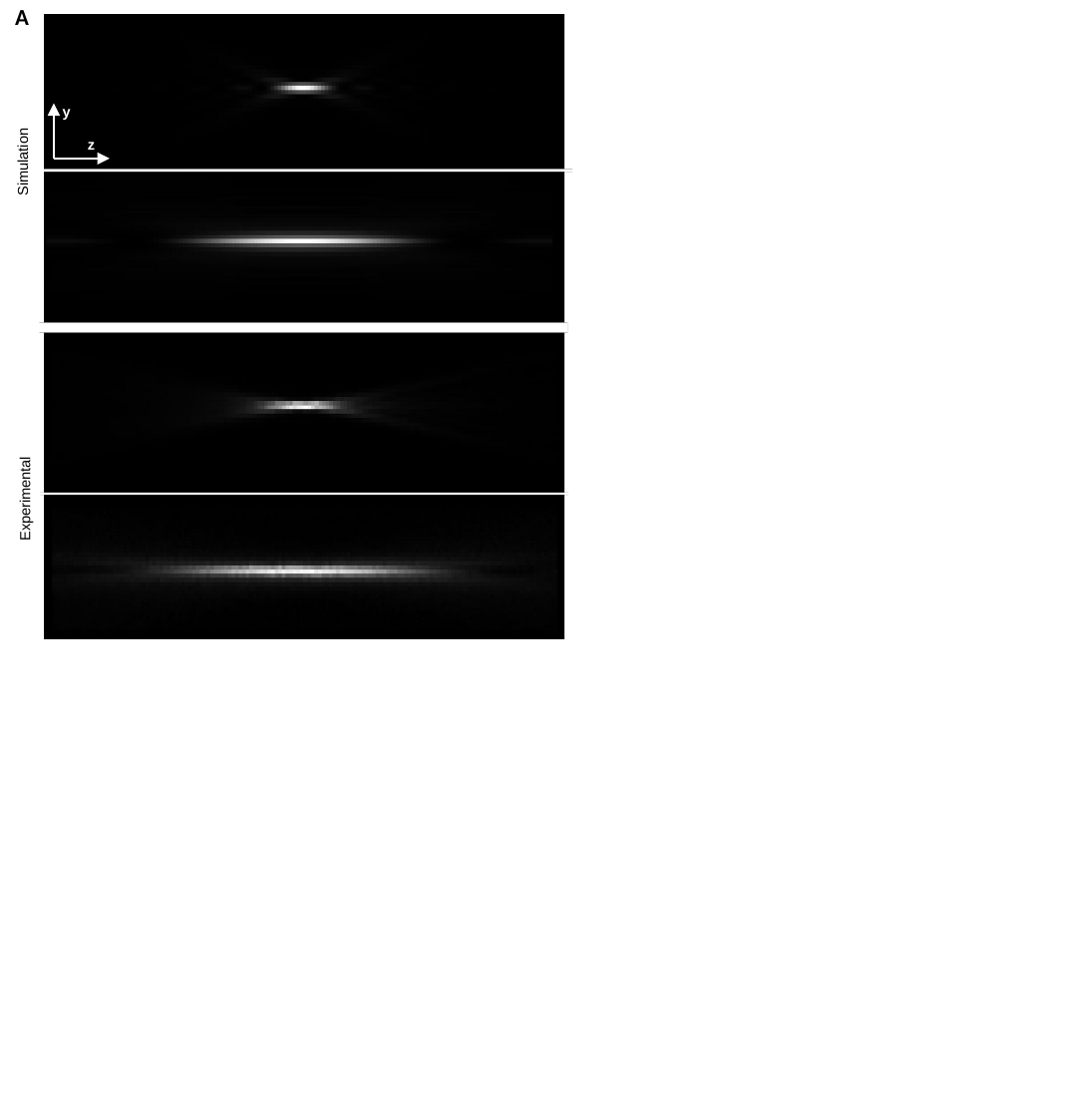

A
y
Simulation
z
Experimental

Supplement: Supplementary file 10 — Source Data [file 41467_2024_48502_MOESM10_ESM.zip › Main - Figure 2/SeparatePanels/Fig 2A.pptx]

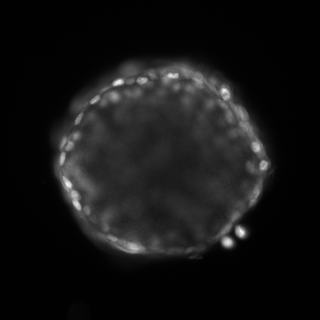

Supplement: Supplementary file 10 — Source Data [file 41467_2024_48502_MOESM10_ESM.zip › Main - Figure 4/SeparatePanels/Figure4_E_Standard_high.png]

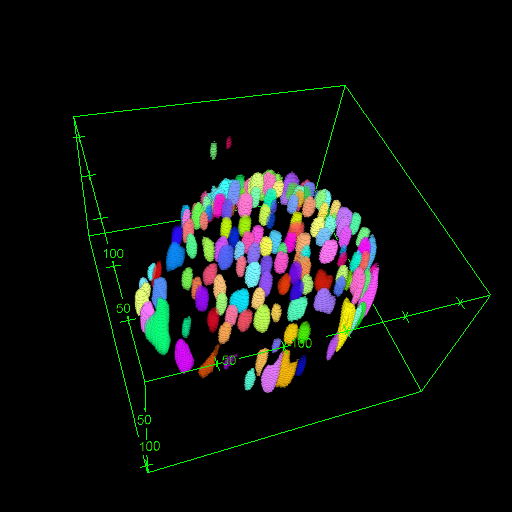

Supplement: Supplementary file 10 — Source Data [file 41467_2024_48502_MOESM10_ESM.zip › Main - Figure 5/SeparatePanels/Figure5_F.png]

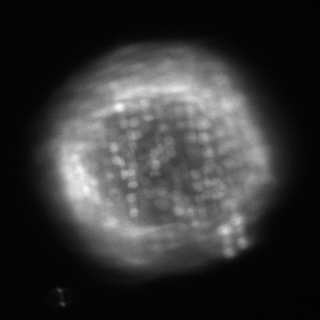

Supplement: Supplementary file 10 — Source Data [file 41467_2024_48502_MOESM10_ESM.zip › Main - Figure 4/SeparatePanels/Figure4_C_Tetrapod_low.png]

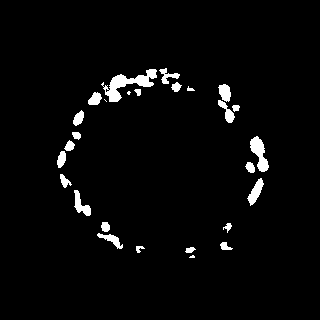

Supplement: Supplementary file 10 — Source Data [file 41467_2024_48502_MOESM10_ESM.zip › Main - Figure 4/SeparatePanels/Figure4_E_Segmented_high.png]

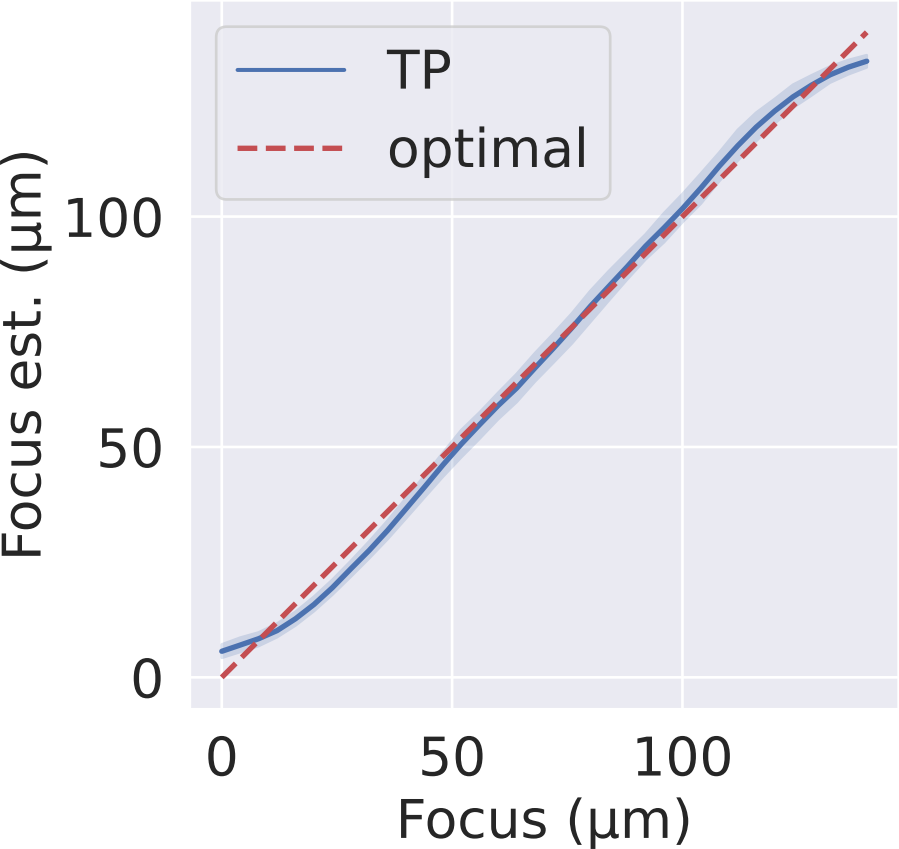

Supplement: Supplementary file 10 — Source Data [file 41467_2024_48502_MOESM10_ESM.zip › Main - Figure 4/SeparatePanels/Figure4_D.pdf]

## Slide 1
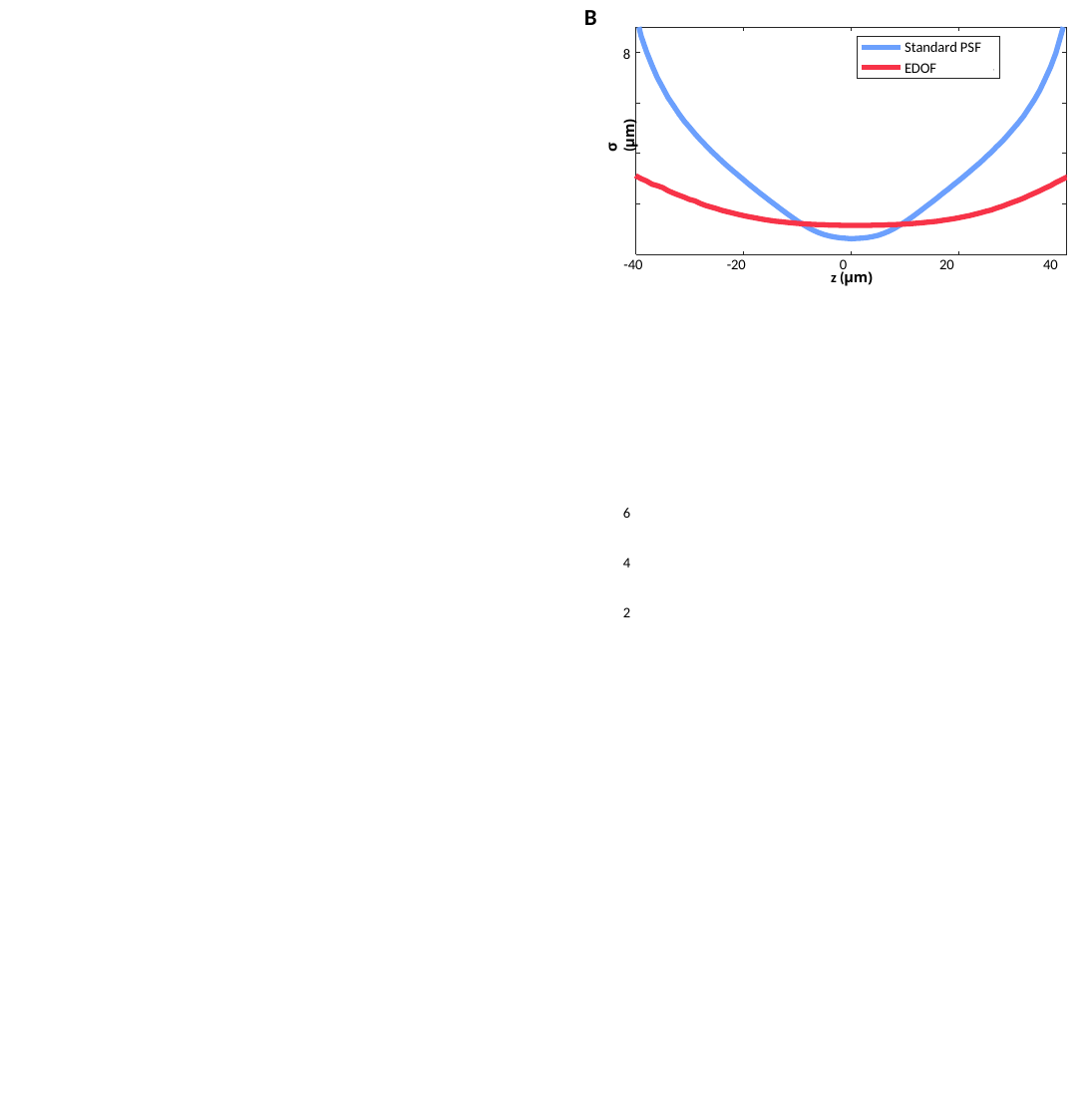

B
Standard PSF
8
 6
4
2
EDOF
σ (μm)
-40 -20 0 20 40
z (μm)

Supplement: Supplementary file 10 — Source Data [file 41467_2024_48502_MOESM10_ESM.zip › Main - Figure 2/SeparatePanels/Fig 2B.pptx]

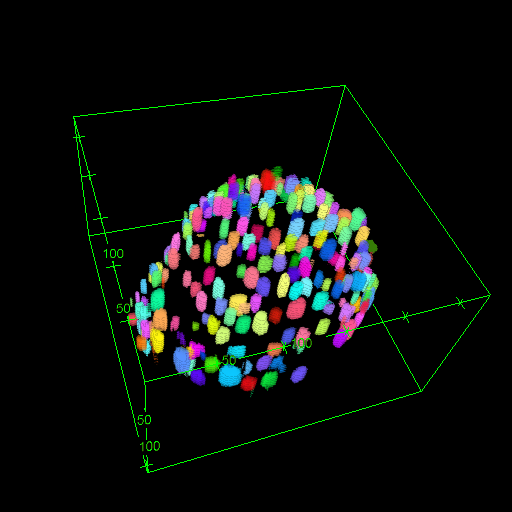

Supplement: Supplementary file 10 — Source Data [file 41467_2024_48502_MOESM10_ESM.zip › Main - Figure 5/SeparatePanels/Figure5_C.png]

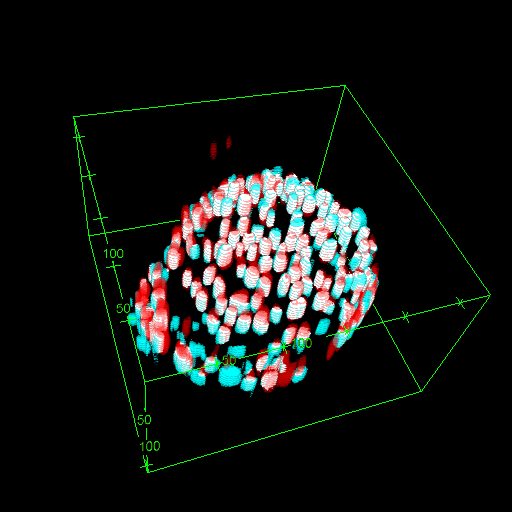

Supplement: Supplementary file 10 — Source Data [file 41467_2024_48502_MOESM10_ESM.zip › Main - Figure 5/SeparatePanels/Figure5_I.png]

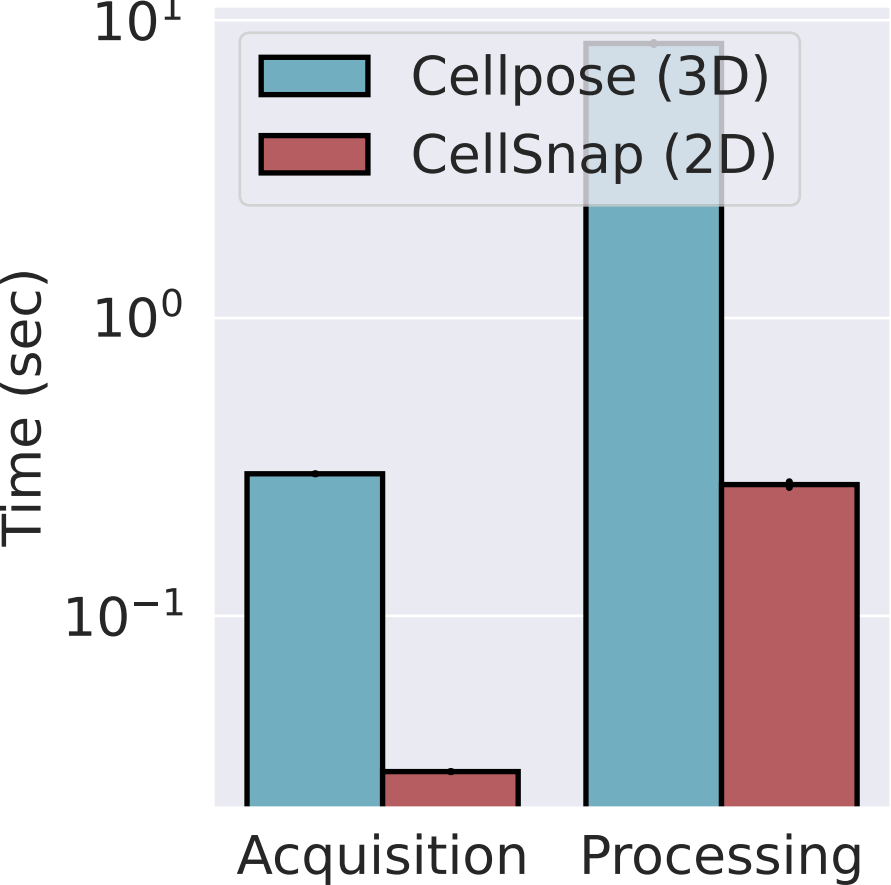

Supplement: Supplementary file 10 — Source Data [file 41467_2024_48502_MOESM10_ESM.zip › Main - Figure 5/SeparatePanels/Figure5_G.pdf]

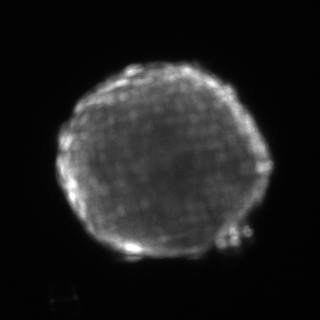

Supplement: Supplementary file 10 — Source Data [file 41467_2024_48502_MOESM10_ESM.zip › Main - Figure 4/SeparatePanels/Figure4_C_Tetrapod_high.png]

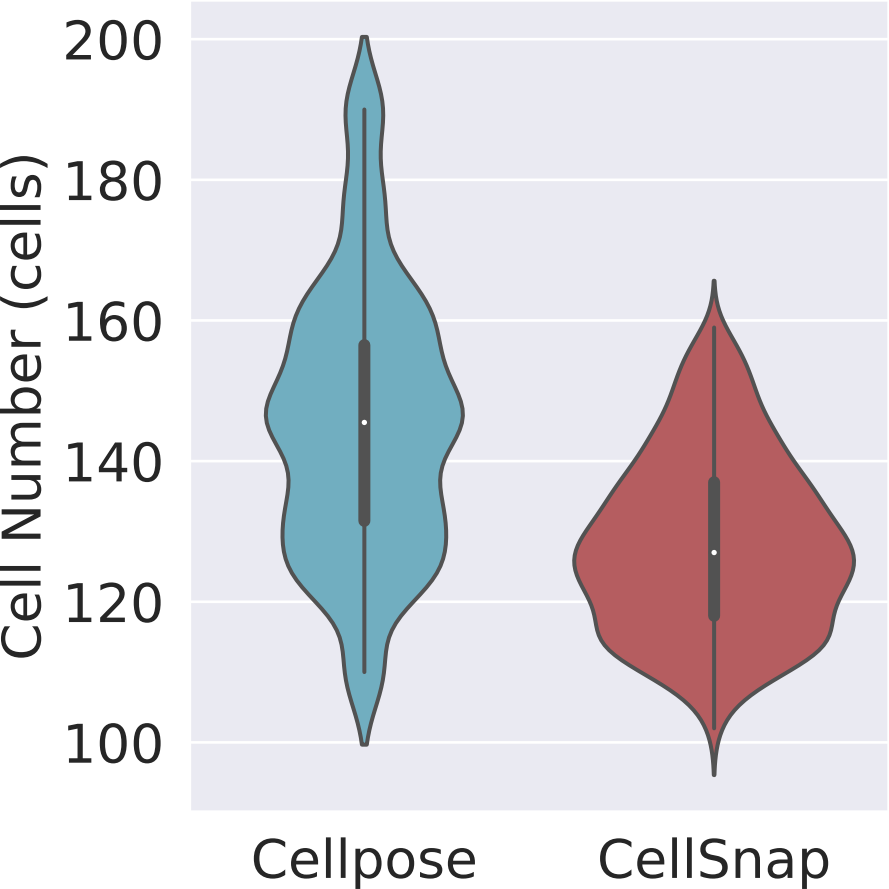

Supplement: Supplementary file 10 — Source Data [file 41467_2024_48502_MOESM10_ESM.zip › Main - Figure 5/SeparatePanels/Figure5_J.pdf]

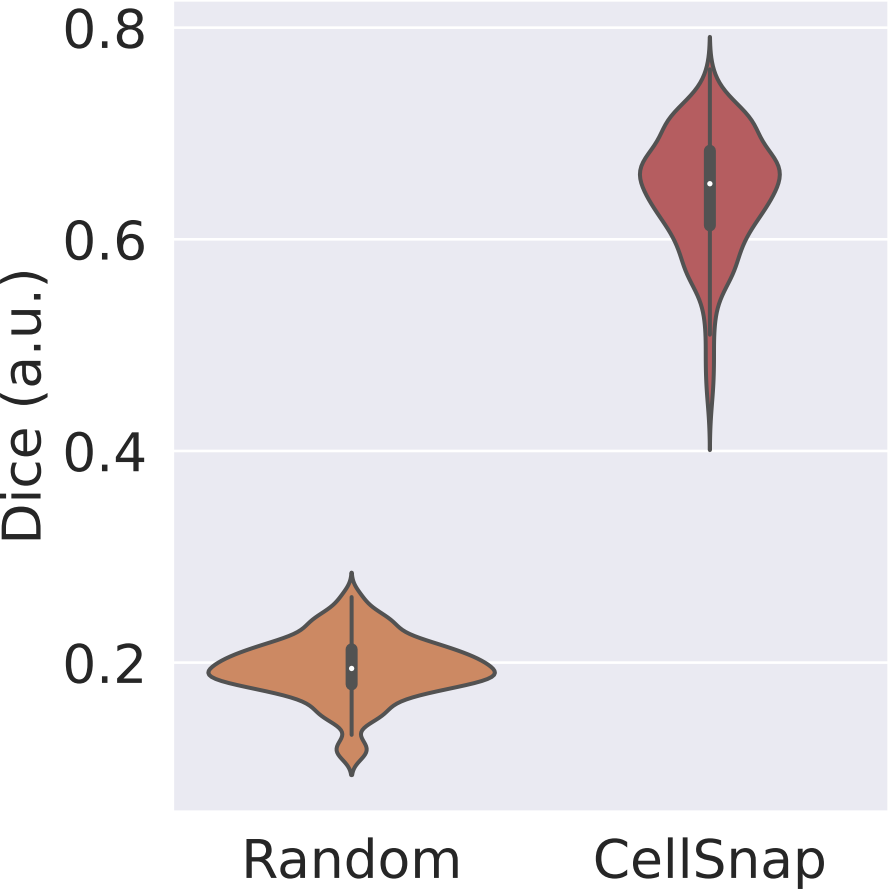

Supplement: Supplementary file 10 — Source Data [file 41467_2024_48502_MOESM10_ESM.zip › Main - Figure 5/SeparatePanels/Figure5_L.pdf]

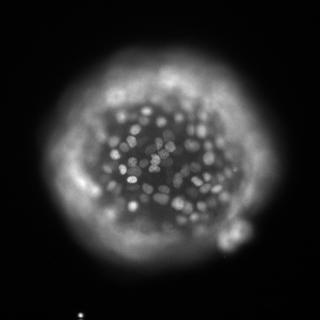

Supplement: Supplementary file 10 — Source Data [file 41467_2024_48502_MOESM10_ESM.zip › Main - Figure 4/SeparatePanels/Figure4_E_Standard_low.png]

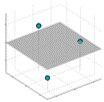

Supplement: Supplementary file 10 — Source Data [file 41467_2024_48502_MOESM10_ESM.zip › Main - Figure 1/Separate Panels/3D render 1D.png]

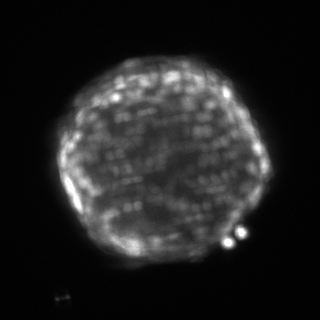

Supplement: Supplementary file 10 — Source Data [file 41467_2024_48502_MOESM10_ESM.zip › Main - Figure 4/SeparatePanels/Figure4_C_random_snapshot.png]

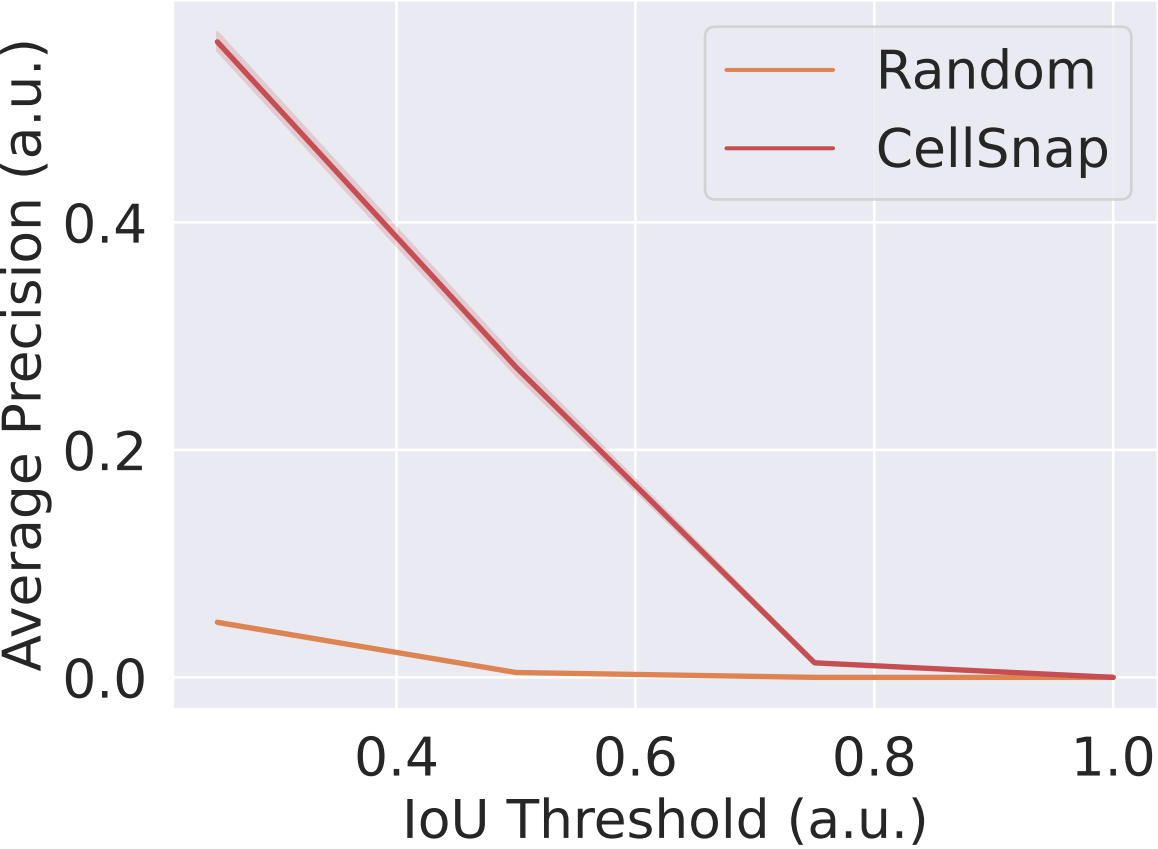

Supplement: Supplementary file 10 — Source Data [file 41467_2024_48502_MOESM10_ESM.zip › Main - Figure 5/SeparatePanels/Figure5_M.pdf]

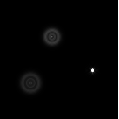

Supplement: Supplementary file 10 — Source Data [file 41467_2024_48502_MOESM10_ESM.zip › Main - Figure 1/Separate Panels/Standard 1E.png]

## Slide 1
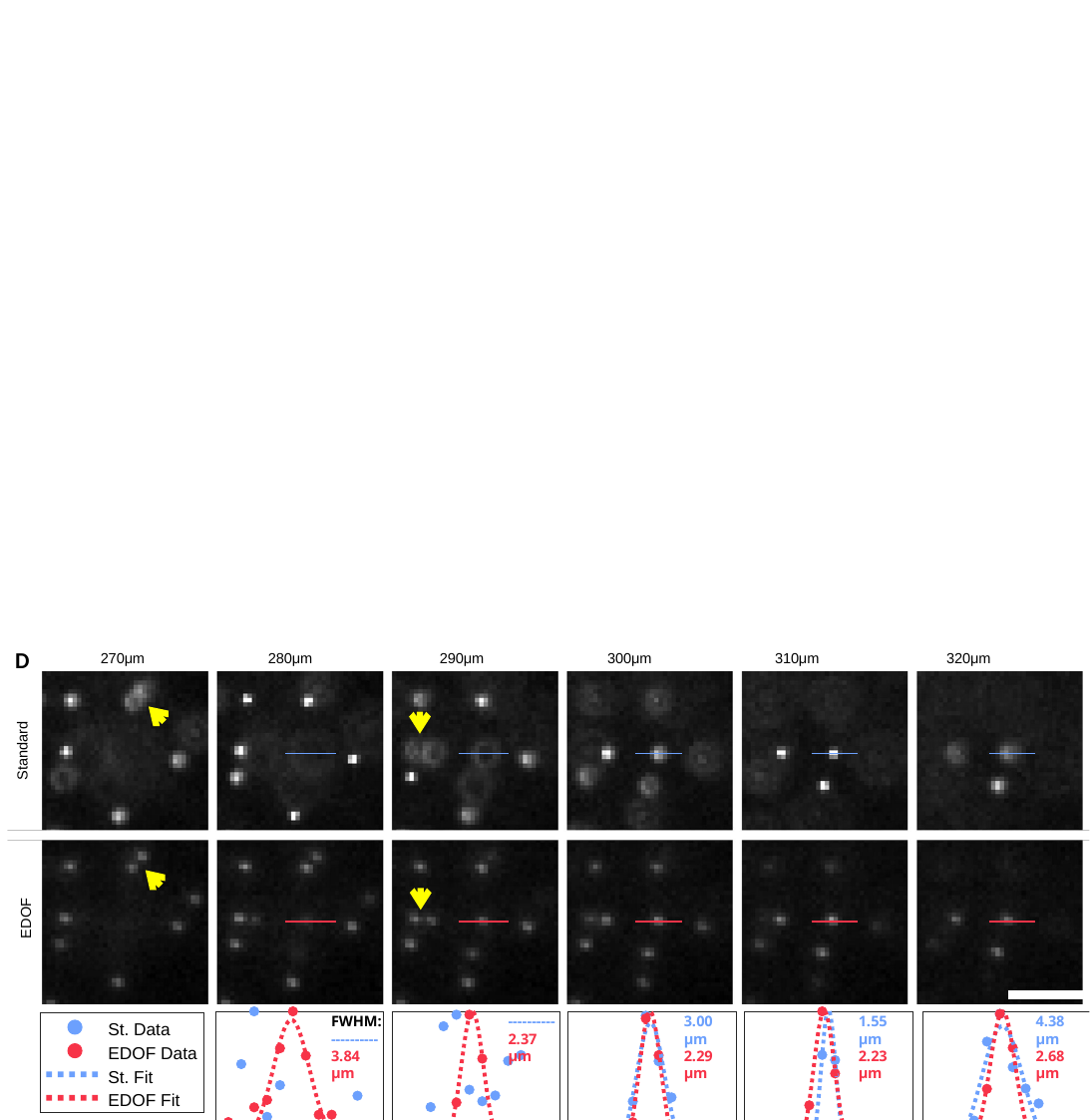

D
270μm 280μm 290μm 300μm 310μm 320μm
Standard
EDOF
FWHM:
----------
3.84 μm
----------
2.37 μm
3.00 μm
2.29 μm
1.55 μm
2.23 μm
4.38 μm
2.68 μm

Supplement: Supplementary file 10 — Source Data [file 41467_2024_48502_MOESM10_ESM.zip › Main - Figure 2/SeparatePanels/Fig 2D.pptx]

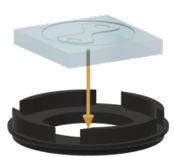

Supplement: Supplementary file 10 — Source Data [file 41467_2024_48502_MOESM10_ESM.zip › Main - Figure 1/Separate Panels/Tetrapod exploded 1B.png]

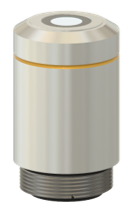

Supplement: Supplementary file 10 — Source Data [file 41467_2024_48502_MOESM10_ESM.zip › Main - Figure 1/Separate Panels/Objective 1B.png]

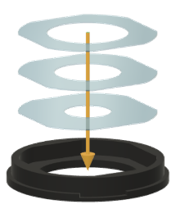

Supplement: Supplementary file 10 — Source Data [file 41467_2024_48502_MOESM10_ESM.zip › Main - Figure 1/Separate Panels/Exploded layers 1C.png]

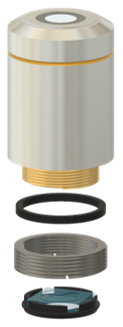

Supplement: Supplementary file 10 — Source Data [file 41467_2024_48502_MOESM10_ESM.zip › Main - Figure 1/Separate Panels/Full exploded 1B.png]

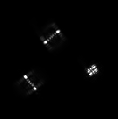

Supplement: Supplementary file 10 — Source Data [file 41467_2024_48502_MOESM10_ESM.zip › Main - Figure 1/Separate Panels/Tetrapod 1E.png]

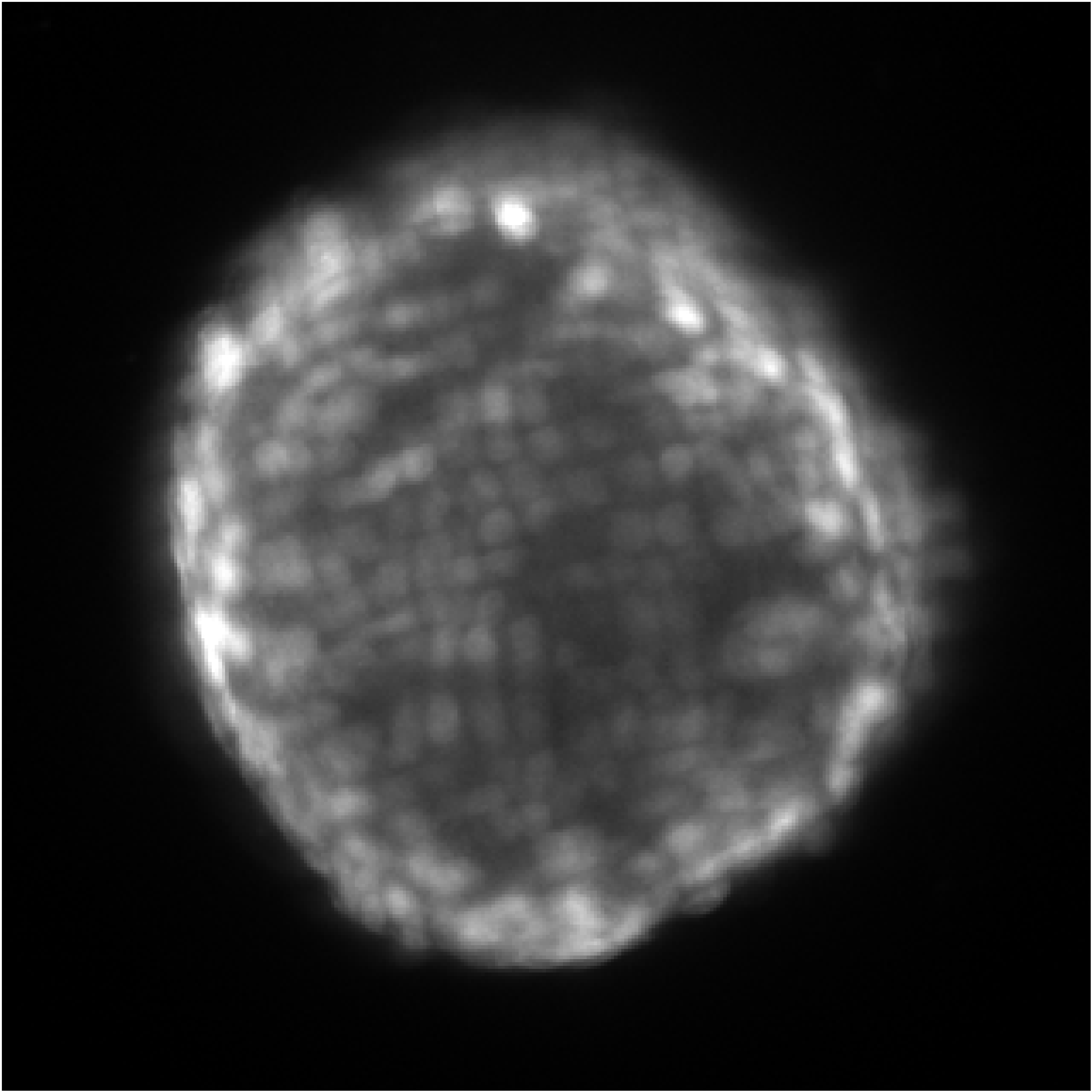

Supplement: Supplementary file 10 — Source Data [file 41467_2024_48502_MOESM10_ESM.zip › Main - Figure 5/SeparatePanels/Figure5_D.png]

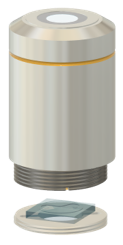

Supplement: Supplementary file 10 — Source Data [file 41467_2024_48502_MOESM10_ESM.zip › Main - Figure 1/Separate Panels/Objective 1A.png]

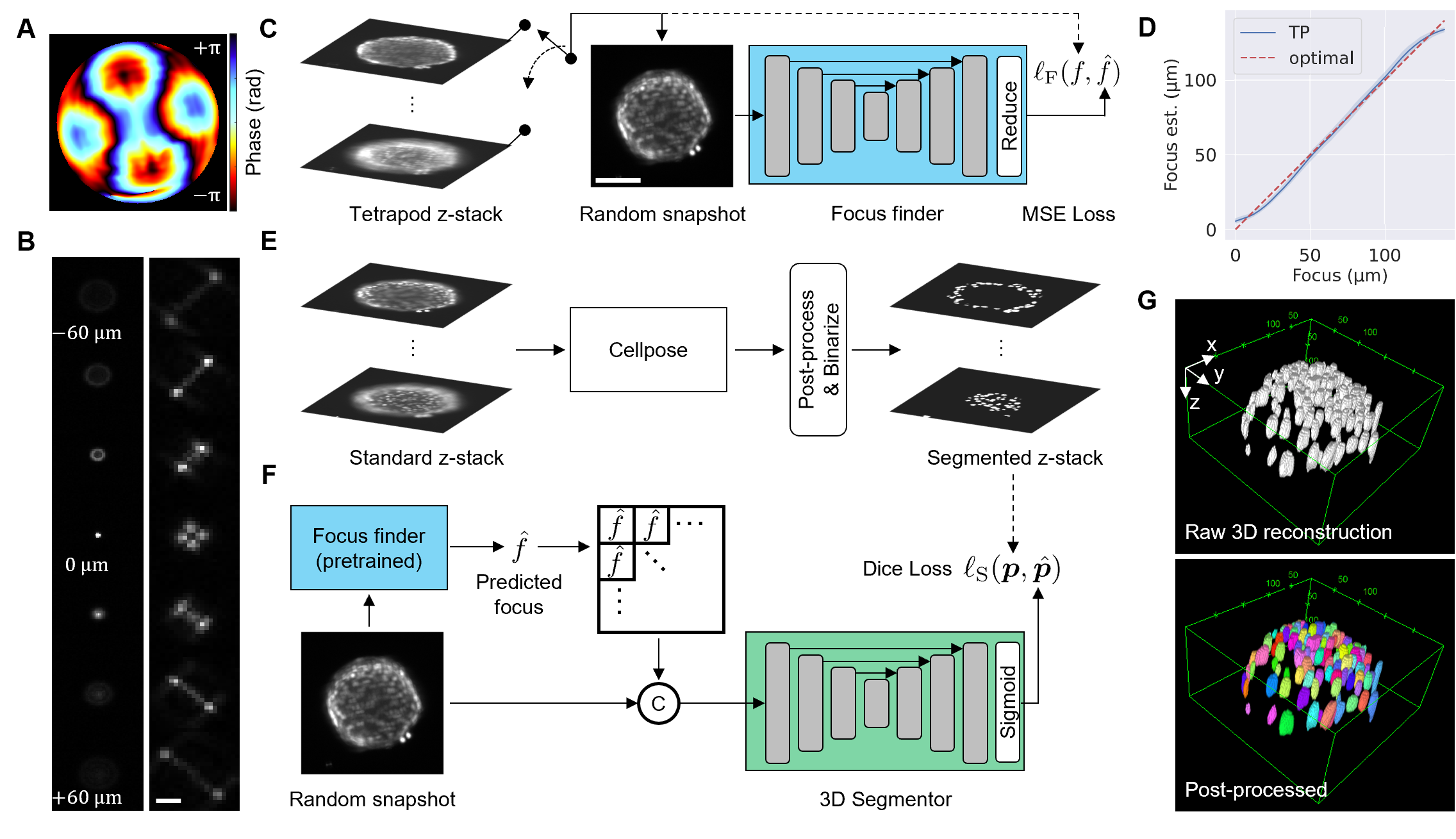

Supplement: Supplementary file 10 — Source Data [file 41467_2024_48502_MOESM10_ESM.zip › Main - Figure 4/CellSnap_architecture_and_concept.png]

## Slide 1
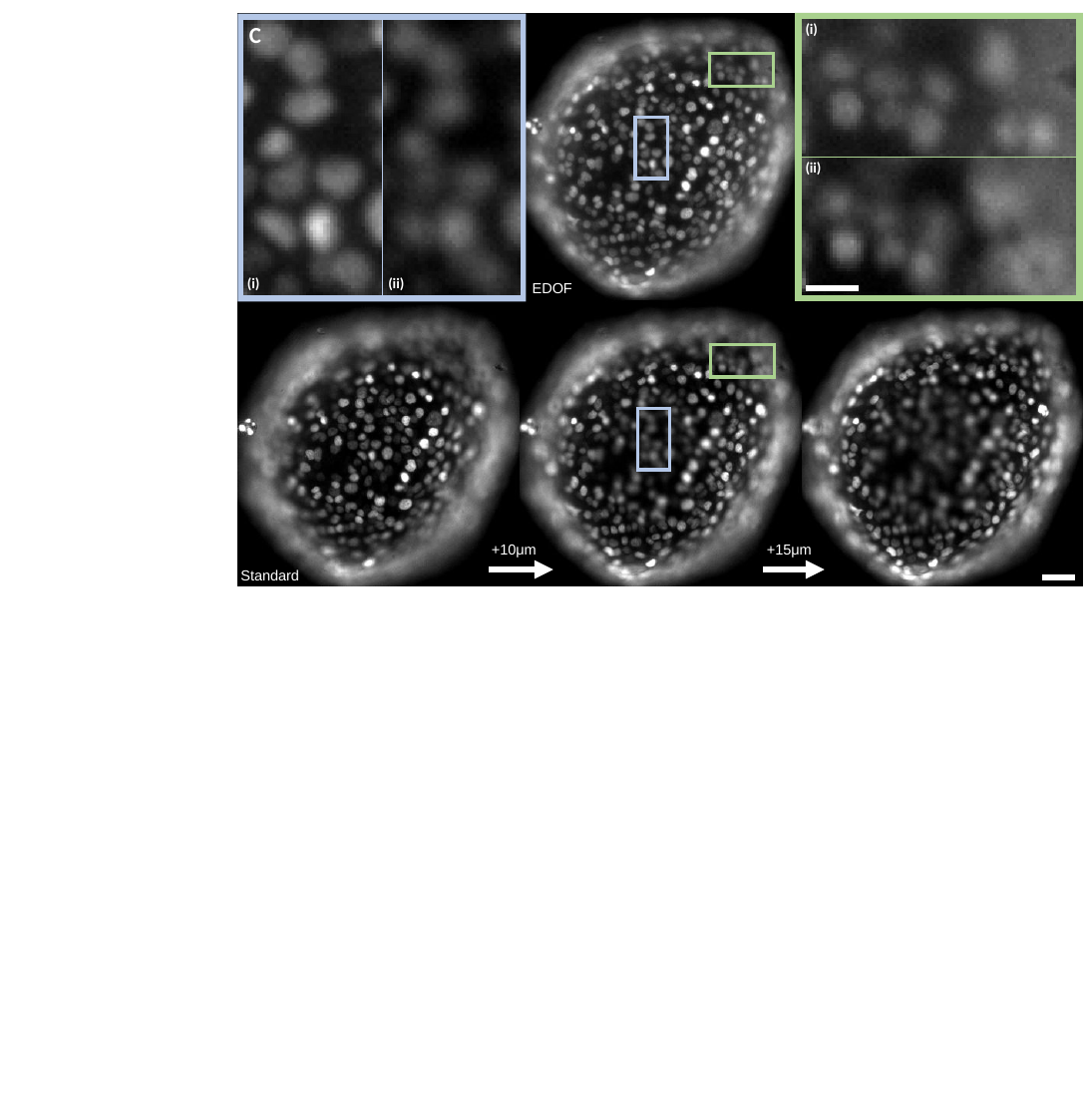

(i)
C
(ii)
(i)
(ii)
EDOF
+10μm
+15μm
Standard

Supplement: Supplementary file 10 — Source Data [file 41467_2024_48502_MOESM10_ESM.zip › Main - Figure 3/SeparatePanels/Fig3C.pptx]

## Slide 1
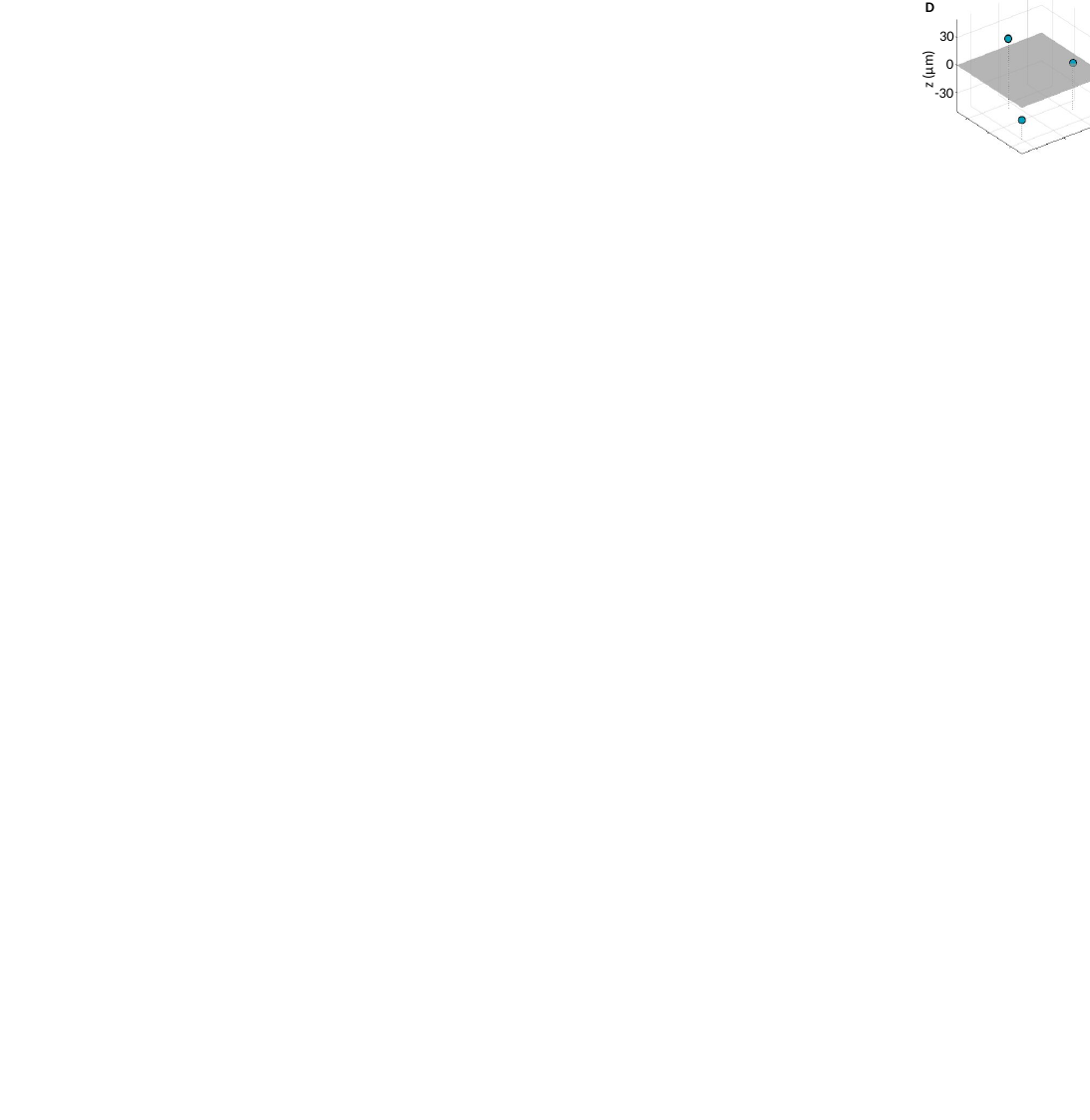

D
30
0
z (μm)
-30

Supplement: Supplementary file 10 — Source Data [file 41467_2024_48502_MOESM10_ESM.zip › Main - Figure 1/Separate Panels/Fig 1D.pptx]

## Slide 1
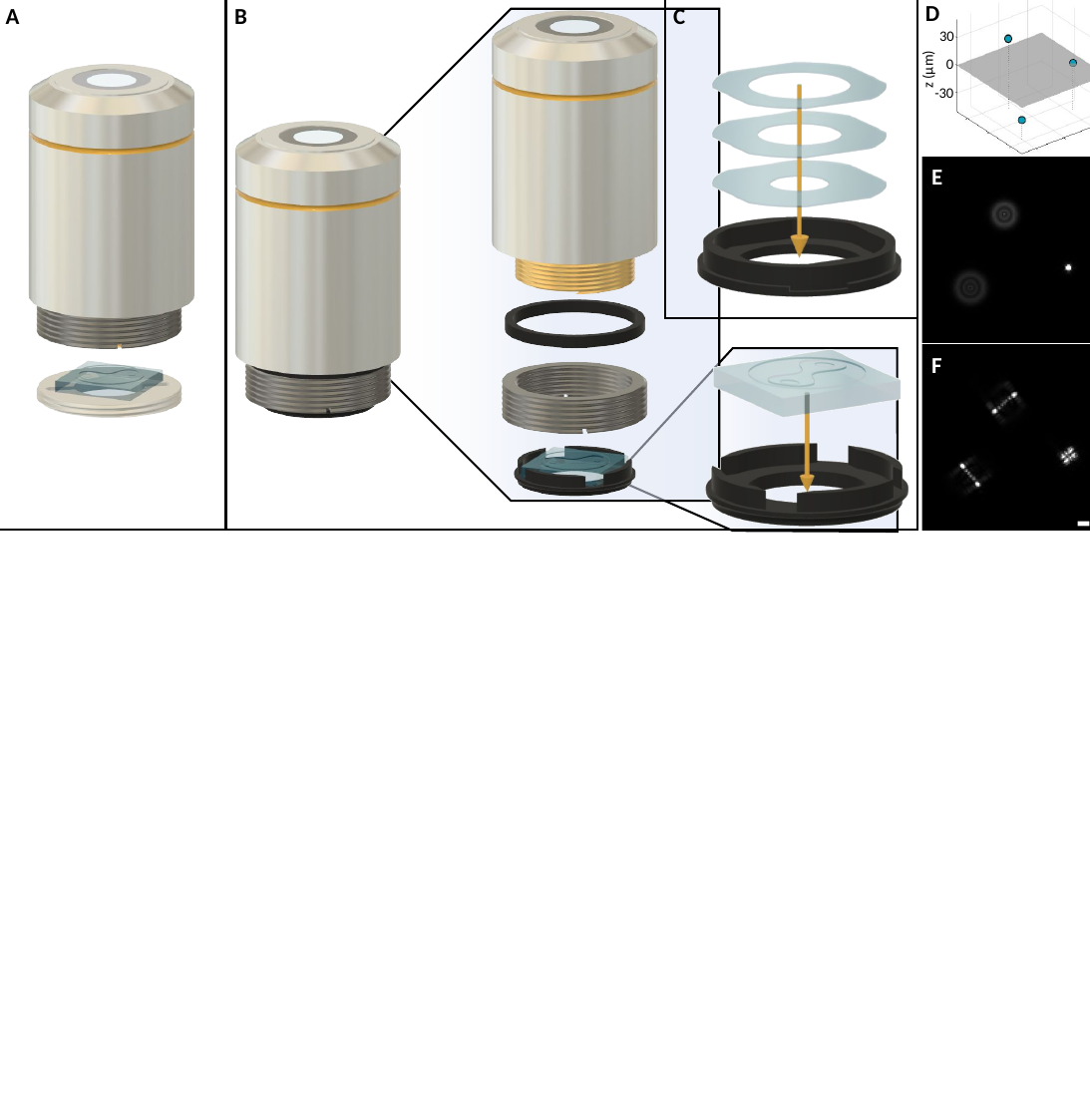

D
A
B
C
30
0
z (μm)
-30
c
E
F

Supplement: Supplementary file 10 — Source Data [file 41467_2024_48502_MOESM10_ESM.zip › Main - Figure 1/Figure 1_PPT.pptx]

## Slide 1
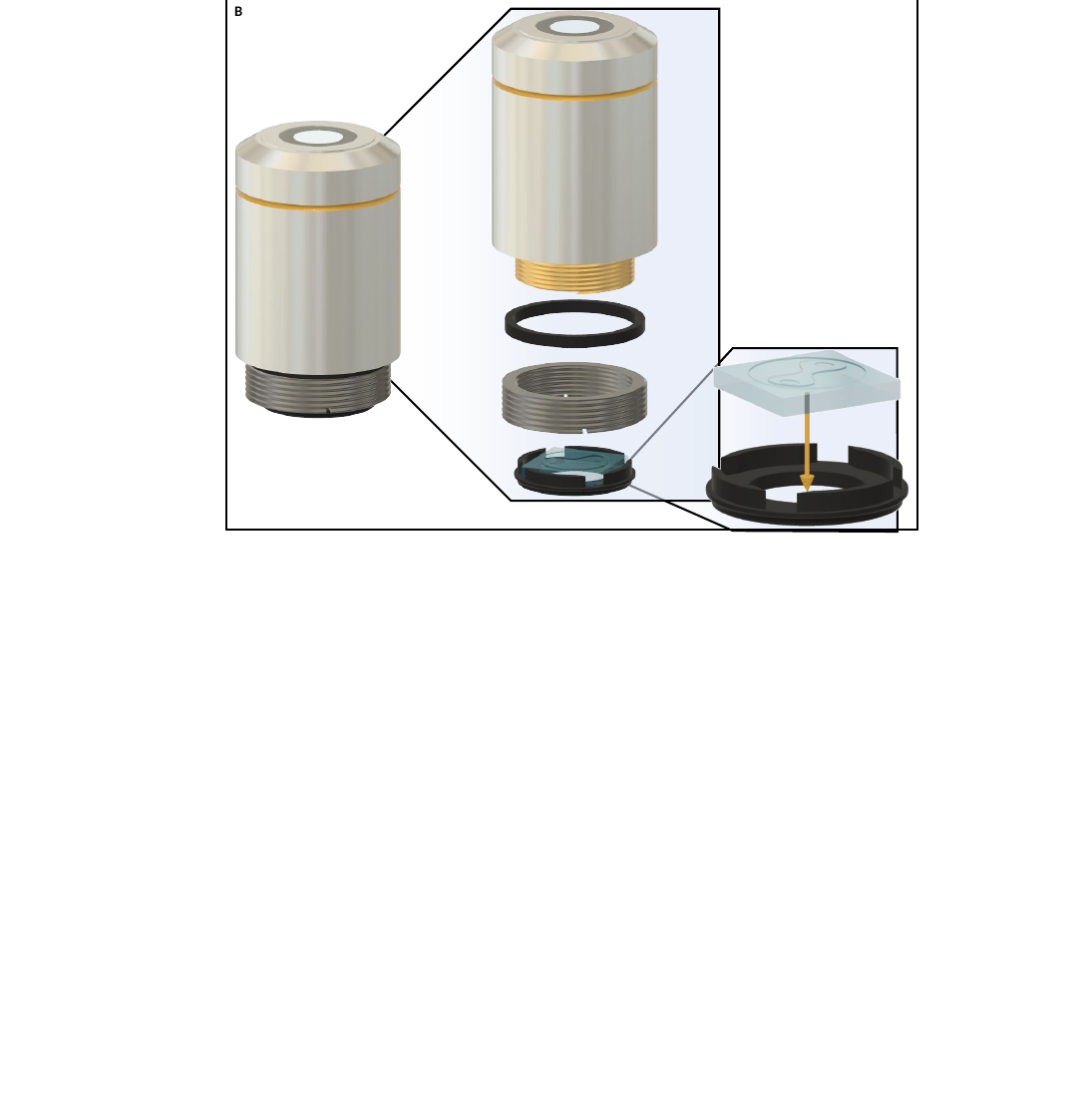

B
c

Supplement: Supplementary file 10 — Source Data [file 41467_2024_48502_MOESM10_ESM.zip › Main - Figure 1/Separate Panels/Fig 1B.pptx]

## Slide 1
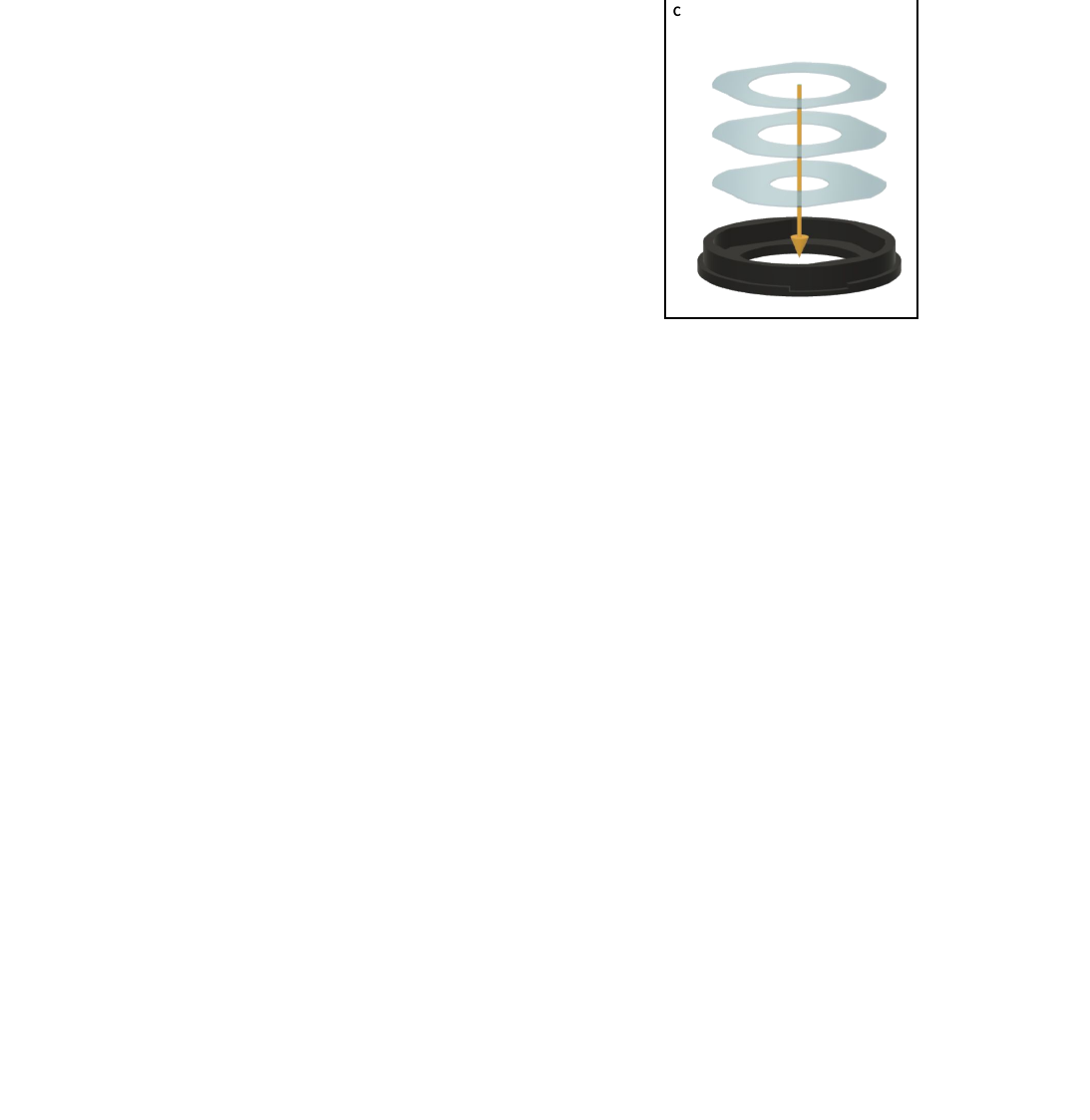

C

Supplement: Supplementary file 10 — Source Data [file 41467_2024_48502_MOESM10_ESM.zip › Main - Figure 1/Separate Panels/Fig 1C.pptx]

## Slide 1
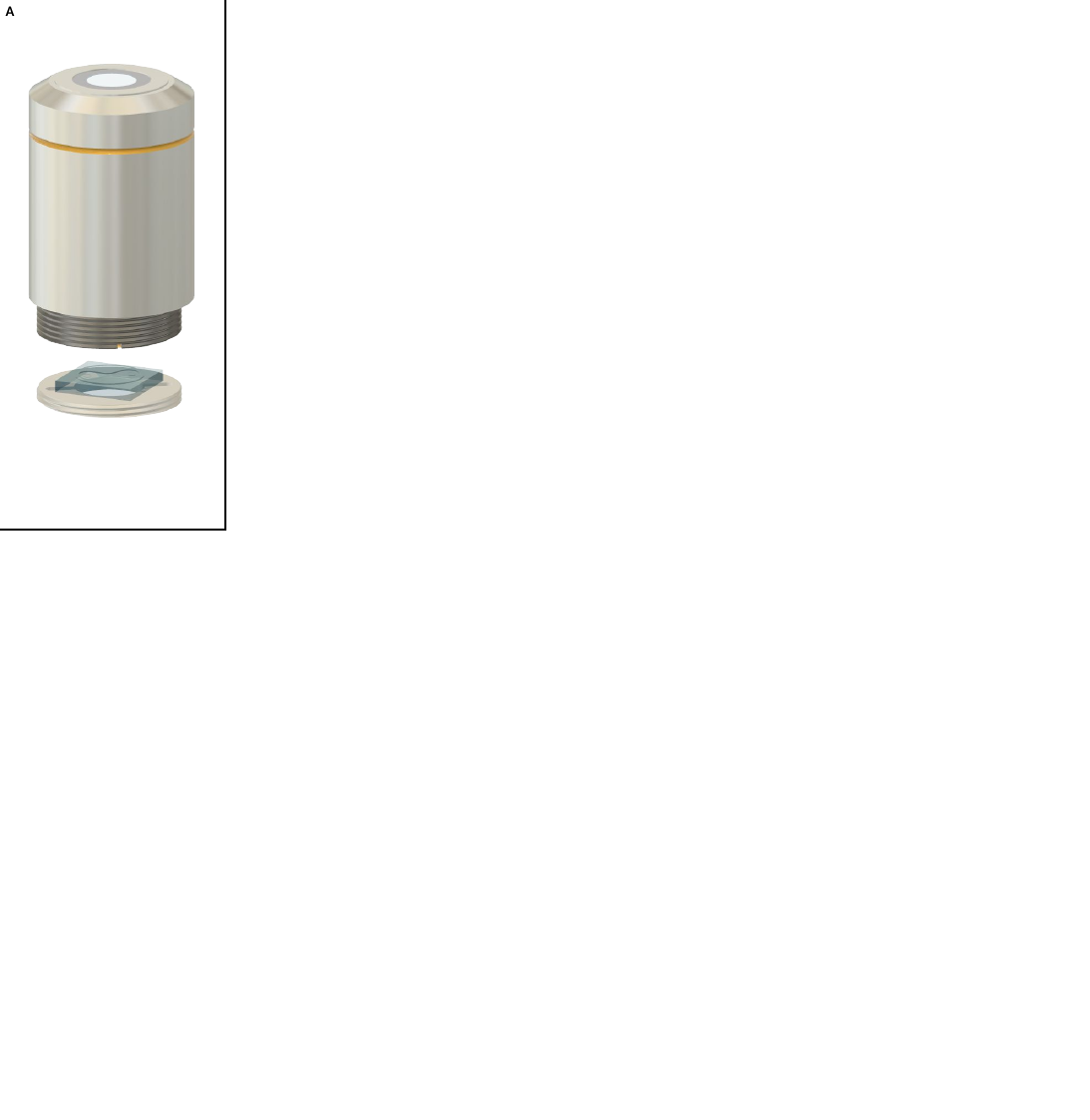

A

Supplement: Supplementary file 10 — Source Data [file 41467_2024_48502_MOESM10_ESM.zip › Main - Figure 1/Separate Panels/Fig 1A.pptx]

## Slide 1
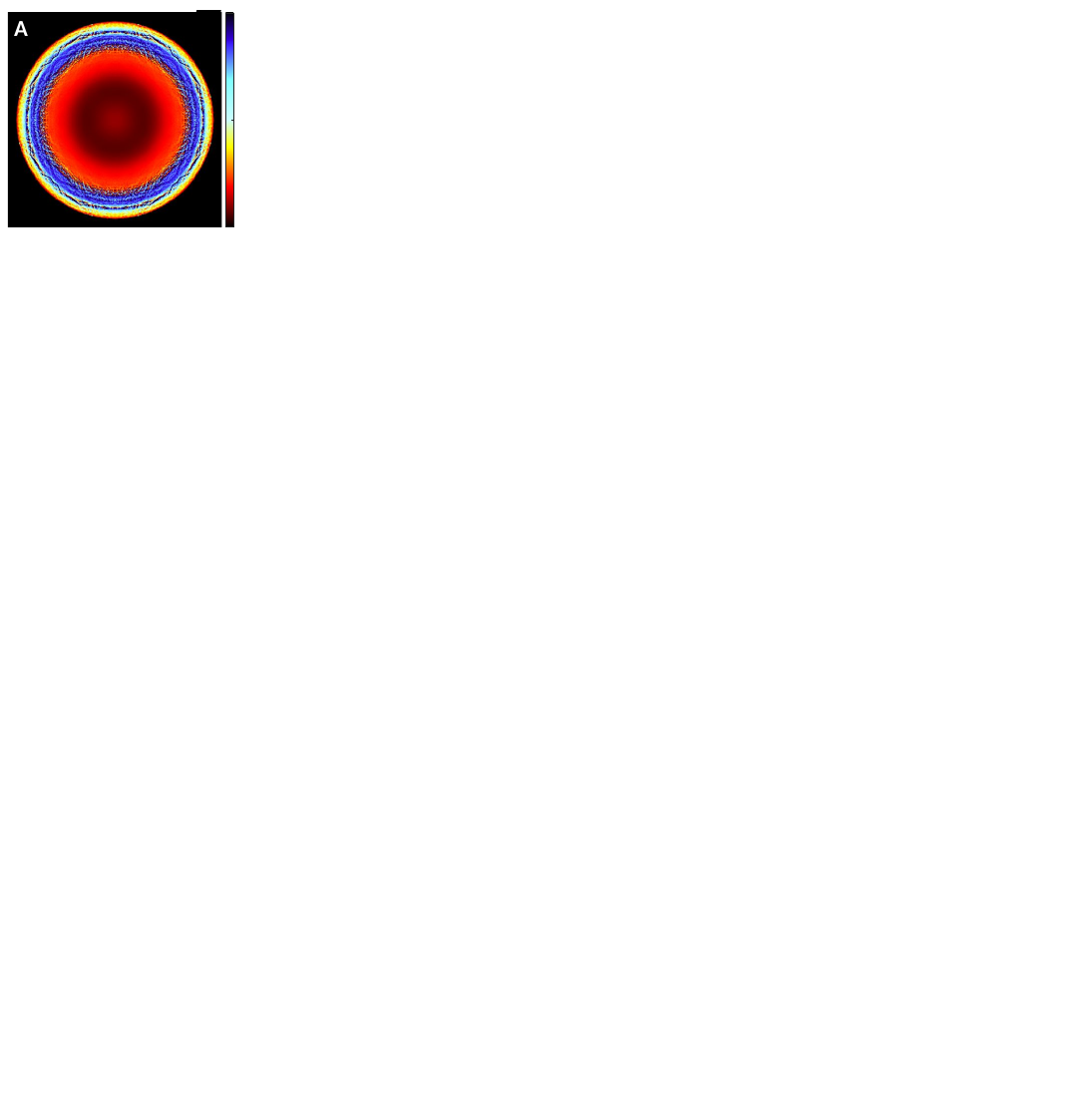

A
C
B

Supplement: Supplementary file 10 — Source Data [file 41467_2024_48502_MOESM10_ESM.zip › Main - Figure 3/SeparatePanels/Fig3A.pptx]

## Slide 1
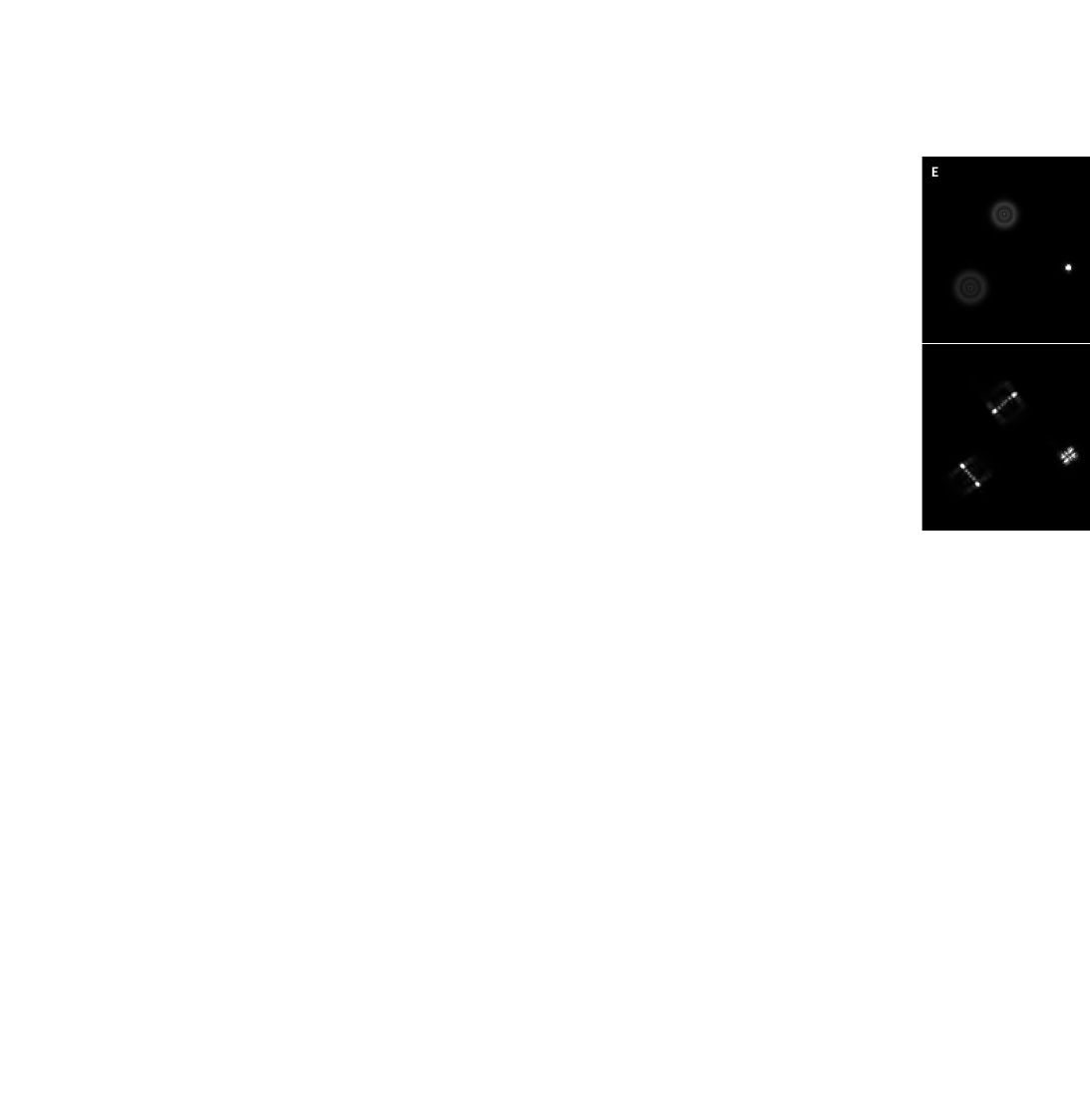

E

Supplement: Supplementary file 10 — Source Data [file 41467_2024_48502_MOESM10_ESM.zip › Main - Figure 1/Separate Panels/Fig 1E.pptx]

## Slide 1
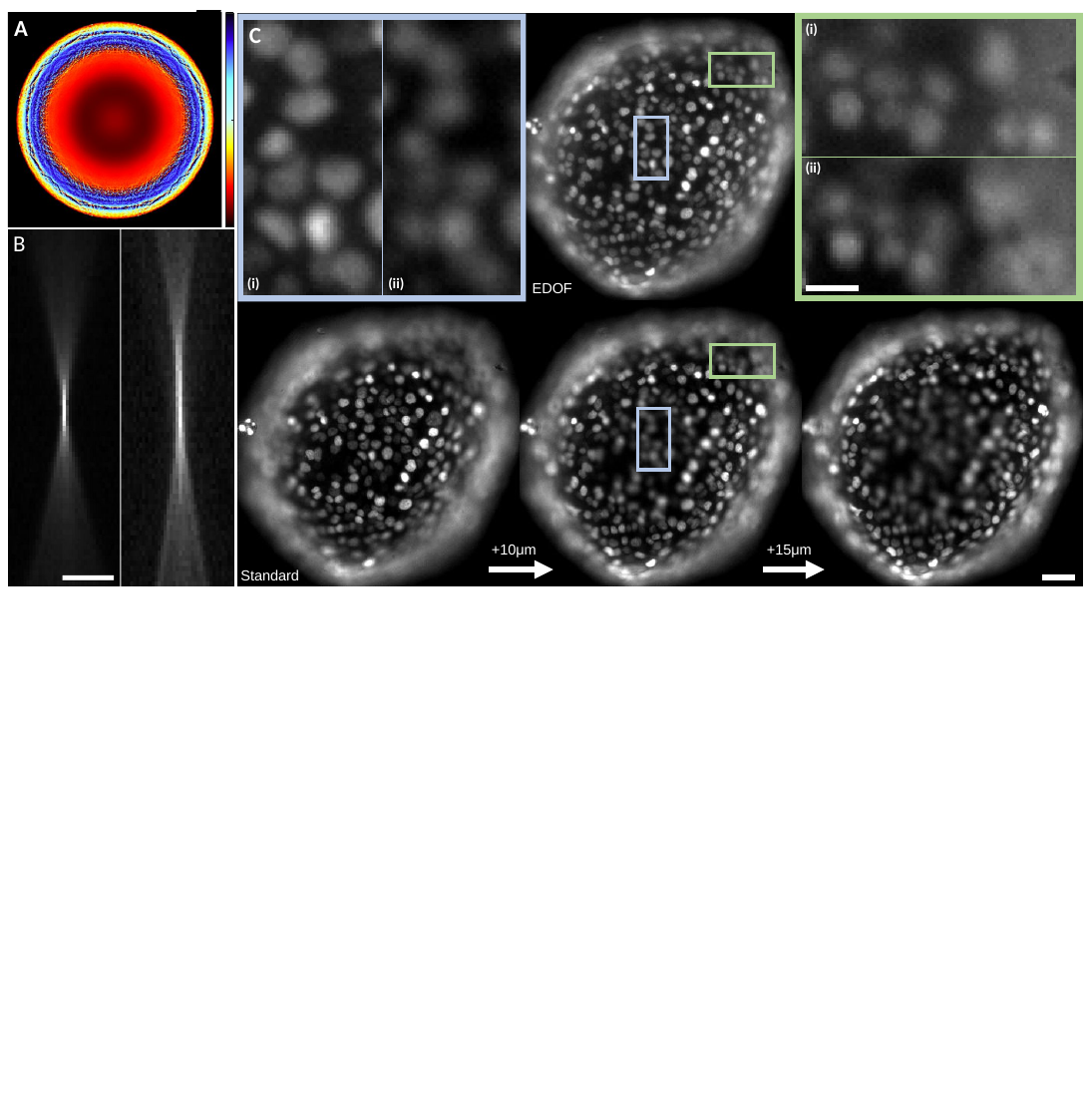

A
(i)
C
(ii)
B
(i)
(ii)
EDOF
+10μm
+15μm
Standard

Supplement: Supplementary file 10 — Source Data [file 41467_2024_48502_MOESM10_ESM.zip › Main - Figure 3/Fig3.pptx]

## Slide 1
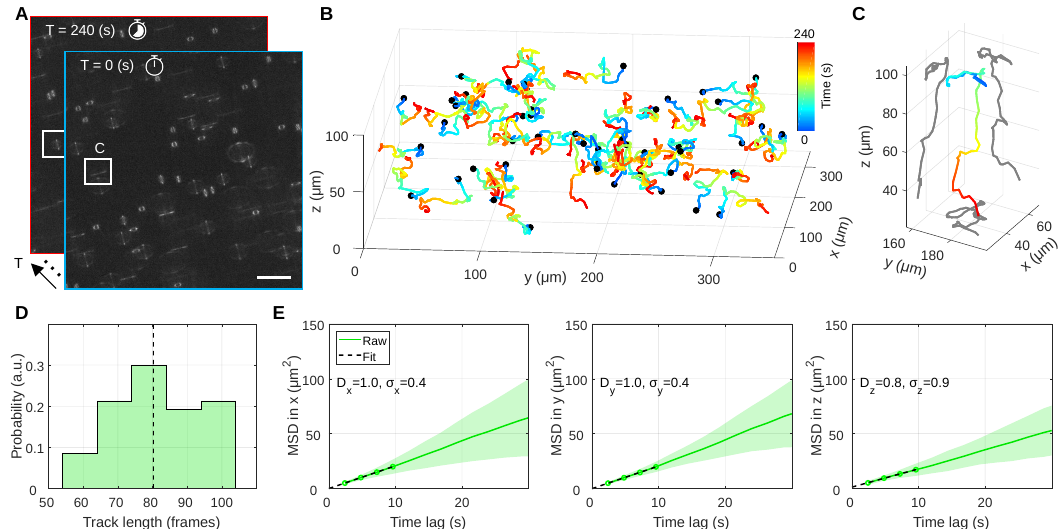

A
B
C
T = 240 (s)
T = 0 (s)
T
…
C
240
Time (s)
0
D
E

Supplement: Supplementary file 10 — Source Data [file 41467_2024_48502_MOESM10_ESM.zip › Main - Figure 6/NTA_and_3D_tracking.pptx]
